# Diagnostic Accuracy of Machine and Deep Learning Methods in Intracranial Hemorrhage Detection: A Systematic Review and Meta-Analysis

**DOI:** 10.3390/diagnostics16213516

**Published:** 2026-10-27

**Authors:** Anastasis Alexopoulos, Yiheng Tan, Maarten Uyttenboogaart, Mathias Prokop, Ronald J. H. Borra, Maarten M. H. Lahr, Reinoud P. H. Bokkers

**Affiliations:** 1 Department of Radiology, University Medical Center Groningen, University of Groningen, 9700 AB Groningen, The Netherlands; 2 Department of Neurology, University Medical Center Groningen, University of Groningen, 9700 AB Groningen, The Netherlands; 3 Department of Imaging, Radboud University Medical Center, 6500 HB Nijmegen, The Netherlands; 4 Department of Nuclear Medicine and Molecular Imaging, University Medical Center Groningen, University of Groningen, 9700 AB Groningen, The Netherlands; 5 Department of Epidemiology, University Medical Center Groningen, University of Groningen, 9700 AB Groningen, The Netherlands

**Keywords:** intracranial hemorrhage, stroke, machine learning, deep learning

## Abstract

**Background/Objectives**: In this systematic review we investigated the diagnostic accuracy of machine and deep learning algorithms for the detection of intracranial hemorrhage (ICH) and its subtypes such as subarachnoid, intraventricular, intraparenchymal, subdural and epidural hemorrhage. In addition, we investigated their generalizability by comparing methods tested on private vs. public datasets, and FDA- vs. non-FDA-approved algorithms. **Methods**: PubMed, Scopus and Embase were searched between January 2010 and December 2025. Eligible studies included patients with acute neurological deficit that received non-contrast CT head scans and implemented machine and deep learning algorithms for ICH detection, that were tested on private and/or public datasets, received FDA approval or not, and used clinicians’ evaluation as reference standard. Pooled sensitivity and specificity were incorporated for meta-analysis. **Results**: A total of 89 publications received full assessment and 51 studies were included in the meta-analysis. The pooled sensitivity and specificity for ICH detection were 94.5% (95% CI: 92.4–96.0) and 95.3% (95% CI: 93.1–96.9), respectively. For AI methods tested on private and public datasets, the pooled sensitivity reached 94.4% (95% CI: 91.2–96.5) and 94.5% (95% CI: 91.8–96.4), with a pooled specificity in 95.6% (95% CI: 92.9–97.2) and 95.1% (95% CI: 90.1–97.5), respectively. FDA- and non-FDA-approved AI algorithms showed a pooled sensitivity of 90.7% (95% CI: 87.1–93.4) and specificity of 95.8% (95% CI: 90.3–98.3) in the former group, and 95.5% (95% CI: 93.4–96.9) and 95.7% (95% CI: 92.4–97.6) for the latter group. **Conclusions**: Study results demonstrated that the diagnostic accuracy of the developed AI algorithms for ICH detection is equivalent to that of experienced clinicians. Methods tested on private and public datasets, and FDA- vs. non-FDA-approved, differ slightly in ICH diagnosis, showing encouraging signs for their incorporation in decision support systems in stroke patient pathways.

## 1. Introduction

Intracranial hemorrhage (ICH) is a life-threatening neurological condition that accounts for approximately 28% of all stroke events worldwide, with a global incidence of 44 cases per 100,000 people. It significantly contributes to stroke-related mortality and disability, underscoring the need for prompt and accurate diagnosis to facilitate timely treatment [1,2,3]. Acute neurological deficits caused by ICH demand urgent medical attention within a narrow therapeutic window to optimize outcome.

Differentiating ICH from ischemic stroke is critical, as management strategies differ significantly. While clinical evaluation alone is insufficient, non-contrast computed tomography (NCCT) remains the cornerstone of ICH diagnosis due to its availability and ability to rapidly visualize brain pathology [4]. However, subtle radiological findings such as small hemorrhages or hyperdense vessel signs can be challenging to detect, particularly for less experienced radiologists. This issue is exacerbated in resource-limited settings or outside regular working hours, where access to specialized neuro-radiologists may be constrained.

Over the past decade, artificial intelligence (AI), particularly machine learning (ML) and deep learning (DL) techniques, has emerged as a promising tool in the healthcare industry. These technologies offer the potential to enhance diagnostic accuracy, assist radiologists in interpreting scans more efficiently, and even operate as standalone diagnostic systems. FDA- and CE-approved AI tools have catalyzed the integration of AI into clinical workflows, demonstrating potential in emergency and routine settings [5,6]. However, the generalizability and reliability of these algorithms remain contingent on various factors, including the quality of training datasets, imaging protocols, and scanner-specific characteristics.

The main aim of this systematic review and meta-analysis is to evaluate the diagnostic accuracy of ML and DL algorithms in detecting ICH and its subtypes (subarachnoid hemorrhage, intraparenchymal hemorrhage, intraventricular hemorrhage, subdural hematoma, and epidural hematoma). As a secondary objective, we assess the generalizability of the developed methods by comparing the performance of the algorithms tested on private datasets with the ones tested on public datasets, and examine the diagnostic performance of FDA-approved versus non-FDA-approved methods.

## 2. Materials and Methods

This systematic review was conducted following the guidelines outlined in the Preferred Reporting Items for Systematic reviews and Meta-Analysis (PRISMA) statement [7].

### 2.1. Literature Search

PubMed, Scopus and Embase were searched for eligible studies that examined artificial intelligence algorithms’ performance on the detection of non-traumatic intracranial hemorrhage from emergency non-contrast CT scans of adult cohorts. Studies with post-operative, chronic, incidental or follow-up CT scans were excluded. The search spanned from January 2010 to December 2025 and included a combination of technical (artificial intelligence, machine learning, etc.), clinical (intracranial, intracerebral hemorrhage, stroke, etc.), task-related (classification, detection) and imaging (computed tomography, non-contrast, head CT, etc.) terms. The search used free text terms, Medical Subject Headings terms, and Boolean operators. The detailed search is provided in Appendix A. Additional studies were identified by cross-reference checks of the relevant literature in each reference.

### 2.2. Study Selection

Two reviewers (A.A. and Y.T.) independently screened the titles and abstracts of retrieved studies for eligibility, with any conflicts resolved by a third reviewer (R.P.H.B.). Full-text studies were then evaluated using predefined inclusion criteria: (a) the study employed ML or DL methods; (b) algorithms were used for the detection or classification of ICH on NCCT scans; (c) performance metrics of the AI tools were compared with clinical assessments or expert evaluations; (d) the study was peer-reviewed. Studies that did not report all the required information on sensitivity and specificity metrics and/or on the number of CTs in test datasets were included only in the systematic review. Case reports, reviews, commentaries, letters to the editors, conference abstracts and publications not written in English were excluded.

### 2.3. Data Extraction

Data were extracted from eligible studies using the Checklist for Artificial Intelligence in Medical Imaging (CLAIM) [8] tool, to gather those variables that describe the author’s work in sufficient detail. Information collected from each study included the study design (prospective, retrospective), the type of ICH detected, the size and type of test datasets (private, public), the reference standard (clinical reports, expert consensus), the AI algorithm tested, the algorithms’ regulatory approval status (FDA, non-FDA) and its diagnostic accuracy. The extracted information was also used to evaluate the methodological quality of the included studies, allowing for a structured assessment of study design, reporting completeness, and potential sources of bias across eligible publications.

### 2.4. Study Subgrouping

Apart from the main group of studies which comprised all the publications that fulfilled the inclusion criteria, two additional subgroups were constructed based on whether the developed ML and DL algorithms were tested on publicly available datasets (private vs. public datasets), and whether the AI tools had acquired a commercial license (FDA vs. non-FDA approved).

Private datasets often lack detailed information about patient population characteristics and imaging protocols, which may hinder the performance of AI algorithms in real-world settings. Comparing AI models evaluated on private datasets with those tested on public datasets provides insight into potential differences in diagnostic performance and offers an indication of the models’ generalizability. Similarly, comparing FDA-approved AI tools with algorithms that have not received regulatory clearance enables the assessment of potential differences in ICH detection performance associated with market approval.

### 2.5. Meta-Analysis

A meta-analysis was conducted in R Studio (version 2026.01.0) with the goal of calculating the pooled performance metrics for ICH and detection of its subtypes. After grouping all included studies according to the type of detection task (ICH, SAH, IPH, IVH, SDH, EDH), according to the type of dataset the AI tools were tested on (private or public), and according to their commercial license (FDA- vs. non-FDA-approved), the pooled sensitivity and specificity of the algorithms included in each subgroup were calculated.

A bivariate random-effects model was used for each subgroup because methodological heterogeneity across the included studies was anticipated, including differences in patient population, dataset size, imaging protocols, model design, and reference standards. This model assumes that the true effect size may vary between studies and therefore incorporates both within-study sampling error and between-study variance. Therefore, the pooled estimate should be interpreted as the average effect across the included studies.

The bivariate random-effect models developed applied a logit transformation to the sensitivity and specificity metrics of each study, and used the rma.glmm function of the metafor package (version 5.0-1) with a restricted maximum-likelihood estimator (REML) estimator. Threshold effects were tested from the between-study correlation of logit sensitivity and specificity, zero cells were handled through the binomial glmm function, and confidence intervals are reported along with the studies’ heterogeneity. Addition information for the meta-analysis about the number of studies in each subgroup is presented in the Supplementary Materials.

## 3. Results

**Figure 1:**
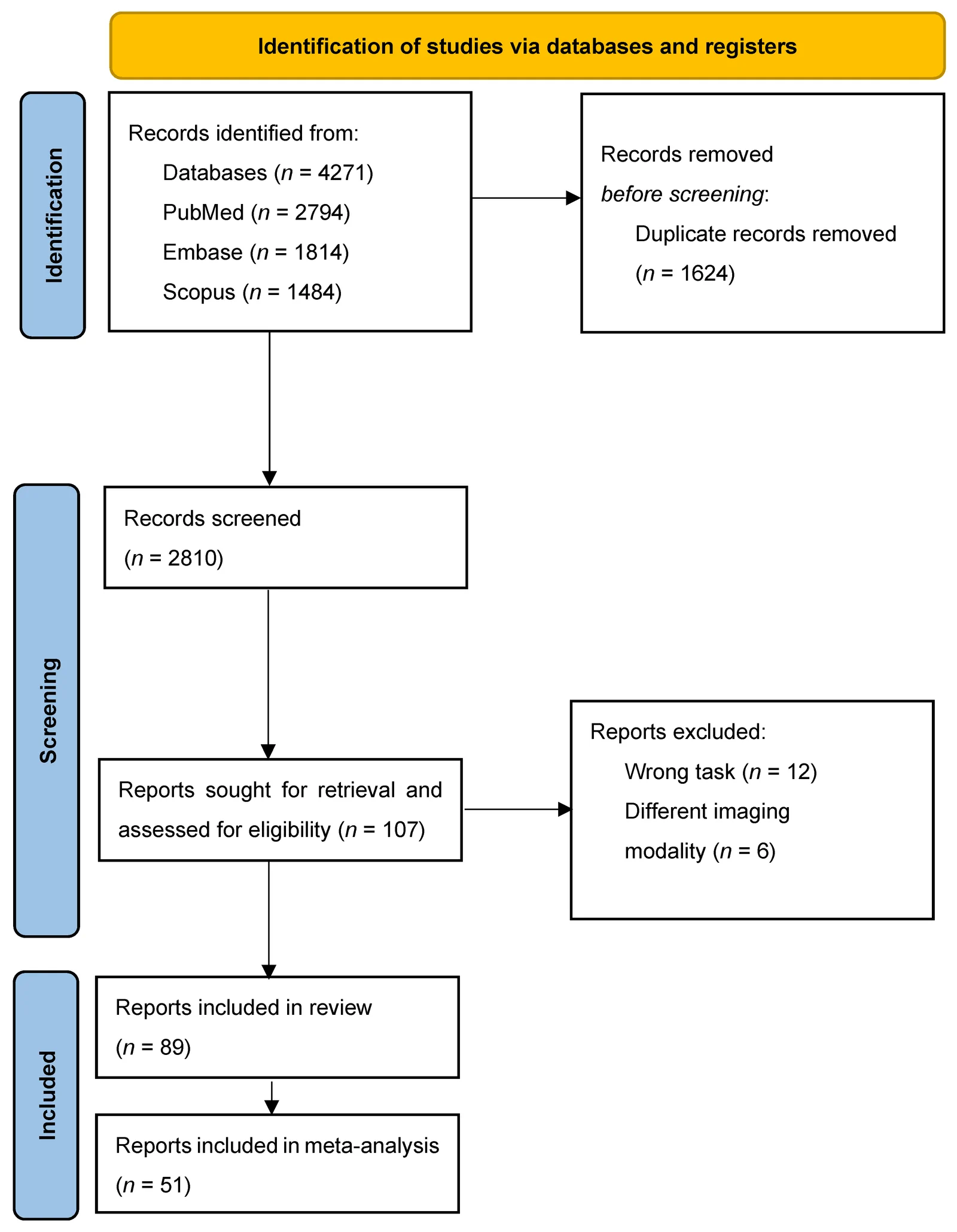
PRISMA flowchart for selection process.

After screening titles and abstracts, 107 full-text articles were assessed for eligibility, and 89 studies were included in the systematic review. Of these, 51 studies provided sufficient data for inclusion in the meta-analysis. A flowchart of the study process is shown in Figure 1.

### 3.1. Study Characteristics

The main characteristics of the 89 included studies are summarized in Table 1. Among these, 32 studies focused exclusively on the detection of intracranial hemorrhage (ICH), while 29 investigated both ICH and its subtypes. Six studies specifically targeted SAH, and one focused on IVH. DL methods were the most commonly used, appearing in 72 studies, while 8 studies employed a combination of ML and DL techniques and 9 only ML architecture.

### 3.2. Study Quality Assessment

**Figure 2:**
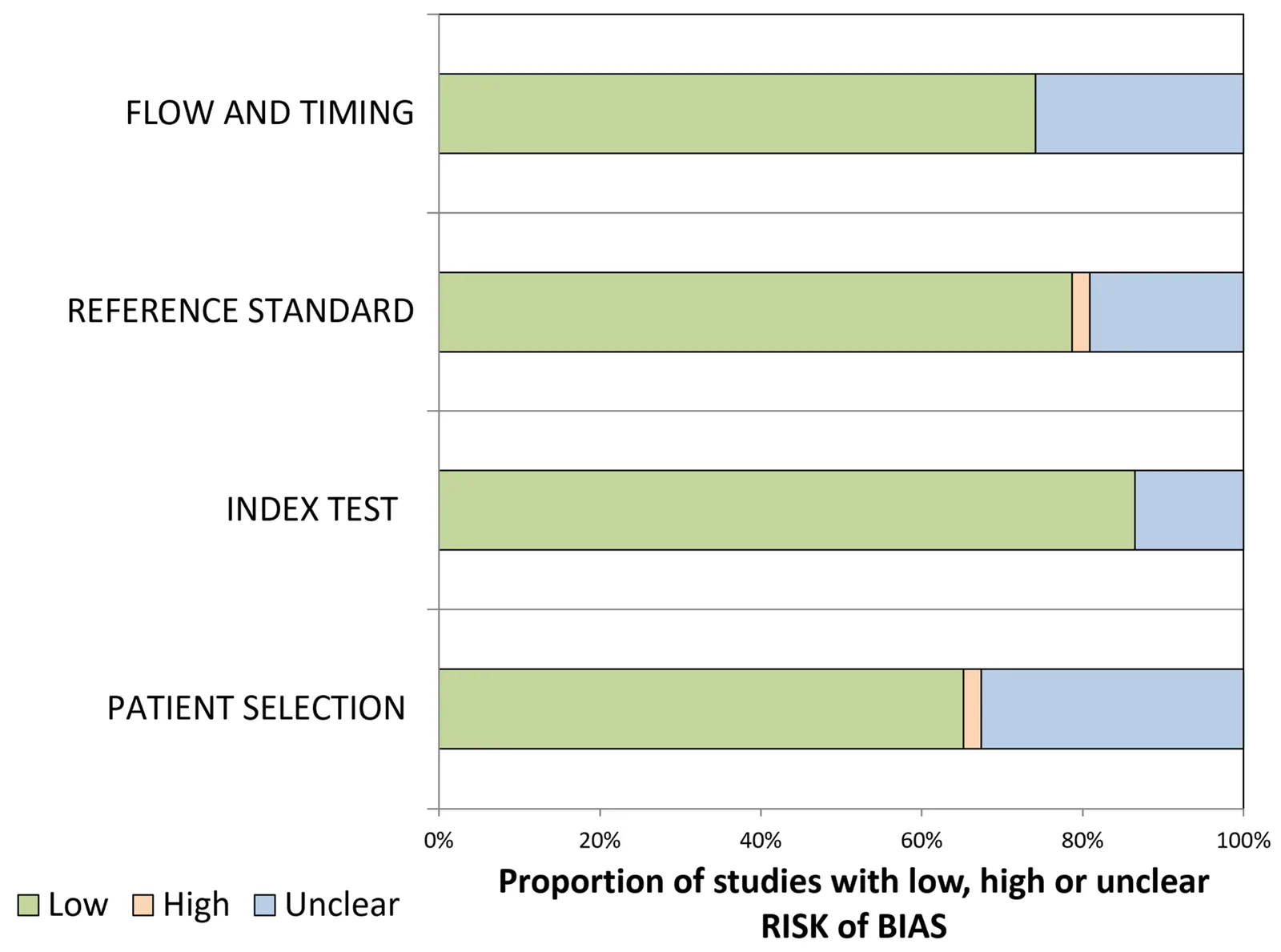
Risk of bias assessment of the included studies according to QUADAS-2.

**Figure 3:**
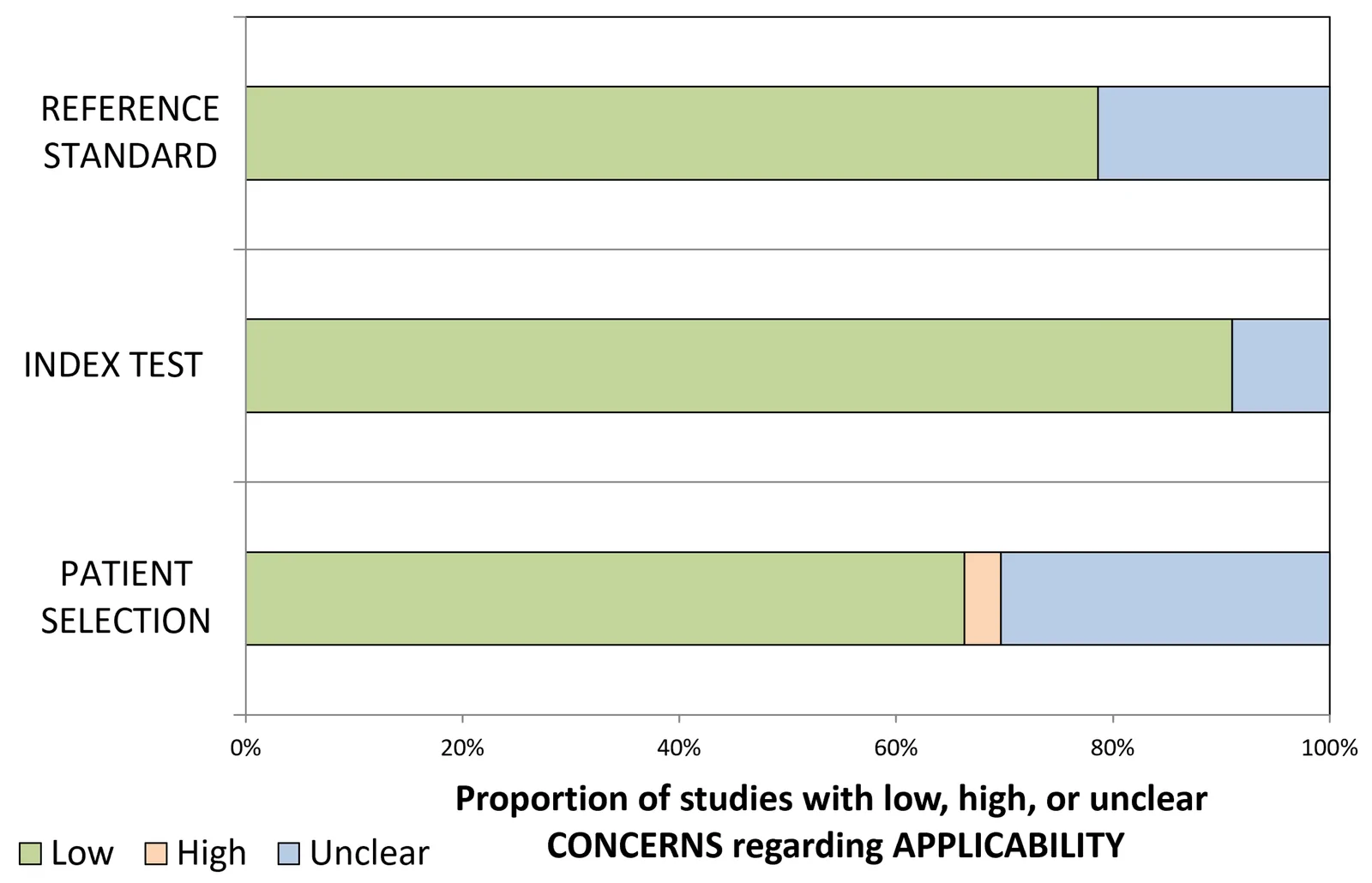
Applicability assessment of the included studies according to QUADAS-2.

The risk of bias and applicability of each study were assessed using the Quality Assessment of Diagnostic Accuracy Studies (QUADAS-2) tool [9], and its results are shown in Figure 2 and Figure 3. Two high-risk biases were reported in all the included studies, and 29 studies involved unclear information on patient selection, 17 studies involved that on reference standards, 23 studies involved that on flow and timing, and 12 studies involved that on index tests. Three studies were reported for high-risk applicability in all the included studies, 29 studies involved unclear information on patient selection, 18 studies involved that on reference standards, and 8 studies involved that on index tests.

### 3.3. Accuracy for ICH Detection

In total, eighty-two studies tested the performance of AI algorithms in ICH detection in NCCT scans. From the above studies, fifty-one provided the required information to calculate their pooled diagnostic accuracy for hemorrhage detection, which is presented in Table 2.

### 3.4. Diagnostic Accuracy in Distinguishing ICH Subtypes

Thirty-seven studies assessed AI algorithms for detecting subarachnoid hemorrhage (SAH), 31 for intraparenchymal hemorrhage (IPH) and intraventricular hemorrhage (IVH), and 30 for subdural hemorrhage (SDH) and epidural hemorrhage (EDH). Not all studies presented adequate information for the performance metrics to be included in the meta-analysis.

**Figure 4:**
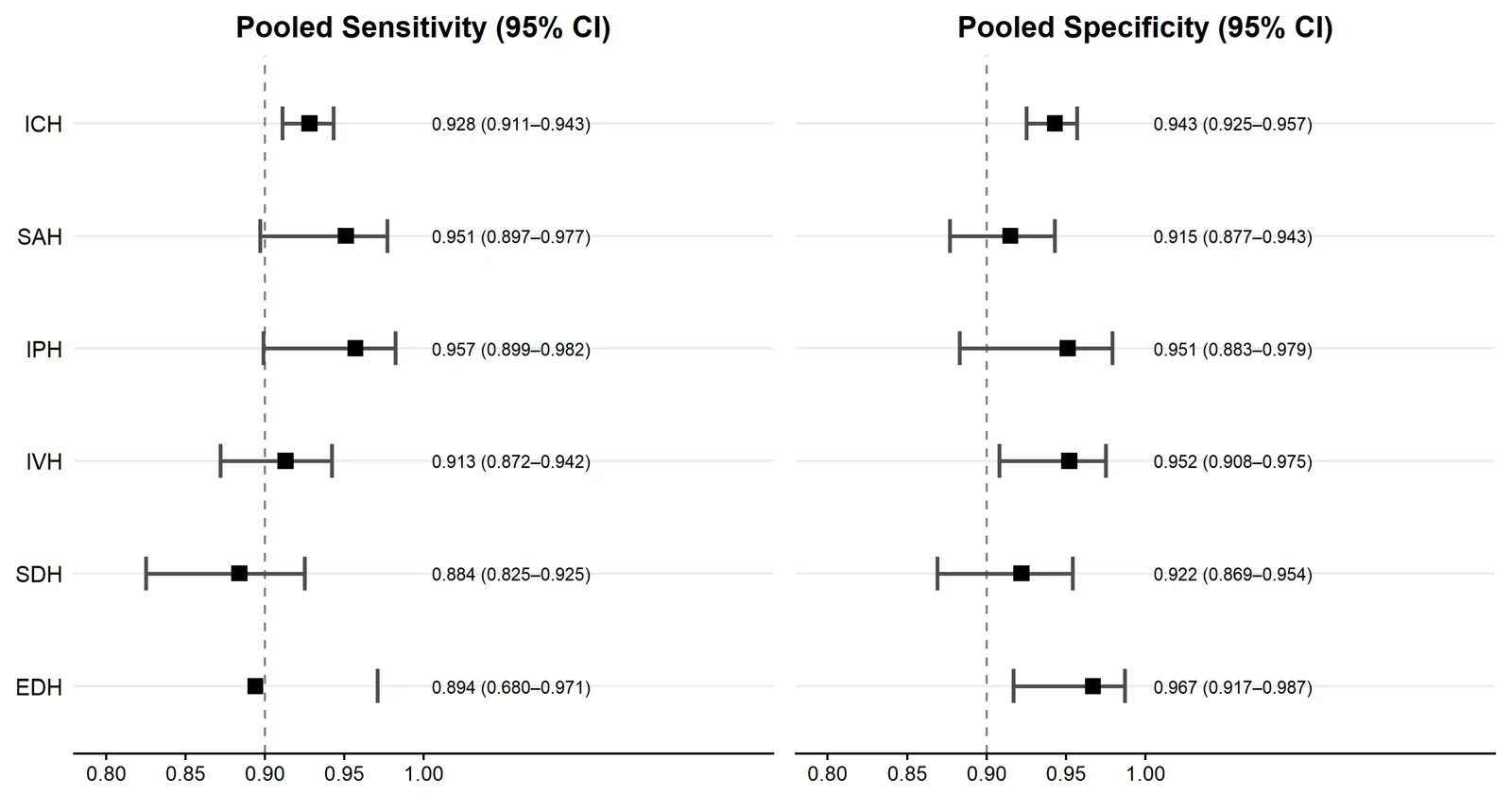
Summary of pooled sensitivity and specificity of algorithms tested with a combination of both datasets for the detection of ICH and its subtypes.

The pooled sensitivity was highest for SAH detection at 95.1% (95% CI: 89.7–97.7), followed by IPH (95.7%, 95% CI: 89.9–98.2), IVH (91.3%, 95% CI: 87.2–94.2), EDH (89.4%, 95% CI: 68.0–97.1), and SDH (88.4%, 95% CI: 82.5–92.5). For specificity, EDH detection achieved the highest pooled value (96.7%, 95% CI: 91.7–98.7), with other subtypes showing specificity values ranging from 90.9% to 95.2%. The pooled diagnostic accuracy metrics for all subtypes are detailed in Table 2, while a summary of these findings is illustrated in Figure 4.

### 3.5. Private vs. Public Test Datasets

Forty-five studies utilized private datasets, while 39 used public datasets. Private datasets were predominantly derived from single-center studies (*n* = 38), whereas most public datasets (*n* = 36) relied on the RSNA ICH Detection Challenge dataset.

For ICH detection, the pooled sensitivity for algorithms tested on private datasets was 92.5% (95% CI: 89.7–94.6), with a specificity of 95.3% (95% CI: 93.3–96.7). Algorithms tested on public datasets achieved a comparable sensitivity of 93.2% (95% CI: 90.9–94.9) and specificity of 92.6% (95% CI: 88.9–95.2).

**Figure 5:**
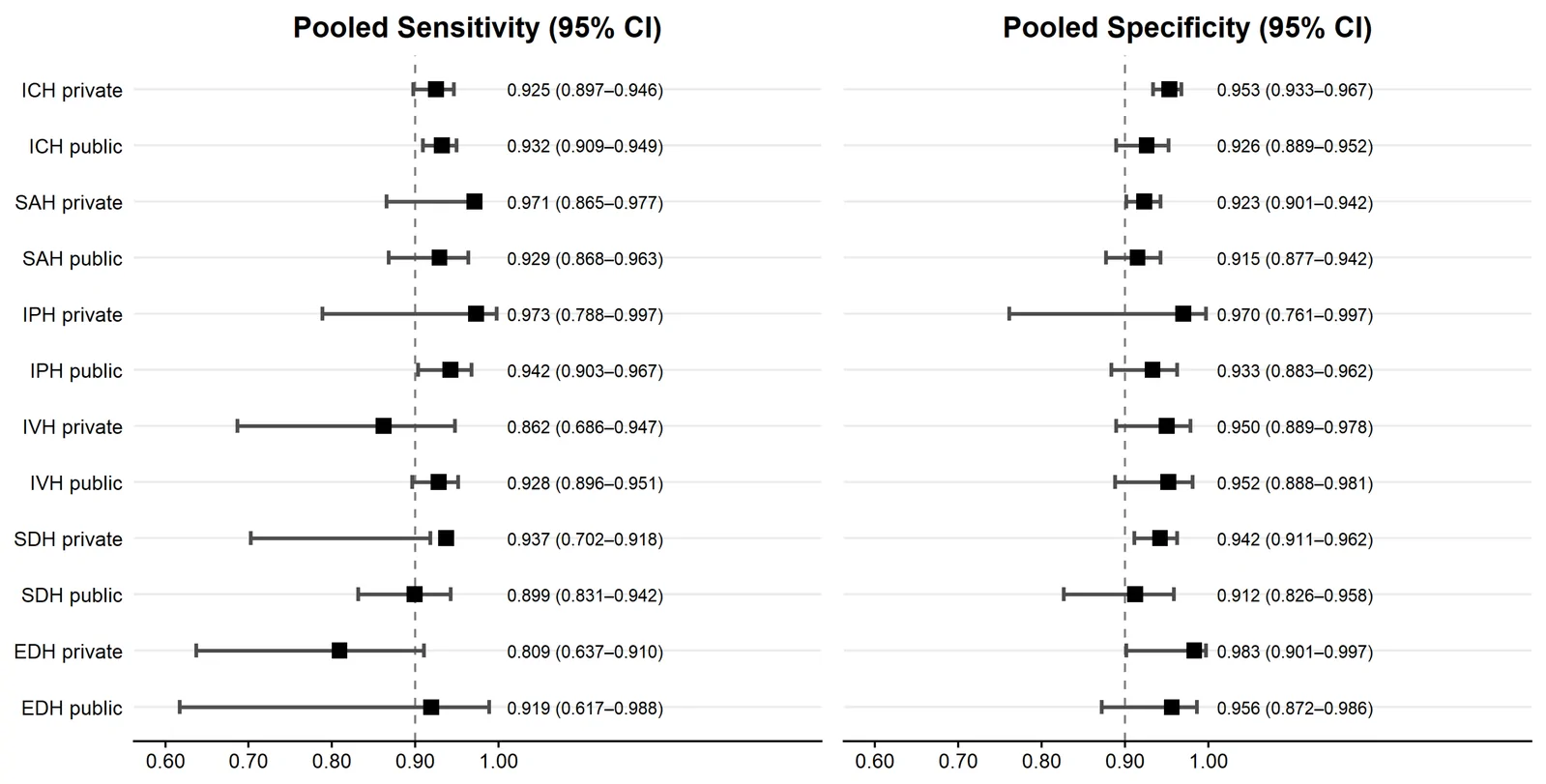
Summary of pooled sensitivity and specificity of algorithms tested using private and public datasets for the detection of ICH and its subtypes.

The pooled diagnostic accuracy metrics for private and public datasets across ICH subtypes are summarized in Figure 5.

Subgroup analysis showed variability in performance for specific subtypes. For SAH, private datasets yielded a higher sensitivity (97.1%, 95% CI: 86.5–99.4) compared to public datasets (92.9%, 95% CI: 86.8–96.3), while specificity was similar. For IPH, private datasets showed slightly better sensitivity (97.3%, 95% CI: 78.8–99.7) and specificity (97.0%, 95% CI: 76.1–99.7) compared to public datasets (94.2%, 95% CI: 90.3–96.7; 93.3%, 95% CI: 88.3–96.2). In contrast, IVH detection algorithms performed better on public datasets, with higher pooled sensitivity (92.8%, 95% CI: 89.6–95.1) and comparable specificity (95.2%, 95% CI: 88.8–98.1).

### 3.6. FDA vs. Non-FDA Marked AI Algorithms

**Figure 6:**
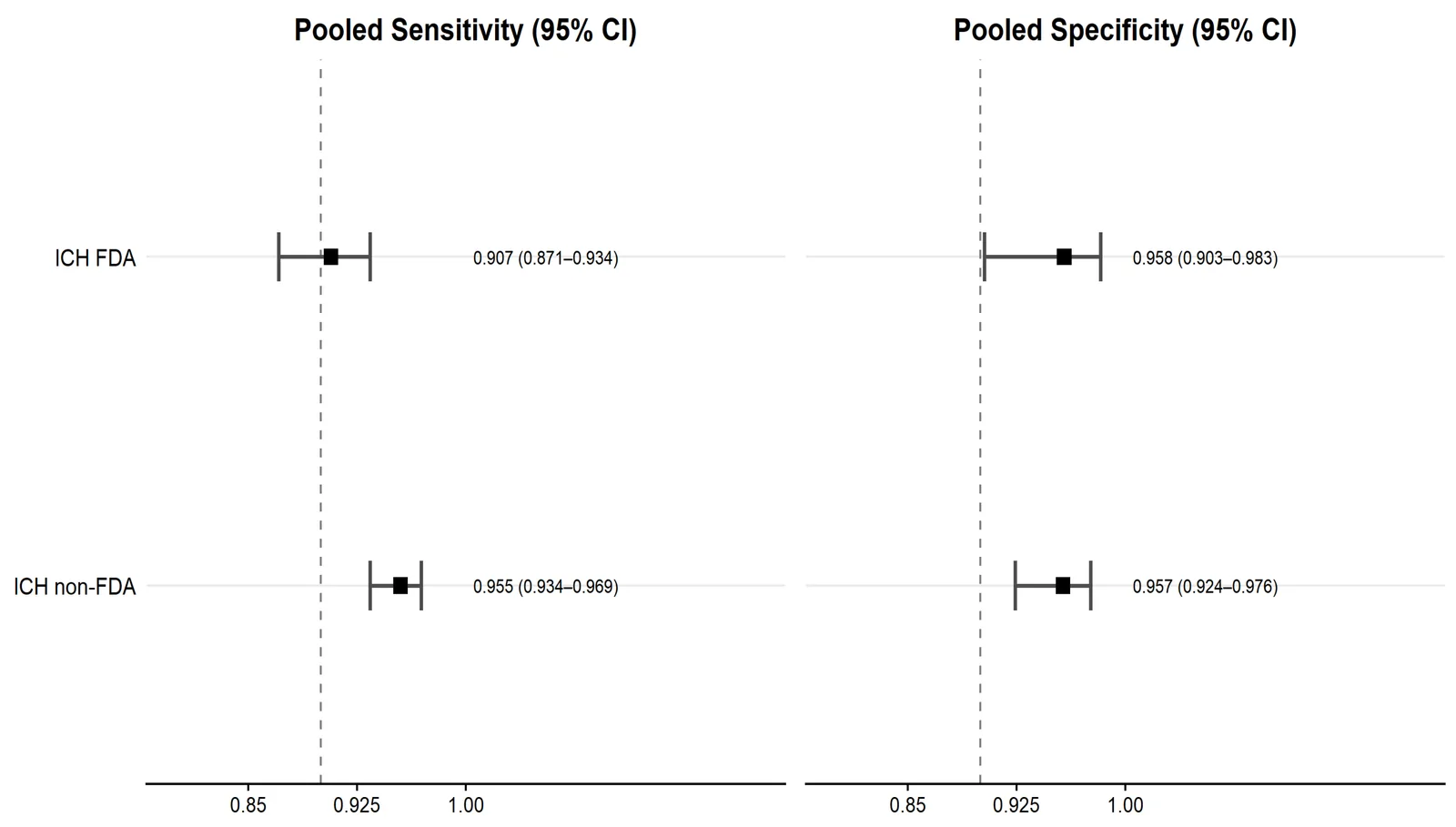
Summary of pooled sensitivity and specificity of algorithms for the detection of ICH and its subtypes.

Fifteen studies that examined the ability of commercially approved algorithms to detect ICH were included, with pooled sensitivity and specificity presented in Figure 6 [10,11,12,13,14,15,16,17,18,19,20,21,22,23,24,25]. The performance of FDA-approved methods was comparable to non-FDA algorithms. FDA-approved models demonstrated a pooled sensitivity of 90.7% (95% CI: 87.1–93.4) and specificity of 95.8% (95% CI: 90.3–98.3), while non-FDA-approved models achieved a slightly higher pooled sensitivity of 95.5% (95% CI: 93.4–96.9) and specificity of 95.7% (95% CI: 92.4–97.6). All FDA-approved AI tools included in this review were tested on private datasets, with four studies [13,15,19,22] using NCCT images from multiple medical centers.

**Table 1:** Characteristics of detection algorithms.

Characteristics of Artificial Intelligence Algorithms for ICH Detection								
Study	DL or ML	ICH Type	Architecture	Reference Standard	Input	# of Inputs per Dataset		
						Training	Validation	Test
Li 2012 retrospective [26]	ML	SAH	SVM	Expert consensus	2D NCCT	60 patients	NR	69 patients
Li 2012 retrospective [27]	ML	SAH	SVM	Expert consensus	2D NCCT	60 patients	NR	69 patients
Prevedello 2017 [28], retrospective and prospective	ML	ICH	NR	Expert consensus	2D NCCT	222	NR	130
Arbabshirani 2018 [29], retrospective	DL	ICH	CNN	Expert consensus	3D NCCT	46,583	NR	9846
Chang 2018 [30], retrospective and prospective	DL	ICH and subtypes	Fast-Recurrent CNN	Clinical reports and one senior radiologist	3D NCCT	10,159	NR	682
Chilamkurthy 2018 [31], retrospective	DL	ICH and subtypes	ResNet-18	Clinical reports and expert consensus	2D NCCT	290,055	NR	21,586
Dawud 2019 [32], retrospective	DL + ML	ICH	AlexNet + SVM	Expert consensus	2D NCCT + 4096 DL features	8855	NR	3790
Cho 2019 [33], retrospective	DL	ICH and subtypes	Cascade LeNet + FCN-VGG16	Expert consensus	2D NCCT	5702	NR	NR
Ye 2019 [34], retrospective	DL	ICH and subtypes	CNN + RNN	Expert consensus	3D NCCT	2836	NR	299
Kuo 2019 [35], retrospective	DL	ICH	ResNet18 + Patch-FCN	Expert consensus	2D NCCT	4396	NR	200
Ginat 2020 [10], prospective	DL	ICH	NR	Senior radiologist	2D NCCT	NR	NR	2011
Karki 2020 [36], retrospective	DL	ICH and subtypes	Deep CNN + Window Estimator	Expert consensus	2D NCCT	30,000	7500	7555
Danilov 2020 [37], retrospective	DL	ICH and subtypes	SE-ResNeXt–50	Expert consensus	2D NCCT	NR	NR	785,807
Ko 2020 [38], retrospective	DL	ICH and subtypes	Xception + LSTM	Expert consensus	2D NCCT	NR	NR	727,392
Burduja 2020 [39], retrospective	DL	ICH and subtypes	ResNetX101 + Recurrent NN	Clinical reports and expert consensus	3D NCCT	728,513, 21,000 patients	24,290, 744 patients	145,522, 3528 patients
Lee 2020 [40], retrospective	DL	ICH and subtypes	Shallow ANN	Expert consensus	2D NCCT	332	NR	84
Wang 2021 [41], retrospective	DL	ICH and subtypes	DenseNet + Recurrent NN	Clinical reports	2D NCCT	19,530 patients	NR	2780 patients
Heit 2021 [13], retrospective	DL	ICH	Deep CNN RAPID	Expert consensus	3D NCCT	805	NR	308
Kumaravel 2021 [42], retrospective	DL + ML	ICH	AlexNet + SVM	Clinical reports	2D NCCT	17,156	NR	4288
Li 2021 [43], retrospective	DL	ICH	U-Net + GANs	Expert consensus	2D NCCT	159 patients	NR	NR
Rao 2021 [15], retrospective	DL	ICH	NR	Clinical reports	2D NCCT	6565 patients	NR	5585 patients
Watanabe 2021 [44], retrospective	DL + ML	ICH	U-Net + Random Forest	Senior radiologist	2D NCCT + DL features	444 patients	NR	40 patients
Rava 2021 [14], retrospective	DL	ICH and subtypes	NR	Senior radiologist	2D NCCT	10,000 patients	814 patients	302 patients
Voter 2021 [11], retrospective	DL	ICH	NR	Senior radiologist	2D NCCT	10,000	NR	3605
Solorio-Ramirez 2021 [45], retrospective	ML	IVH	k-NN, RF, SVM, Hierarchical Minimalist ML	Expert consensus	2D NCCT + texture features	252 patients	NR	NR
Salehinejad 2021 [46], retrospective	DL + ML	ICH and subtypes	SE-ResNeXt 50 + Gradient Boost	Clinical reports	2D NCCT + DL features	752,803	NR	5965
Nishi 2021 [47], retrospective	DL	SAH	U-Net	Clinical reports	2D and 3D NCCT	708 patients	49 patients	785 patients
Goyal 2022 [48], retrospective	ML	ICH	RF, DT, k-NN, SVM	Senior radiologist	2D NCCT	160 patients	NR	40 patients
Hopkins 2022 [49], retrospective	DL	ICH	DenseNet-121	Expert consensus	2D NCCT	752,807	NR	726
Alfaer 2022 [50], retrospective	DL + ML	ICH and subtypes	EfficientNet + fuzzy SVM	Clinical reports	2D NCCT + DL extracted	NR	NR	341
Algaweed 2022 [51], retrospective	ML	SAH	NCFP-growth algorithm	Clinical reports	2D NCCT + features	1000	NR	NR
Guo 2022 [52], retrospective–prospective	DL	ICH	RoLo CNN	Clinical reports and a senior radiologist	2D NCCT	104,594	NR	491
Neethi 2022 [53], retrospective	DL	ICH	Pre-Process Tomo + CNN (F&M block)	Expert consensus	3D NCCT	147 patients	39 patients	48 patients
Dyer 2022 [54], retrospective	DL	ICH	EfficientNet	Expert consensus	2D NCCT	2059	NR	390
Alis 2022 [55], retrospective	DL	ICH and subtypes	Inception–ResNetV2 + RNN	Expert consensus	2D NCCT	49,968	5211	511
Eldaya 2022 [16], retrospective	DL	ICH	NR	Expert consensus	2D NCCT	NR	NR	307 patients
Ertugrul 2022 [56], retrospective	DL	ICH and subtypes	YoLo2	Expert consensus	2D NCCT	14,381	NR	1598
Chen 2022 [57], retrospective	DL	ICH	ResNet50	Expert consensus	2D NCCT	17,339	4954	2476
Schmitt 2022 [17], retrospective	DL	ICH	Brainomix U-Net	Senior neuro- radiologist	2D NCCT	NR	NR	160 patients
Mair 2022 [19], retrospective	DL	ICH	e-ASPECTS NR	Expert consensus	2D NCCT	NR	NR	4100
Kau 2022 [12], retrospective	DL	ICH	CNN	Expert consensus	2D NCCT	NR	NR	4100
Gibson 2022 [58], retrospective	DL	ICH and subtypes	Dense U-Net + Classifier	Expert consensus	2D NCCT	25,946	400	16,764
Lopez-Perez 2022 [59], retrospective	DL	ICH	Dense–U-Net & Axial Classifier	Clinical reports and expert consensus	2D NCCT	1000 patients	NR	640 patients
Altuve 2022 [60], retrospective	DL	ICH	ResNet-18	Senior radiologist	2D NCCT	200 patients	NR	NR
Kyung 2022 [61], retrospective	DL	ICH	SmartNet	Clinical reports and expert consensus	2D NCCT	50,763	2248	2736
Yalcin 2023 [62], retrospective	DL	ICH	Deep CNN + U-Net	Clinical reports	2D NCCT	7099	NR	1774
Warman 2022 [18], retrospective	DL	ICH and subtypes	CNN + LSTM	Expert consensus	2D NCCT	NR	NR	526
Ozaltin 2022 [63], retrospective	DL + ML	ICH	OzNet (CNN) + Feature Extraction + Naïve Bayes	Clinical reports	2D NCCT + DL features	1900	NR	NR
Gudadhe 2022 [64], retrospective	ML	ICH	DR, RF, SVM, k-NN	Clinical reports	Texture-based features	1426	NR	125
Rajagopal 2023 [65], retrospective	DL	ICH and subtypes	CNN + LSTM	Clinical reports	2D NCCT	72,516	7512	7512
Yun 2023 [66], retrospective	DL	ICH	CNN + LSTM	Clinical reports and expert consensus	2D NCCT	3010 patients	NR	49,841 patients
Cheung 2023 [67], retrospective	DL	ICH and subtypes	ResNeXt-101	Clinical reports and expert consensus	2D NCCT	14,488 patients	2557 patients	1894 patients
Kothala 2023 [68], retrospective	DL	ICH and subtypes	YoLov5x	Clinical reports and expert consensus	2D NCCT	491 patients	NR	75 patients
Cortes-Ferre 2023 [69], retrospective	DL	ICH	EfficientNet + ResNet	Clinical reports and expert consensus	2D NCCT	195,050	10,802	10,014
Asif 2023 [70], retrospective	DL + ML	ICH and subtypes	ResNet101 + InceptionV4 + Light GBM	Clinical reports and expert consensus	2D NCCT + DL extracted features	9334 patients	NR	491 patients
Thanellas 2023 [71], retrospective, prospective	DL	SAH	U-Net	Clinical reports and expert consensus	2D NCCT	110 patients	NR	279 patients
Abrigo 2023 [72], retrospective	DL	ICH and subtypes	VGG16	Clinical reports and expert consensus	2D NCCT	639,886	NR	112,921
Chen 2023 [73], retrospective	ML + DL	ICH	Weight update method + CNN	Clinical reports	2D NCCT	468	66	66
Neves 2023 [20], retrospective	DL	ICH and subtypes	NR	Expert consensus	2D NCCT	NR	NR	510
Yeo 2023 [74], retrospective	DL	ICH and subtypes	CNN + ResNeXt + Recurrent NN	Clinical reports and expert consensus	2D NCCT	602,242	150,560	193,317
Kang 2023 [75], retrospective	DL	ICH and subtypes	MobileNetV2, VGG19, Ensemble Weighted	Clinical reports and expert consensus	2D NCCT	80,906	NR	1091
Sengupta 2023 [76], retrospective	DL	ICH and subtypes	Bi-LSTM + CNN	Clinical reports	2D NCCT	25,272 patients	NR	NR
Wang 2023 [77], retrospective	DL	ICH	ResNeXt	Expert Consensus	2D and 3D NCCT	20,000	NR	527
Umapathy 2023 [78], retrospective	DL	ICH and subtypes	ResNeXt-2D + LSTM	Clinical reports	2D	25,272 patients	NR	491 patients
Chen 2024 [79], retrospective	DL	ICH and subtypes	DCNN + depthwise separable structure	Clinical reports	2D	18,938 patients	NR	566 patients
He 2024 [80], retrospective	DL	ICH and subtypes	VGG-16	Clinical reports	2D	750,000	NR	7500 patients
Lin 2024 [81], retrospective	DL	ICH	PatchFCN	Clinical reports and expert consensus	2D	25,000	NR	491 patients
Teneggi [82], retrospective	DL	ICH	MIL + ResNet	Clinical reports	2D	21,736 patients	NR	566 patients
Priya 2024 [83], retrospective	DL	ICH	DenseNet121-LSTM	clinical reports	2D	1600	NR	600
Boujelb 2024 [84], retrospective	ML	ICH	XGBoost	clinical reports	2D	266	NR	85
DelGaizo 2024 [12], retrospective	DL	ICH	NR	clinical reports	2D	NR	NR	58,321
Gamble 2024 [85], retrospective	DL	ICH and subtypes	YOLO	clinical reports	2D	491	NR	3650
Gencturk 2024 [86], retrospective	DL	ICH and SDH	EfficieNet	clinical reports	2D	491	NR	191
Kaur 2024 [87], retrospective	DL	ICH and subtypes	YOLO	clinical reports	2D	6000	NR	NR
Nagaraju 2024 [88], retrospective	DL	ICH and subtypes	Ensemble Learning	clinical reports	2D	2000	NR	500
Pettet 2024 [15], retrospective	DL	ICH	NR	neuroradiologists	2D	NR	NR	1315
Savage 2024 [21], retrospective	DL	ICH	NR	clinical reports	2D	NR	NR	3716
Sengupta 2024 [89], retrospective	ML	SAH	DWSCSO-PRSCNN	clinical reports	2D	1120	NR	280
Thalhammer 2024 [90], retrospective	DL	ICH and subtypes	U-Net	clinical reports	2D	2400 patients	NR	600 patients
Chaudhary 2025 [91], retrospective	DL	ICH	Ensemble Learning	clinical reports	2D	140,000	NR	35,000
Fan 2025 [92], retrospective	DL	ICH	DTViT Encoder	clinical reports	2D	22,445	NR	1266
Hassan 2025 [93], retrospective	DL	ICH and subtypes	U-Net	clinical reports	2D	4797	NR	1199
Khoruzhaya 2025 [94], retrospective	DL	ICH	NR	clinical reports	2D	NR	NR	1200
Kim 2025 [95], retrospective	DL	ICH	NR	clinical reports	2D	NR	NR	415
Liu 2025 [96], retrospective	DL	ICH and subtypes	YOLOv8	clinical reports	2D	8867	NR	1191
Mahmood 2025 [97], retrospective	DL	ICH and subtypes	NR	clinical reports	2D	NR	NR	NR
Nada 2025 [22], retrospective	DL	ICH and subtypes	NR	clinical reports	2D	NR	NR	5600
Villringer 2025 [23], retrospective	DL	ICH	NR	clinical reports	2D	NR	NR	255 patients
Wang 2025 [98], retrospective	DL	ICH	U-Net	clinical reports	2D	1815	NR	491

**Table 2:** Pooled sensitivity and specificity values for all subgroups.

	Pooled Sensitivity	(95% CI)	(95% PI)	I^2^	τ^2^	Pooled Specificity	(95% CI)	(95% PI)	I^2^	τ^2^
ICH	0.928	(0.911–0.943)	(0.744–0.983)	100.00%	1.4152	0.943	(0.931–0.969)	(0.698–0.992)	100.00%	2.1482
ICH private dataset	0.925	(0.897–0.946)	(0.676–0.987)	100.00%	1.7623	0.953	(0.929–0.972)	(0.747–0.993)	100.00%	1.7926
ICH public dataset	0.932	(0.909–0.949)	(0.791–0.980)	100.00%	0.9964	0.926	(0.901–0.975)	(0.614–0.990)	100.00%	2.7558
SAH	0.951	(0.897–0.977)	(0.589–0.989)	100.00%	3.1641	0.915	(0.877–0.943)	(0.611–0.985)	100.00%	0.8751
SAH private dataset	0.971	(0.865–0.994)	(0.359–0.996)	100.00%	5.6647	0.923	(0.900–0.942)	(0.787–0.976)	100.00%	0.1748
SAH public dataset	0.929	(0.869–0.963)	(0.572–0.990)	100.00%	1.3278	0.909	(0.831–0.953)	(0.412–0.992)	100.00%	1.4365
IPH	0.957	(0.900–0.982)	(0.745–0.984)	100.00%	3.3843	0.950	(0.883–0.980)	(0.639–0.988)	100.00%	3.5982
IPH private dataset	0.973	(0.788–0.997)	(0.782–0.961)	100.00%	8.1513	0.970	(0.761–0.997)	(0.499–0.993)	100.00%	8.4894
IPH public dataset	0.942	(0.903–0.967)	(0.675–0.992)	100.00%	0.8466	0.933	(0.883–0.962)	(0.567–0.992)	100.00%	0.9718
IVH	0.913	(0.872–0.942)	(0.644–0.977)	100.00%	0.678	0.952	(0.908–0.975)	(0.521–0.997)	100.00%	1.7253
IVH private dataset	0.862	(0.686–0.947)	(0.037–0.999)	100.00%	1.1457	0.950	(0.889–0.978)	(0.298–0.999)	100.00%	0.7681
IVH public dataset	0.928	(0.896–0.951)	(0.828–0.958)	100.00%	0.4283	0.952	(0.888–0.981)	(0.370–0.998)	100.00%	2.2351
SDH	0.884	(0.825–0.925)	(0.585–0.967)	100.00%	0.846	0.922	(0.869–0.954)	(0.524–0.991)	100.00%	1.2042
SDH private dataset	0.837	(0.702–0.918)	(0.148–0.994)	100.00%	0.6302	0.942	(0.911–0.962)	(0.671–0.992)	100.00%	0.214
SDH public dataset	0.899	(0.831–0.942)	(0.551–0.976)	100.00%	0.9159	0.912	(0.826–0.958)	(0.359–0.994)	100.00%	1.6063
EDH	0.894	(0.680–0.971)	(0.183–0.984)	100.00%	6.4005	0.967	(0.917–0.987)	(0.452–0.998)	100.00%	3.2319
EDH private dataset	0.809	(0.637–0.910)	(0.248–0.978)	99.90%	0.7993	0.983	(0.901–0.997)	(0.934–0.970)	100.00%	3.6585
EDH public dataset	0.919	(0.617–0.988)	(0.086–0.993)	100.00%	8.9591	0.956	(0.872–0.986)	(0.239–0.999)	100.00%	3.1274
ICH FDA	0.907	(0.871–0.934)	(0.658–0.978)	100.00%	0.521	0.958	(0.903–0.983)	(0.843–0.983)	100.00%	3.18
ICH non-FDA	0.955	(0.934–0.969)	(0.758–0.989)	100.00%	1.398	0.957	(0.924–0.976)	(0.545–0.996)	100.00%	3.118

## 4. Discussion

In this systematic review, we demonstrated that AI methods used for detection of ICH and its subtypes achieve high diagnostic accuracy. The pooled sensitivity and specificity for ICH detection were approximately 95%, indicating that these AI algorithms perform comparably to experienced clinicians in identifying ICH on non-contrast CT (NCCT) scans.

One of the key findings is the consistency in diagnostic performance across private and public datasets. The near-identical pooled sensitivity and specificity values suggest that AI algorithms, especially those trained on diverse datasets, exhibit robust generalizability. However, slight differences were observed in the detection of specific ICH subtypes. For instance, algorithms trained on private datasets performed better for SAH and IPH, while those tested on public datasets excelled in detecting IVH and SDH. These variations may reflect the challenges associated with applying AI models to heterogeneous imaging protocols and patient populations.

AI algorithms tested on private datasets generally outperformed those tested on public datasets for specific ICH subtypes such as subarachnoid hemorrhage (SAH) and intraparenchymal hemorrhage (IPH). This may reflect the challenges AI models face when applied to diverse public datasets that may have variations in imaging protocols, scanner types, and patient populations. However, the relatively small differences between private and public performance metrics suggest that AI models are still robust in data similar to real-world variability, though further improvements in training with heterogeneous data could further enhance their generalizability.

Subgroup analysis of FDA-approved and non-FDA-approved algorithms revealed slightly higher sensitivity for non-FDA-approved models. However, both groups achieved similarly high specificity. This suggests that non-FDA-approved algorithms, often developed in academic settings with more experimental flexibility, may show incremental improvements in performance. Nonetheless, FDA-approved models, which have undergone extensive regulatory validation, can be highly reliable and clinically applicable, especially in structured environments such as hospitals, although more research is suggested on their performance on datasets similar to real-world cohorts.

This study also highlights the importance of ensuring that AI models used for clinical decision support are tested across a range of settings to verify their reliability. The integration of AI tools into clinical workflows may significantly reduce diagnostic delays, particularly in emergency settings or resource-limited regions where experienced radiologists may not be readily available. AI tools achieving sensitivity and specificity values similar to clinicians are the first step, out of many, for AI tool incorporation in patient treatment pathways. Nevertheless, additional studies are required to investigate the real impact of AI tools in a clinical environment since high AI performance metrics may not be translated to better patient care in all hospital settings.

Moreover, AI systems have the potential to function as standalone diagnostic tools, although more steps are needed towards the explainability and the reasoning behind those algorithms, as well as the course of action in cases of false positives and false negatives. The more transparent the process behind the decision making of an AI tool is, the more reliable and acceptable the AI system is for healthcare professionals and patients.

Despite the encouraging results, several limitations should be noted. The included studies exhibited significant variability in AI architectures, imaging protocols, and dataset characteristics, which may introduce variability in the results. The use of different reference standards that vary both in the type (clinical reports, expert consensus) and the number and seniority of participating clinicians may not allow us to draw concrete comparisons of the performance metrics of the AI tools. Furthermore, the retrospective nature of most studies limits the ability to assess real-time clinical applicability. Many studies also lacked detailed descriptions of the patient cohort composing the training and test datasets, which hinders a thorough evaluation of model generalizability. The large heterogeneity and between-study variance in the examined AI architectures did not allow for a more direct comparison of diagnostic accuracy between ML and DL methods, since their high values indicated several sources of variability such as the data trained and tested on, both with respect to their size and their quality. Furthermore, the reported performance by the included studies does not indicate full characterization of the AI tools’ failure mode, since high diagnostic performance may coexist with poorly calibrated prediction outcomes in particular clinical cases. Additionally, the presentation of the best performing model within each study may introduce optimistic bias, as the reported accuracy could partially reflect favorable variation in the testing data.

Beyond diagnostic accuracy, the clinical value of these tools will depend on how they are embedded in acute care pathways. In the emergency setting, an AI tool that reliably flags intracranial hemorrhage on non-contrast CT could prioritize positive scans within the radiology worklist, shorten the time to expert review, and prompt earlier neurosurgical consultation. Realizing these benefits will require prospective evaluation, since most current studies are retrospective. Future work should therefore assess not only standalone diagnostic accuracy but also how such tools interact with clinicians and whether their integration improved patient-centered outcomes such as time to diagnosis, time to intervention, and functional outcome.

## 5. Conclusions

The results of this systematic review and meta-analysis demonstrate that AI algorithms for ICH detection achieve high diagnostic accuracy and show promising generalizability across both private and public datasets. FDA-approved and non-FDA-approved models showed comparable performance, reinforcing their reliability and clinical applicability. AI methods hold substantial potential for integration into clinical workflows, particularly in settings with limited radiological expertise. Future research should prioritize prospective trials, evaluate performance in diverse and heterogeneous datasets, and explore the interaction between AI systems and clinicians to optimize their role in improving patient care.

## Data Availability

No new data were created or analyzed in this study. Data sharing is not applicable to this article.

## Supplementary Materials

The following supporting information can be downloaded at https://www.mdpi.com/article/10.3390/diagnostics16213516/s1, PRISMA Checklist. Table S1: Characteristics of ICH studies tested on private datasets. Table S2: Characteristics of ICH studies tested on public datasets. Table S3: Characteristics of SAH studies tested on private datasets. Table S4: Characteristics of SAH studies tested on public datasets. Table S5: Characteristics of IPH studies tested on private datasets. Table S6: Characteristics of IPH studies tested on public datasets. Table S7: Characteristics of IVH studies tested on private datasets. Table S8: Characteristics of IVH studies tested on public datasets. Table S9: Characteristics of SDH studies tested on private datasets. Table S10: Characteristics of SDH studies tested on public datasets. Table S11: Characteristics of EDH studies tested on private datasets. Table S12: Characteristics of EDH studies tested on public datasets. Table S13: Characteristics of FDA-approved ICH studies. Table S14: Characteristics of Non-FDA-approved ICH studies. Figure S1. Sensitivity of ICH studies tested on both private and public datasets. Figure S2. Specificity of ICH studies tested on both private and public datasets. Figure S3. Funnel plot for ICH studies tested on both private and public datasets. Figure S4. Sensitivity of ICH studies tested on private datasets. Figure S5. Specificity of ICH studies tested on private datasets. Figure S6. Sensitivity of ICH studies tested on public datasets. Figure S7. Specificity of ICH studies tested on public datasets. Figure S8. Sensitivity of SAH studies tested on both private and public datasets. Figure S9. Specificity of SAH studies tested on both private and public datasets. Figure S10. Sensitivity of SAH studies tested on private datasets. Figure S11. Specificity of SAH studies tested on private datasets. Figure S12. Sensitivity of SAH studies tested on public datasets. Figure S13. Specificity of SAH studies tested on public datasets. Figure S14. Sensitivity of IPH studies tested on both private and public datasets. Figure S15. Specificity of IPH studies tested on both private and public datasets. Figure S16. Sensitivity of IPH studies tested on private datasets. Figure S17. Specificity of IPH studies tested on private datasets. Figure S18. Sensitivity of IPH studies tested on public datasets. Figure S19. Specificity of IPH studies tested on public datasets. Figure S20. Sensitivity of IVH studies tested on both private and public datasets. Figure S21. Specificity of IVH studies tested on both private and public datasets. Figure S22. Sensitivity of IVH studies tested on private datasets. Figure S23. Specificity of IVH studies tested on private datasets. Figure S24. Sensitivity of IVH studies tested on public datasets. Figure S25. Specificity of IVH studies tested on public datasets. Figure S26. Sensitivity of SDH studies tested on both private and public datasets. Figure S27. Specificity of SDH studies tested on both private and public datasets. Figure S28. Sensitivity of SDH studies tested on private datasets. Figure S29. Specificity of SDH studies tested on private datasets. Figure S30. Sensitivity of SDH studies tested on public datasets. Figure S31. Specificity of SDH studies tested on public datasets. Figure S32. Sensitivity of EDH studies tested on both private and public datasets. Figure S33. Specificity of EDH studies tested on both private and public datasets. Figure S34. Sensitivity of EDH studies tested on private datasets. Figure S35. Specificity of EDH studies tested on private datasets. Figure S36. Sensitivity of EDH studies tested on public datasets. Figure S37. Specificity of EDH studies tested on public datasets. Figure S38. Sensitivity of FDA-approved ICH studies. Figure S39. Specificity of FDA-approved ICH studies. Figure S40. Sensitivity of Non-FDA-approved ICH studies. Figure S41. Specificity of Non-FDA-approved ICH studies.

## Appendix A

### Search Terms

PubMed

(“Intracranial Hemorrhages”[Mesh] OR “Hemorrhagic Stroke”[Mesh] OR stroke[tiab] OR hematoma[tiab] OR hemorrhag*[tiab])

AND

(“Algorithms”[Mesh] OR algorithm*[tiab] OR artificial intelligence[tiab] OR deep learning[tiab] OR machine learning[tiab] OR “Diagnosis, Computer-Assisted”[Mesh] OR computer assisted[tiab] OR computer-aided[tiab])

AND

(“Tomography, X-Ray Computed”[Mesh] OR computed tomograph*[tiab] OR “head ct”[tiab:~3] OR cranial ct[tiab] OR cerebral ct[tiab] OR brain ct[tiab]) NOT (“animals”[MeSH] NOT “humans”[MeSH])

Scopus

(TITLE-ABS ({stroke} OR {hematoma} OR {hemorrhage} OR {haemorrhage} OR {haematoma}) OR AUTHKEY ({stroke} OR {hematoma} OR {hemorrhage} OR {haemorrhage} OR {haematoma}))

AND

(TITLE-ABS ({algorithm} OR {artificial intelligence} OR {deep learning} OR {machine learning} OR {computer assisted} OR {computer-aided} OR {computer-assisted} OR {computer aided}) OR AUTHKEY ({algorithm} OR {Algoritms} OR {artificial intelligence} OR {deep learning} OR {machine learning} OR {computer assisted} OR {computer-aided} OR {computer-assisted} OR {computer aided}))

AND

(TITLE-ABS ({computed tomography} OR {computerized tomography} OR {head ct} OR {cranial ct} OR {cerebral ct} OR {brain ct}) OR AUTHKEY ({computed tomography} OR {computerized tomography} OR {head ct} OR {cranial ct} OR {cerebral ct} OR {brain ct}))

AND (EXCLUDE (DOCTYPE, “cp”))

Embase

(‘brain hemorrhage’/exp OR (stroke OR hematoma OR hemorrhag*):ab,ti,kw)

AND

(‘algorithm’/exp OR ‘computer assisted diagnosis’/de OR (algorithm* OR ‘artificial intelligence’ OR ‘deep learning’ OR ‘machine learning’ OR ‘computer assisted’ OR ‘computer-aided’):ab,ti,kw)

AND

(‘x-ray computed tomography’/exp OR (‘computed tomograph*’ OR (head NEAR/3 ct) OR ‘cranial ct’ OR ‘cerebral ct’ OR ‘brain ct’):ab,ti,kw) NOT (‘animal’/exp NOT ‘human’/exp) NOT ‘conference abstract’/it
