# Supplementary material for: Diagnostic Accuracy of Machine and Deep Learning Methods in Intracranial Hemorrhage Detection: A Systematic Review and Meta-Analysis
Source: Diagnostics (Basel). 2026 Oct 27;16(21):3516. doi: 10.3390/diagnostics16213516 (PMC13762282; doi:10.3390/diagnostics16213516)
Supplement: Supplementary file 1 [file diagnostics-16-03516-s001.zip › diagnostics-4374597-supplementary.pdf]

Systematic Review – Supplementary Material

Diagnostic Accuracy of Machine and Deep Learning Methods in Intracranial Hemorrhage Detection: A Systematic Review and Meta-Analysis

PRISMA CHECKLIST

Section and Topic	Item #	Checklist item	Location where item is reported
**TITLE**			
Title	1	Identify the report as a systematic review.	Page 1
**ABSTRACT**			
Abstract	2	See the PRISMA 2020 for Abstracts checklist.	Page 1
**INTRODUCTION**			
Rationale	3	Describe the rationale for the review in the context of existing knowledge.	Page 2
Objectives	4	Provide an explicit statement of the objective(s) or question(s) the review addresses.	Page 2
**METHODS**			
Eligibility criteria	5	Specify the inclusion and exclusion criteria for the review and how studies were grouped for the syntheses.	Page 3
Information sources	6	Specify all databases, registers, websites, organisations, reference lists and other sources searched or consulted to identify studies. Specify the date when each source was last searched or consulted.	Page 3
Search strategy	7	Present the full search strategies for all databases, registers and websites, including any filters and limits used.	Page 3; Appendix
Selection process	8	Specify the methods used to decide whether a study met the inclusion criteria of the review, including how many reviewers screened each record and each report retrieved, whether they worked independently, and if applicable, details of automation tools used in the process.	Page 3
Data collection process	9	Specify the methods used to collect data from reports, including how many reviewers collected data from each report, whether they worked independently, any processes for obtaining or confirming data from study investigators, and if applicable, details of automation tools used in the process.	Page 3
Data items	10a	List and define all outcomes for which data were sought. Specify whether all results that were compatible with each outcome domain in each study were sought (e.g. for all measures, time points, analyses), and if not, the methods used to decide which results to collect.	Page 3
	10b	List and define all other variables for which data were sought (e.g. participant and intervention characteristics, funding sources). Describe any assumptions made about any missing or unclear information.	Page 3
Study risk of bias assessment	11	Specify the methods used to assess risk of bias in the included studies, including details of the tool(s) used, how many reviewers assessed each study and whether they worked independently, and if applicable, details of automation tools used in the process.	Page 3
Effect measures	12	Specify for each outcome the effect measure(s) (e.g. risk ratio, mean difference) used in the synthesis or presentation of results.	Page 4
Synthesis methods	13a	Describe the processes used to decide which studies were eligible for each synthesis (e.g. tabulating the study intervention characteristics and comparing against the planned groups for each synthesis (item #5)).	Page 3

Section and Topic	Item #	Checklist item	Location where item is reported
	13b	Describe any methods required to prepare the data for presentation or synthesis, such as handling of missing summary statistics, or data conversions.	Page 3
	13c	Describe any methods used to tabulate or visually display results of individual studies and syntheses.	N/A
	13d	Describe any methods used to synthesize results and provide a rationale for the choice(s). If meta-analysis was performed, describe the model(s), method(s) to identify the presence and extent of statistical heterogeneity, and software package(s) used.	N/A
	13e	Describe any methods used to explore possible causes of heterogeneity among study results (e.g. subgroup analysis, meta-regression).	N/A
	13f	Describe any sensitivity analyses conducted to assess robustness of the synthesized results.	N/A
Reporting bias assessment	14	Describe any methods used to assess risk of bias due to missing results in a synthesis (arising from reporting biases).	Page 3
Certainty assessment	15	Describe any methods used to assess certainty (or confidence) in the body of evidence for an outcome.	Page 3
**RESULTS**			
Study selection	16a	Describe the results of the search and selection process, from the number of records identified in the search to the number of studies included in the review, ideally using a flow diagram.	Page 4; Figure 1
	16b	Cite studies that might appear to meet the inclusion criteria, but which were excluded, and explain why they were excluded.	N/A
Study characteristics	17	Cite each included study and present its characteristics.	Table 1
Risk of bias in studies	18	Present assessments of risk of bias for each included study.	Figure 2
Results of individual studies	19	For all outcomes, present, for each study: (a) summary statistics for each group (where appropriate) and (b) an effect estimate and its precision (e.g. confidence/credible interval), ideally using structured tables or plots.	N/A
Results of syntheses	20a	For each synthesis, briefly summarise the characteristics and risk of bias among contributing studies.	Pages 4-5
	20b	Present results of all statistical syntheses conducted. If meta-analysis was done, present for each the summary estimate and its precision (e.g. confidence/credible interval) and measures of statistical heterogeneity. If comparing groups, describe the direction of the effect.	Figures 4-5-6
	20c	Present results of all investigations of possible causes of heterogeneity among study results.	N/A
	20d	Present results of all sensitivity analyses conducted to assess the robustness of the synthesized results.	N/A
Reporting biases	21	Present assessments of risk of bias due to missing results (arising from reporting biases) for each synthesis assessed.	N/A
Certainty of evidence	22	Present assessments of certainty (or confidence) in the body of evidence for each outcome assessed.	N/A
**DISCUSSION**			
Discussion	23a	Provide a general interpretation of the results in the context of other evidence.	Page 5
	23b	Discuss any limitations of the evidence included in the review.	Page 5
	23c	Discuss any limitations of the review processes used.	Page 5
	23d	Discuss implications of the results for practice, policy, and future research.	Page 6

Section and Topic	Item #	Checklist item	Location where item is reported
**OTHER INFORMATION**			
Registration and protocol	24a	Provide registration information for the review, including register name and registration number, or state that the review was not registered.	Not registered
	24b	Indicate where the review protocol can be accessed, or state that a protocol was not prepared.	Not prepared
	24c	Describe and explain any amendments to information provided at registration or in the protocol.	N/A
Support	25	Describe sources of financial or non-financial support for the review, and the role of the funders or sponsors in the review.	Page 7
Competing interests	26	Declare any competing interests of review authors.	N/A
Availability of data, code and other materials	27	Report which of the following are publicly available and where they can be found: template data collection forms; data extracted from included studies; data used for all analyses; analytic code; any other materials used in the review.	All material is available

Tables of each ICH sub-group included in the Meta-Analysis

Table S1: Characteristics of ICH studies tested on private datasets

Study	Events	Total	Sensitivity	Specificity	TP	FP	FN	TN
Prevedello 2017 (1)	50	85	90.00	85.00	45	5	5	30
Chang 2018 (2)	82	682	95.10	98.10	78	11	4	589
Kuo 2019 (3)	25	200	99.99	90.00	25	18	0	158
Ye 2019 (4)	194	299	99.00	99.00	192	1	2	104
Ginat 2020 (5)	373	2011	88.70	94.20	331	95	42	1543
Lee 2020 (6)	50	84	84.00	77.00	42	8	8	26
Karki 2020 (7)	441	541	89.80	98.50	396	2	45	99
Heit 2021 (8)	158	308	95.60	95.30	151	7	7	143
Watanabe 2021 (9)	24	40	91.70	81.20	22	3	2	13
Rava 2021 (10)	200	302	93.00	93.00	186	7	14	95
Voter 2021 (11)	349	3625	92.30	97.70	322	75	27	3201
Alis 2022 (12)	167	547	96.41	95.79	161	16	6	364
Eldaya 2022 (13)	37	307	91.90	84.40	34	42	3	228
Schmitt 2022 (14)	79	160	91.00	89.00	72	9	7	72
Mair 2022 (15)	627	3266	94.00	83.00	588	89	39	2550
Kau 2022 (16)	214	2188	68.20	96.80	146	63	68	1911
Warman 2022 (17)	391	526	94.50	92.10	369	11	22	124
Ozaltin 2022 (18)	285	570	97.54	99.30	278	2	7	283
Wang 2023 (19)	79	527	92.00	96.00	73	18	6	430
Yun 2023 (20)	6442	49841	94.40	98.20	6081	781	361	42618
Neves 2023 (21)	402	510	97.50	99.99	392	0	10	108
Boujelb 2024 (22)	67	85	98.00	77.00	62	5	1	17
Del Gaizo 2024 (23)	5656	58321	75.60	92.10	1192	4464	384	52281

Pettet 2024 (24)	165	1315	85.70	94.30	96	69	16	1134
Savage 2024 (25)	814	3716	87.80	94.30	645	169	90	2812
Fan 2025 (26)	807	1266	99.80	99.60	807	0	1	458
Nada 2025 (27)	1595	5600	89.00	96.00	1420	160	175	3845
Villringer 2025 (28)	38	257	90.00	96.00	29	9	4	215

Table S2: Characteristics of ICH studies tested on public datasets

Study	Events	Total	Sensitivity	Specificity	TP	FP	FN	TN
Chilamkurthy 2018 (29)	205	491	94.63	70.98	194	83	11	203
Burduja 2020 (30)	319	744	94.36	95.76	301	18	18	407
Kumaravel 2021 (31)	205	491	99.86	99.86	205	0	0	286
Wang 2021 (32)	879	2214	95.00	94.40	835	75	44	1260
Salehinejad 2021 (33)	674	5965	91.30	94.10	615	312	59	4979
Goyal 2022 (34)	100	200	95.00	99.99	95	0	5	100
Alfaer 2022 (35)	36	72	88.60	98.30	32	1	4	35
Dyer 2022 (36)	82	279	98.80	92.50	81	15	1	182
Gibson 2022 (37)	8329	28160	86.00	92.00	7163	1586	1166	18245
Hopkins 2022 (38)	33	726	94.00	94.00	31	42	2	651
Altuve 2022 (39)	100	200	95.20	96.20	95	4	5	96
Kyung 2022 (40)	34	73	97.10	74.40	33	10	1	29
Gudadhe 2022 (41)	125	250	86.30	86.80	108	17	17	109
Cortes-Ferre 2023 (42)	142	947	91.40	94.00	130	48	12	757
Asif 2023 (43)	205	491	95.40	97.20	196	8	9	278
Abrigo 2023 (44)	84	1372	73.00	79.00	62	271	22	1017
Yeo 2023 (45)	205	491	95.00	81.00	195	54	10	232
Kang 2023 (46)	487	1473	92.80	85.70	452	141	35	845
Umapathy 2023 (47)	205	491	97.00	99.00	198.85	3	6	283
Gamble 2024 (48)	2139	3650	92.7	87.9	1952	187	153	1358

Table S3: Characteristics of SAH studies tested on private datasets

Study	Events	Total	Sensitivity	Specificity	TP	FP	FN	TN
Li 2012 (49)	30	69	99.99	92.4	30	3	0	36
Li 2012 (50)	30	69	99.99	89.7	30	4	0	35
Chang 2018 (2)	21	103	90.5	90.5	19	8	2	74
Ye 2019 (4)	86	194	65	94	56	6	30	102
Nishi 2021 (51)	135	331	99	92	134	16	1	180
Alis 2022 (12)	48	298	91.7	86	44	35	4	215

Warman 2022 (17)	157	292	83.2	94.6	131	7	26	128
Nada 2025 (27)	399	5600	93.95	95.77	375	24	175	4980

Table S4: Characteristics of SAH studies tested on public datasets

Study	Events	Total	Sensitivity	Specificity	TP	FP	FN	TN
Chilamkurthy 2018 (29)	60	346	91.7	86.5	55	39	5	247
Burduja 2020 (30)	144	569	69.4	97.8	100	9	44	416
Wang 2021 (32)	383	1718	94.6	81.5	362	247	21	1088
Salehinejad 2021 (33)	674	5965	85.4	97.0	246	169	42	5508
Gibson 2022 (37)	3620	23451	84.0	78.0	3041	4363	579	15468
Asif 2023 (43)	60	346	94.3	95.1	57	14	3	272
Thanellas 2023 (52)	37	279	97.0	86.0	36	34	1	208
Abrigo 2023 (44)	34	1322	75.0	82.0	26	232	9	1056
Yeo 2023 (45)	60	346	96.0	73.0	58	77	2	209
Kang 2023 (46)	82	1068	97.4	82.6	80	172	2	814
Umapathy 2023 (47)	60	346	99.0	99.0	59	3	1	283

Table S5: Characteristics of IPH studies tested on private datasets

Study	Events	Total	Sensitivity	Specificity	TP	FP	FN	TN
Chang 2018 (2)	23	105	99.99	99.99	23	0	0	82
Ye 2019 (4)	145	194	90.00	82.00	131	9	15	40
Alis 2022 (12)	86	336	82.60	82.60	71	44	15	207
Warman 2022 (17)	196	331	90.30	96.90	177	4	19	131
Schmitt 2022 (14)	47	128	98.00	89.00	46	9	1	72
Nada 2025 (27)	491	5600	92.46	95.77	454	234	37	4875

Table S6: Characteristics of IPH studies tested on public datasets

Study	Events	Total	Sensitivity	Specificity	TP	FP	FN	TN
Chilamkurthy 2018 (29)	134	420	94.80	81.20	127	54	7	232
Burduja 2020 (30)	191	616	94.40	95.80	180	18	11	407
Wang 2021 (32)	525	1860	96.50	95.90	507	55	18	1280
Salehinejad 2021 (33)	287	5965	89.20	96.70	256	189	31	5489
Gibson 2022 (37)	4853	24684	88.00	84.00	4271	3173	582	16658
Asif 2023 (43)	134	420	94.10	93.50	126	19	8	267
Abrigo 2023 (44)	40	1328	77.00	88.00	31	155	9	1133
Yeo 2023 (45)	134	420	99.00	83.00	133	49	1	237
Kang 2023 (46)	266	1252	97.4	82.6	259	172	7	814
Umapathy 2023 (47)	134	420	97.00	99.00	130	3	4	283

Table S7: Characteristics of IVH studies tested on private datasets

Study	Events	Total	Sensitivity	Specificity	TP	FP	FN	TN
Ye 2019 (4)	67	194	66.00	94.00	44	8	23	119

Alis 2022 (12)	38	288	86.80	86.80	33	33	5	217
Warman 2022 (17)	119	254	82.30	98.20	98	2	21	133
Nada 2025 (27)	215	5600	96.23	95.75	207	242	8	5143

Table S8: Characteristics of IVH studies tested on public datasets

Study	Events	Total	Sensitivity	Specificity	TP	FP	FN	TN
Chilamkurthy 2018 (29)	28	314	92.90	66.50	26	96	2	190
Burduja 2020 (30)	126	551	91.00	99.50	115	2	11	423
Wang 2021 (32)	379	1714	94.60	97.00	359	40	20	1295
Salehinejad 2021 (33)	128	5965	87.50	98.80	112	68	16	5769
Gibson 2022 (37)	3253	23084	90.00	84.00	2928	3173	325	16658
Asif 2023 (43)	28	314	93.40	95.20	26	14	2	272
Abrigo 2023 (44)	13	1301	79.00	89.00	10	142	3	1146
Yeo 2023 (45)	28	314	95.00	96.00	27	11	1	275
Kang 2023 (46)	8	994	97.4	82.6	8	172	0	814
Umapathy 2023 (47)	28	314	96.00	99.00	27	3	1	283

Table S9: Characteristics of SDH studies tested on private datasets

Study	Events	Total	Sensitivity	Specificity	TP	FP	FN	TN
Ye 2019 (4)	36	194	61	93	22	11	14	147
Alis 2022 (12)	76	326	88.2	90.2	67	25	9	226
Warman 2022 (17)	204	339	88.00	96.10	180	5	24	130
Nada 2025 (27)	457	5600	89.05	95.74	407	219	50	4924

Table S10: Characteristics of SDH studies tested on public datasets

Study	Events	Total	Sensitivity	Specificity	TP	FP	FN	TN
Chilamkurthy 2018 (29)	53	339	94.60	71.00	50	83	3	203
Burduja 2020 (30)	143	568	72.00	97.60	103	10	40	415
Wang 2021 (32)	370	1705	88.70	88.00	328	160	42	1175
Salehinejad 2021 (33)	367	5965	89.70	97.00	329	169	38	5429
Gibson 2022 (37)	3472	23303	76.00	83.00	2639	3371	833	16460
Asif 2023 (43)	53	339	95.10	95.30	50	13	3	273
Abrigo 2023 (44)	32	1320	74.00	83.00	24	219	8	1069
Yeo 2023 (45)	53	339	94.00	73.00	50	77	3	209
Kang 2023 (46)	15	1001	88.40	82.60	13	172	2	814
Umapathy 2023 (47)	53	339	98.00	99.00	52	3	1	283

Table S11: Characteristics of EDH studies tested on private datasets

Study	Events	Total	Sensitivity	Specificity	TP	FP	FN	TN
Ye 2019 (4)	13	194	69.00	94.00	9	11	4	170
Alis 2022 (12)	14	264	71.40	99.90	10	0	4	250
Warman 2022 (17)	23	158	78.60	97.20	18	4	5	131

Nada 2025 (27)	33	5600	93.94	95.74	31	237	2	5330

Table S12: Characteristics of EDH studies tested on public datasets

Study	Events	Total	Sensitivity	Specificity	TP	FP	FN	TN
Chilamkurthy 2018 (29)	13	299	92.30	88.30	12	33	1	253
Burduja 2020 (30)	10	435	40.00	99.70	4	1	6	424
Wang 2021 (32)	41	1376	95.60	88.50	39	154	2	1181
Salehinejad 2021 (33)	25	5965	28.00	99.80	7	14	18	5926
Gibson 2022 (37)	304	20135	56.00	83.00	170	3371	134	16460
Asif 2023 (43)	13	299	95.40	94.20	12	17	1	269
Yeo 2023 (45)	13	299	99.99	90.00	13	29	0	257
Kang 2023 (46)	1	987	88.40	82.60	1	172	0	814
Umapathy 2023 (47)	13	299	96.00	97.00	12	9	1	277

Table S13: Characteristics of FDA-approved ICH studies

Study	Events	Total	Sensitivity	Specificity	TP	FP	FN	TN
Ginat 2020 (5)	373	2011	88.70	94.20	331	95	42	1543
Voter 2021 (11)	349	3625	92.30	97.70	322	75	27	3201
Heit 2021 (8)	158	308	95.60	95.30	151	7	7	143
Rava 2021 (10)	200	302	93.00	93.00	186	7	14	95
Kau 2022 (16)	214	2188	68.20	96.80	146	63	68	1911
Eldaya 2022 (13)	37	307	91.90	84.40	34	42	3	228
Schmitt 2022 (14)	79	160	91.00	89.00	72	9	7	72
Warman 2022 (17)	391	526	94.50	92.10	369	11	22	124
Mair 2022 (15)	627	3266	94.00	83.00	588	89	39	2550
Neves 2023 (21)	402	510	97.50	99.99	392	0	10	108
Del Gaizo 2024 (23)	5656	58321	75.60	92.10	1192	4464	384	52281
Pettet 2024 (24)	165	1315	85.70	94.30	96	69	16	1134
Savage 2024 (25)	814	3716	87.80	94.30	645	169	90	2812
Nada 2025 (21)	1595	5600	89.00	96.00	1420	160	175	3845
Villringer 2025 (28)	38	257	90.00	96.00	29	9	4	215

Table S14: Characteristics of Non-FDA-approved ICH studies

Study	Events	Total	Sensitivity	Specificity	TP	FP	FN	TN
Prevedello 2017 (1)	50	85	90.00	85.00	45	5	5	30
Chang 2018 (2)	82	682	95.10	98.10	78	11	4	589
Kuo 2019 (3)	25	200	99.99	90.00	25	18	0	158
Ye 2019 (4)	194	299	99.00	99.00	192	1	2	104
Lee 2020 (6)	50	84	84.00	78.00	42	7	8	27
Karki 2020 (7)	441	541	89.80	98.50	396	2	45	99
Watanabe 2021 (9)	24	40	91.70	81.20	22	3	2	13
Ozaltin 2022 (18)	285	570	97.54	99.30	278	2	7	283
Alis 2022 (12)	167	547	96.41	95.79	161	16	6	364

Yun 2023 (53)	6442	49841	94.40	98.20	6081	781	361	42618
Chilamkurthy 2018 (29)	205	491	94.63	70.98	194	83	11	203
Burduja 2020 (30)	319	744	94.36	95.76	301	18	18	407
Kumaravel 2021 (31)	205	491	99.86	99.86	205	0	0	286
Wang 2021 (32)	879	2214	95.00	94.40	835	75	44	1260
Salehinejad 2021 (33)	674	5965	91.30	94.10	615	312	59	4979
Goyal 2022 (34)	100	200	95.00	99.99	95	0	5	100
Gudadhe 2022 (41)	125	250	86.30	86.80	108	17	17	109
Alfaer 2022 (35)	36	72	88.60	98.30	32	1	4	35
Kyung 2022 (54)	34	73	97.10	74.40	33	10	1	29
Dyer 2022 (36)	82	279	98.80	92.50	81	15	1	182
Gibson 2022 (37)	8329	28160	86.00	92.00	7163	1586	1166	18245
Hopkins 2022 (38)	33	726	94.00	94.00	31	42	2	651
Altuve 2022 (39)	100	200	95.20	96.20	95	4	5	96
Cortes-Ferre 2023 (42)	142	947	91.40	94.00	130	48	12	757
Abrigo 2023 (44)	84	1372	73.00	79.00	62	271	22	1017
Yeo 2023 (45)	205	491	95.00	81.00	195	54	10	232
Asif 2023 (43)	205	491	95.40	97.20	196	8	9	278
Wang 2023 (19)	79	527	92.00	96.00	73	18	6	430
Kang 2023 (46)	487	986	92.80	85.70	452	71	35	428
Umapathy 2023 (47)	205	491	97.00	99.00	198.85	3	6	283
Boujelb 2024 (22)	67	85	98	77	62	5	1	17
Gamble 2024 (48)	2139	3650	92.7	87.9	1952	187	153	1358
Fan 2025 (26)	807	1266	99.8	99.6	807	0	1	458

Figures of each ICH sub-group included in the Meta-Analysis

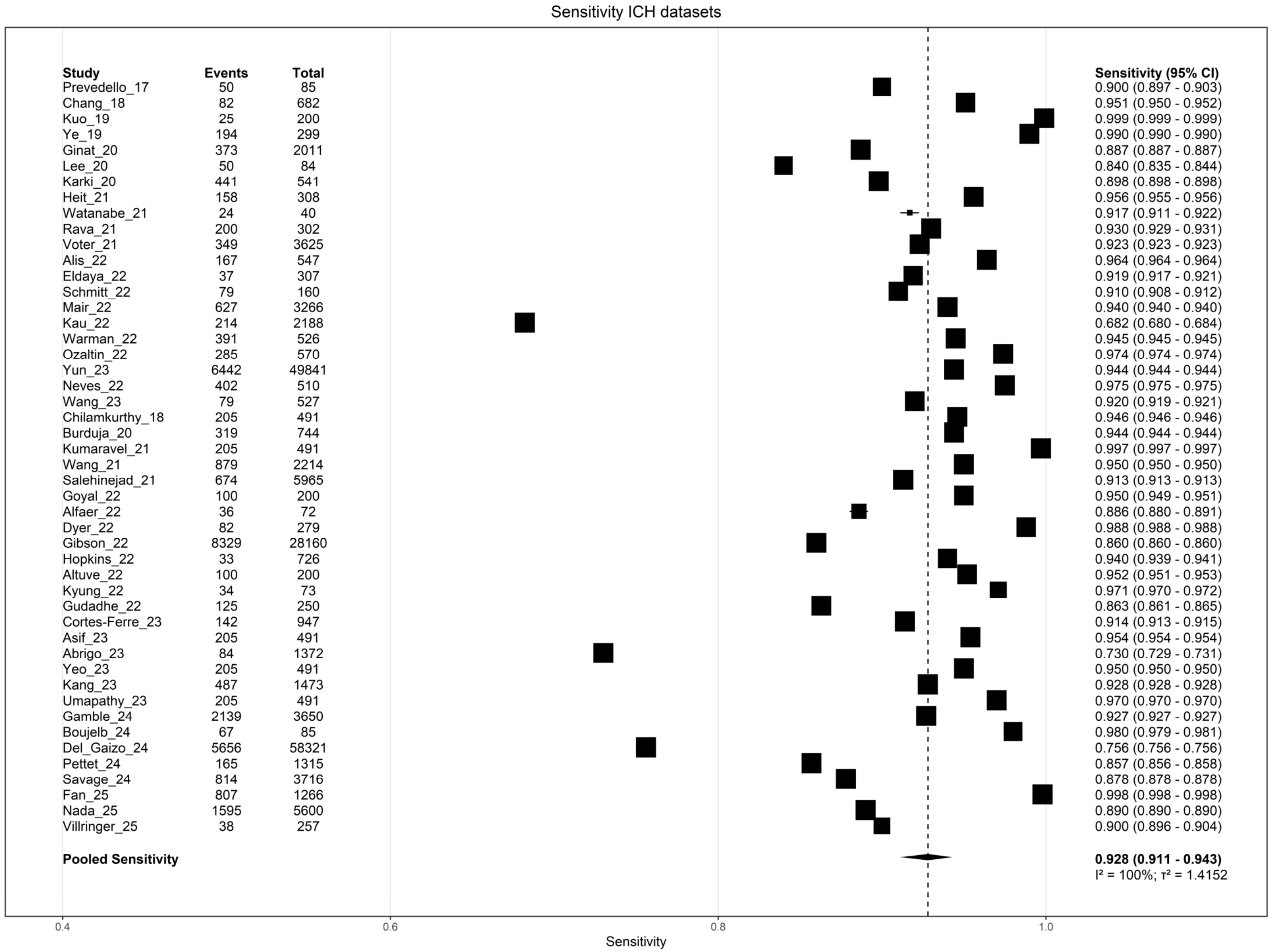

Figure S1. Sensitivity of ICH studies tested on both private and public datasets.

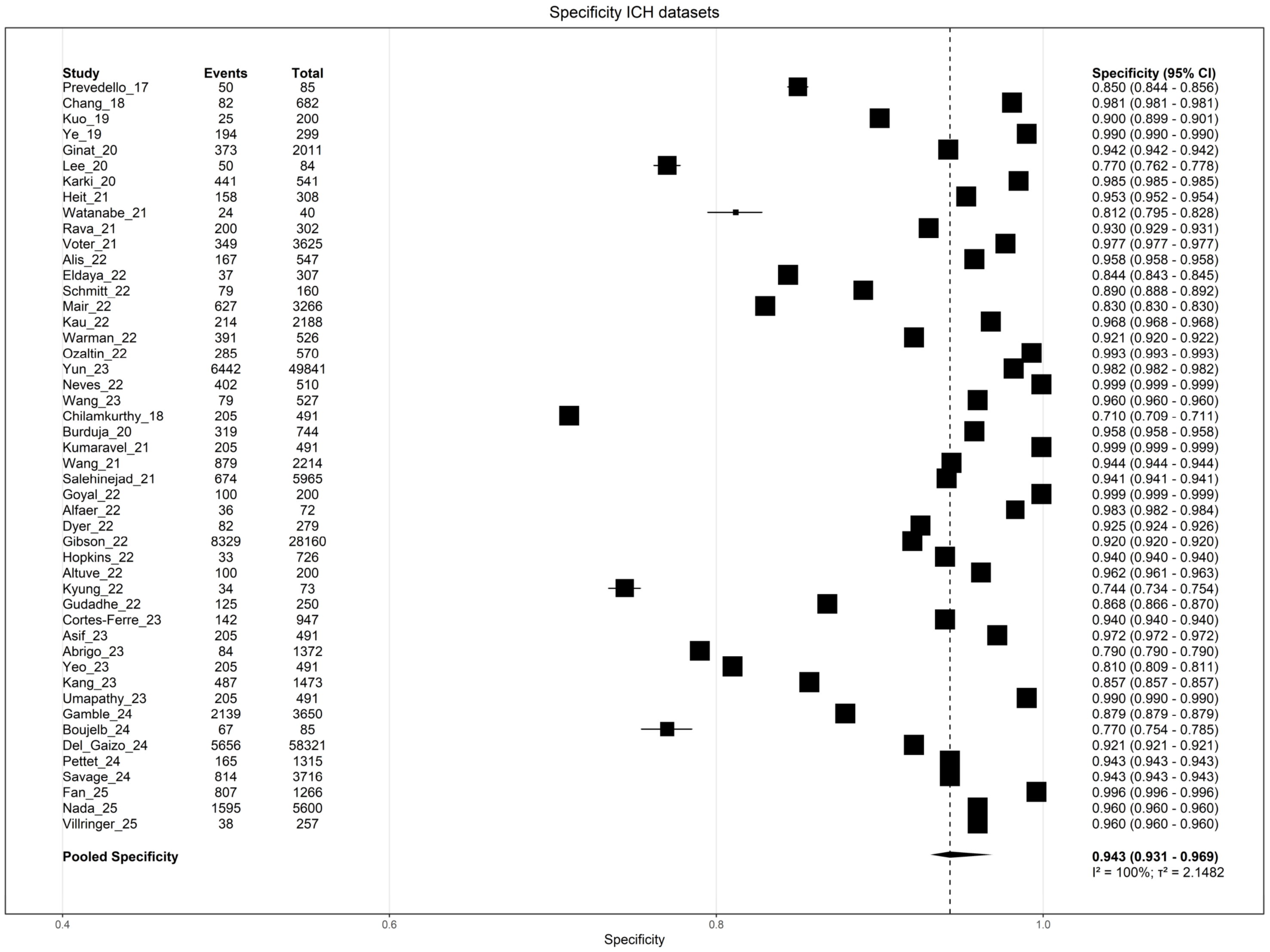

Figure S2. Specificity of ICH studies tested on both private and public datasets.

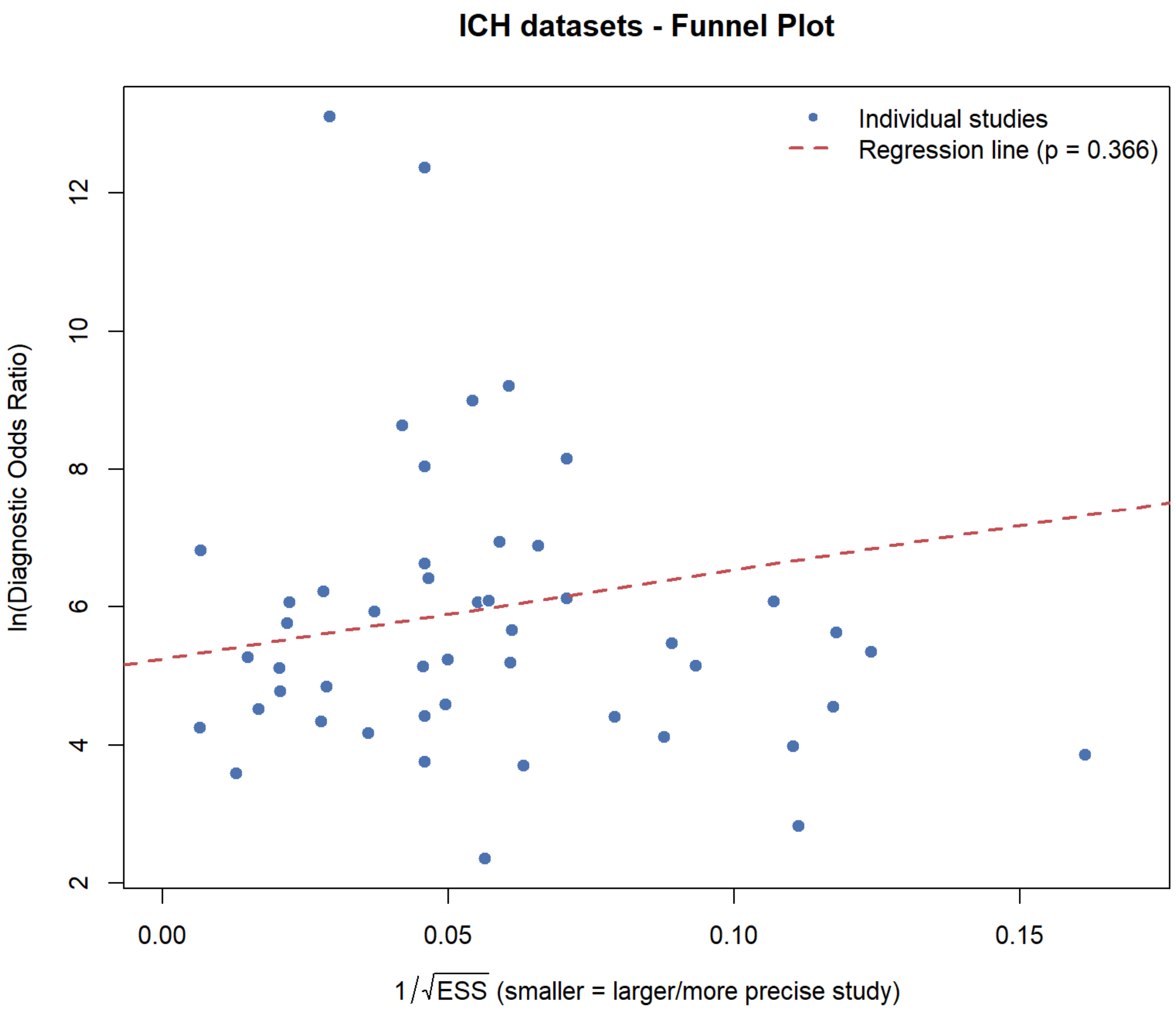

Figure S3. Funnel plot for ICH studies tested on both private and public datasets.

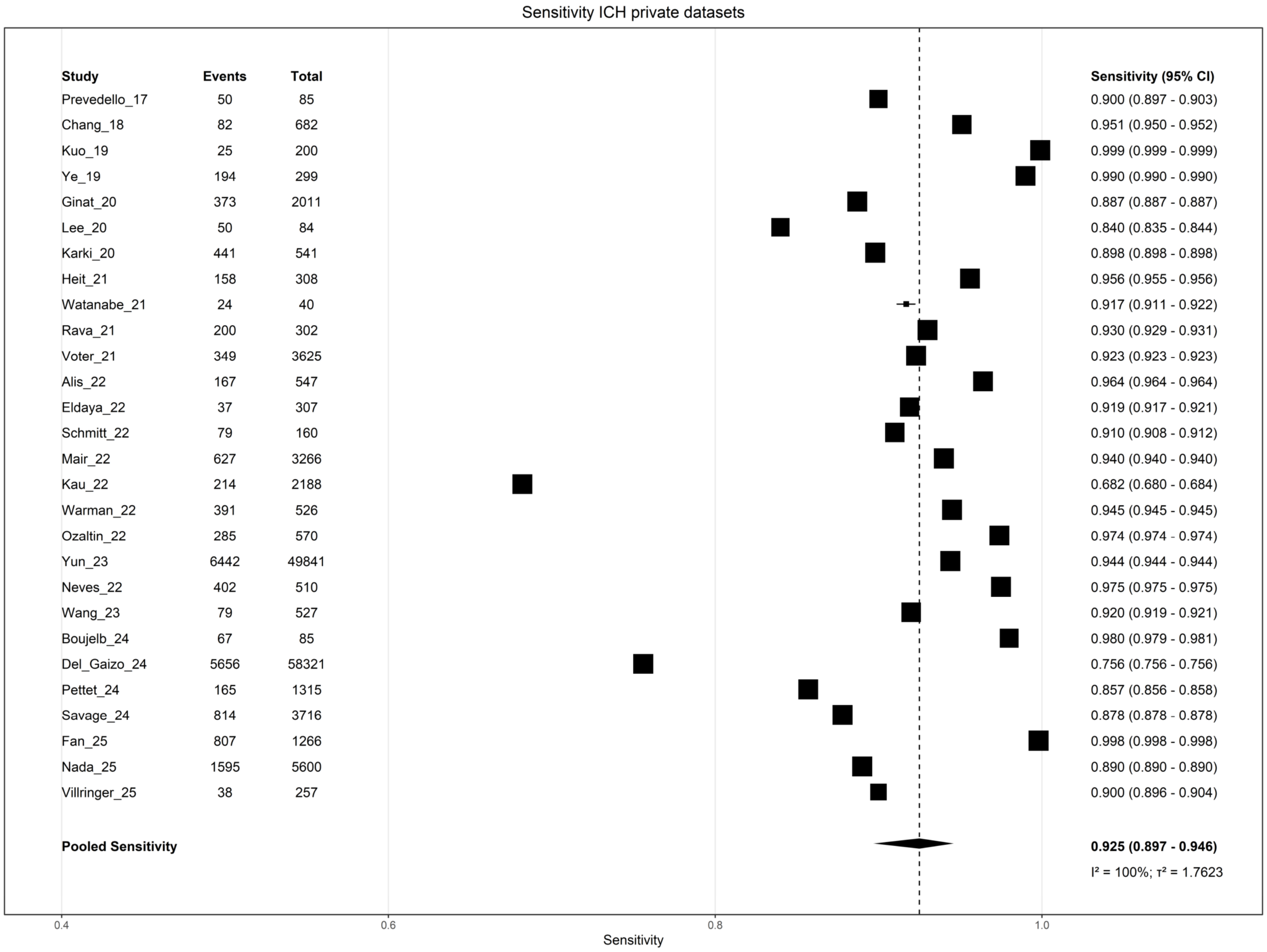

Figure S4. Sensitivity of ICH studies tested on private datasets.

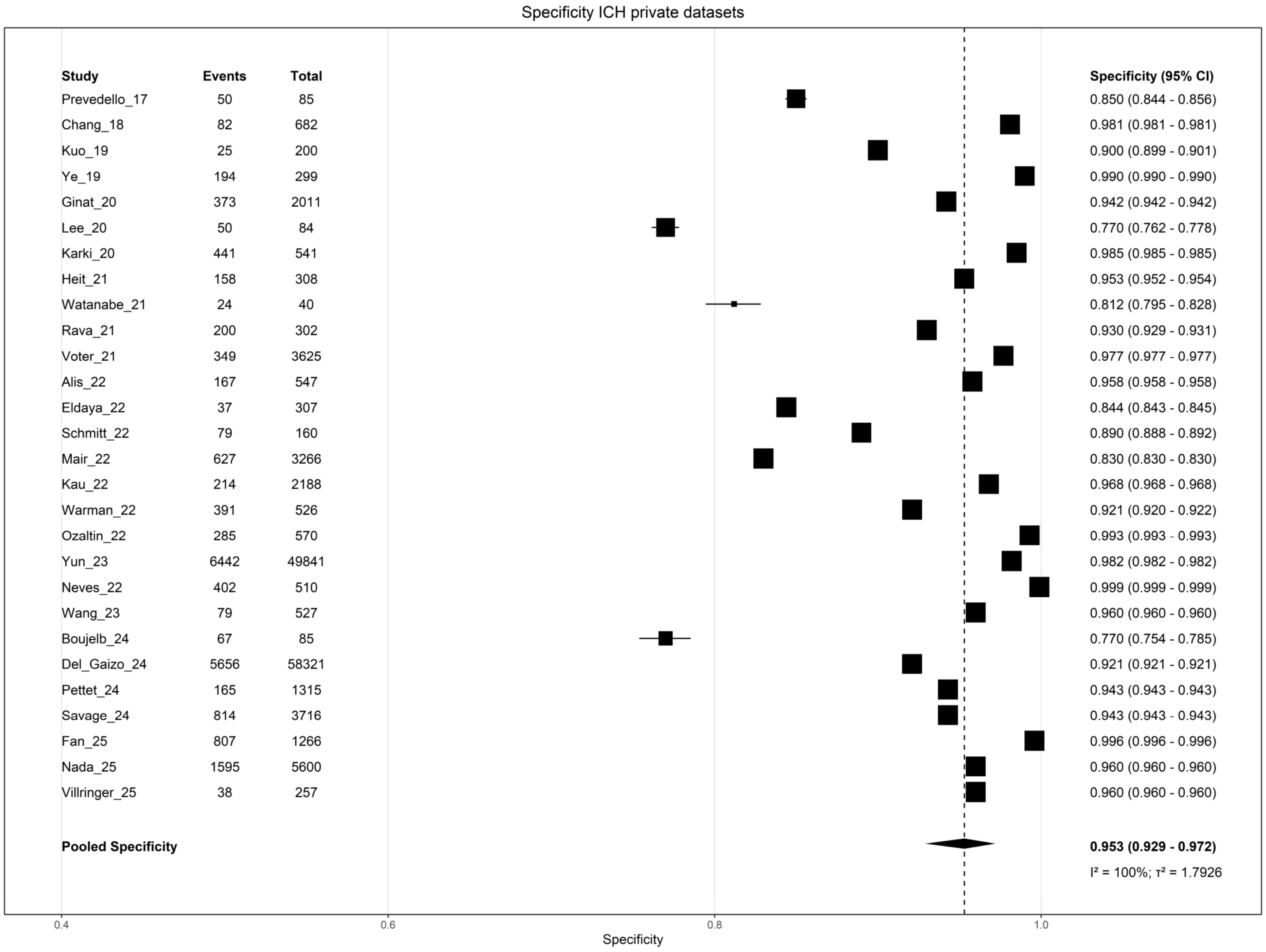

Figure S5. Specificity of ICH studies tested on private datasets.

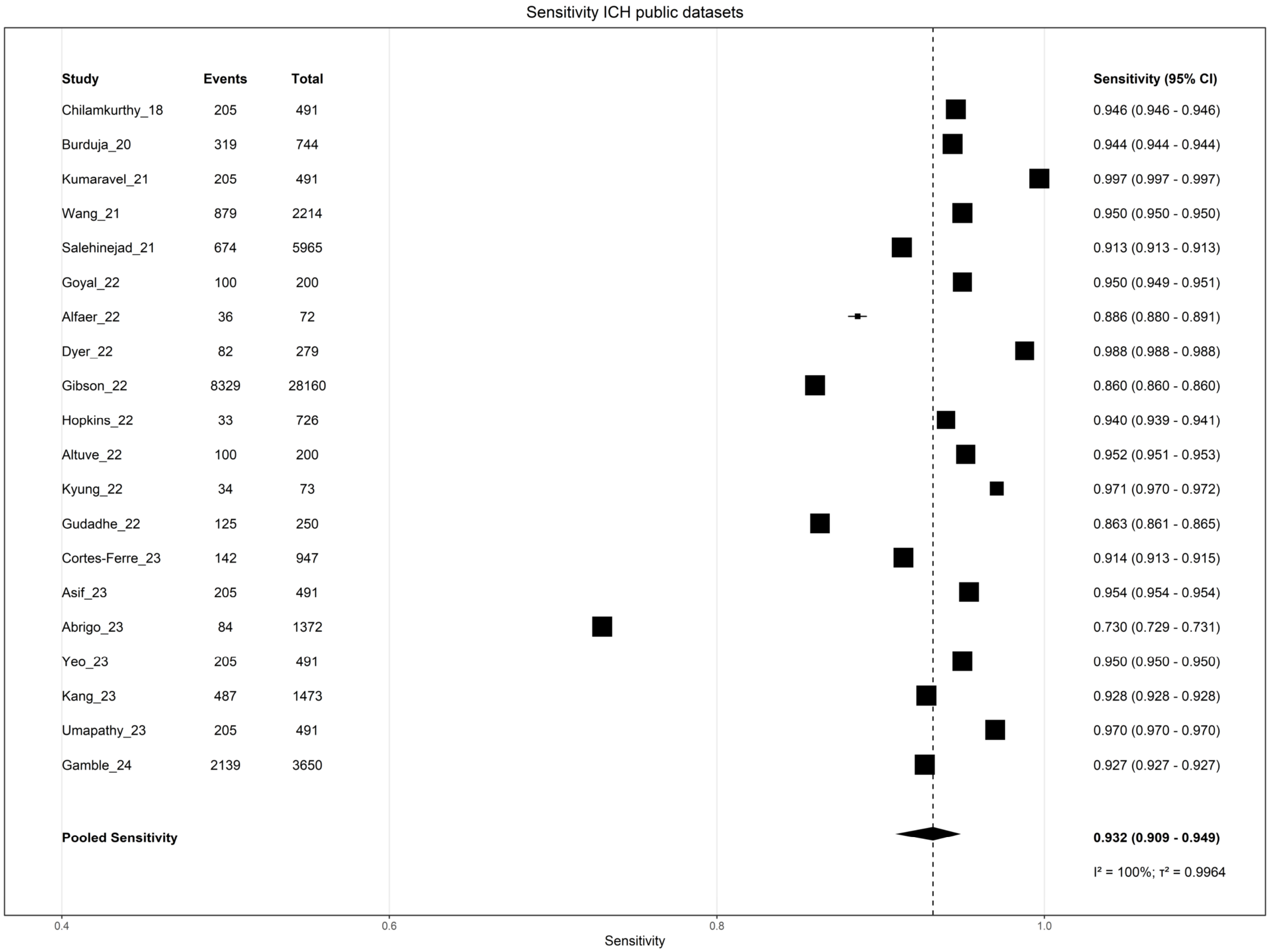

Figure S6. Sensitivity of ICH studies tested on public datasets.

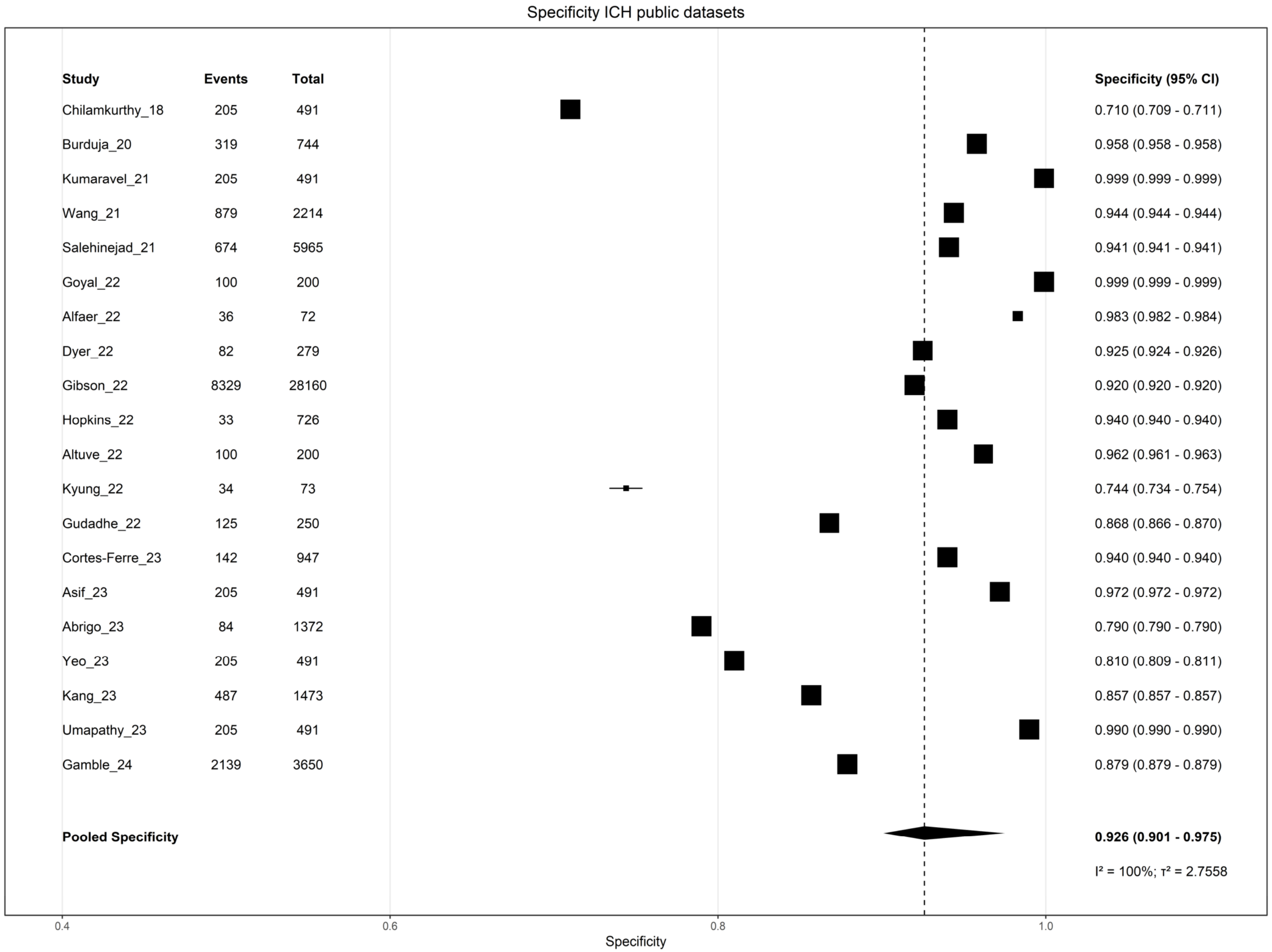

Figure S7. Specificity of ICH studies tested on public datasets.

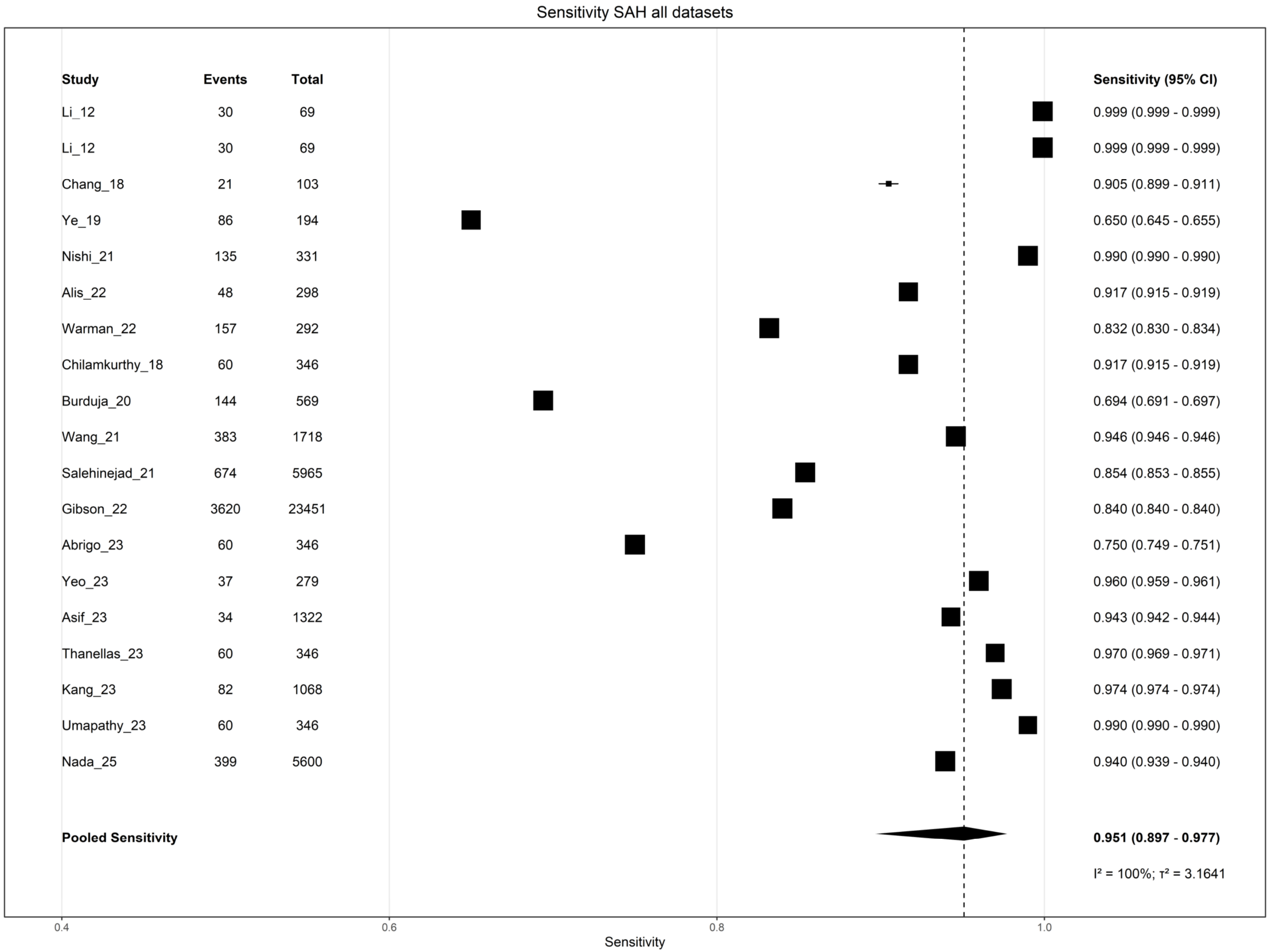

Figure S8. Sensitivity of SAH studies tested on both private and public datasets.

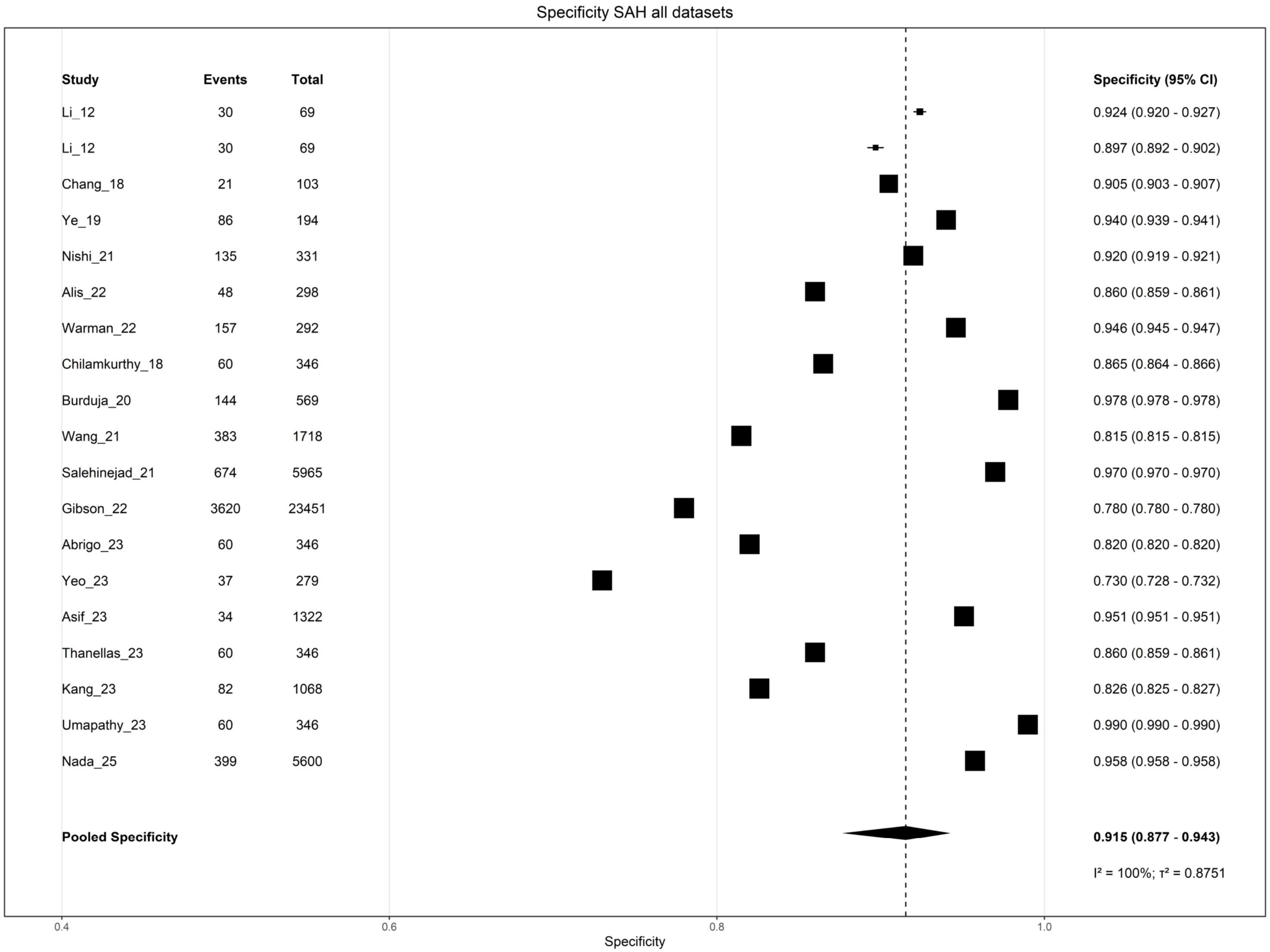

Figure S9. Specificity of SAH studies tested on both private and public datasets.

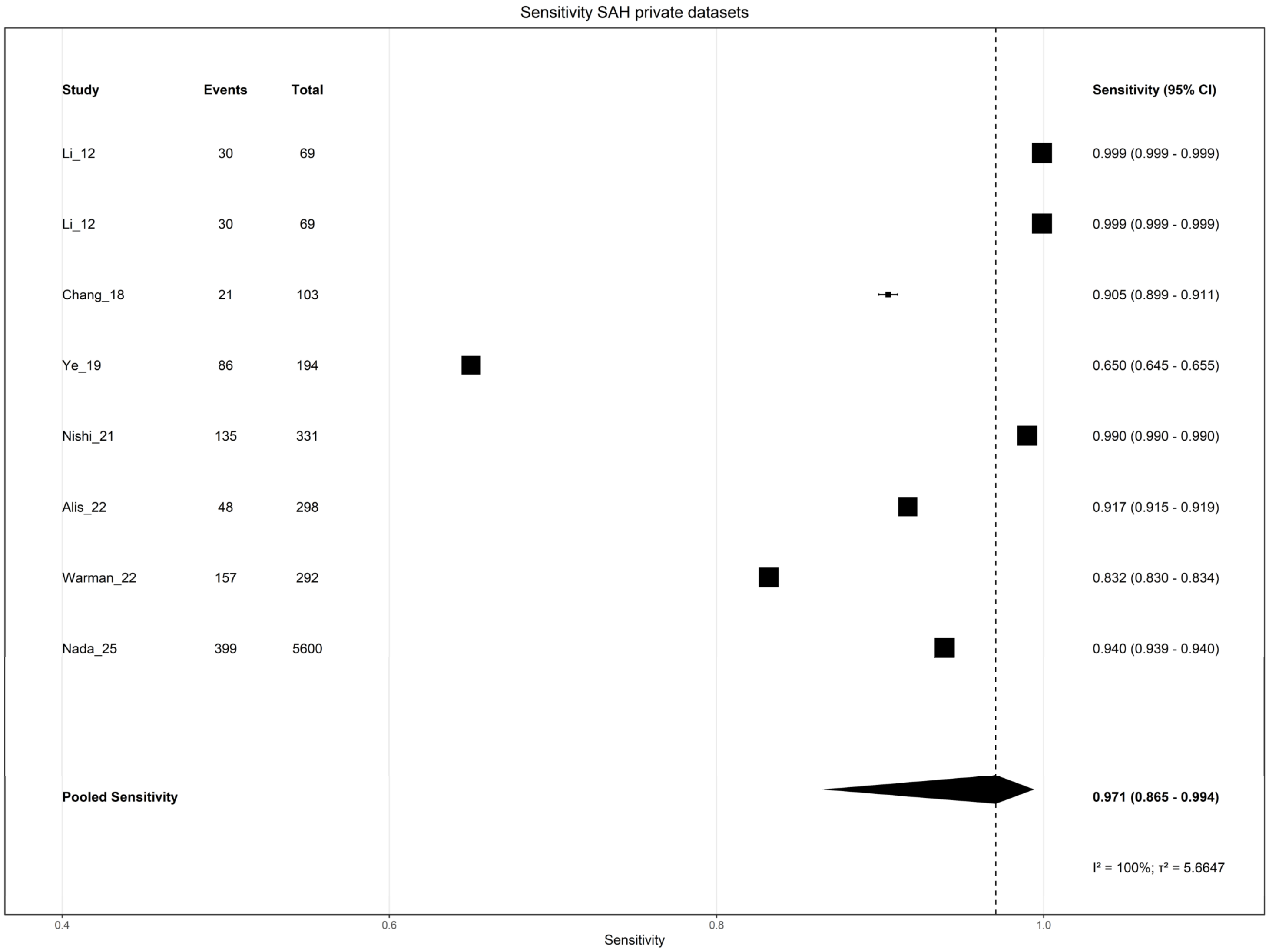

Figure S10. Sensitivity of SAH studies tested on private datasets.

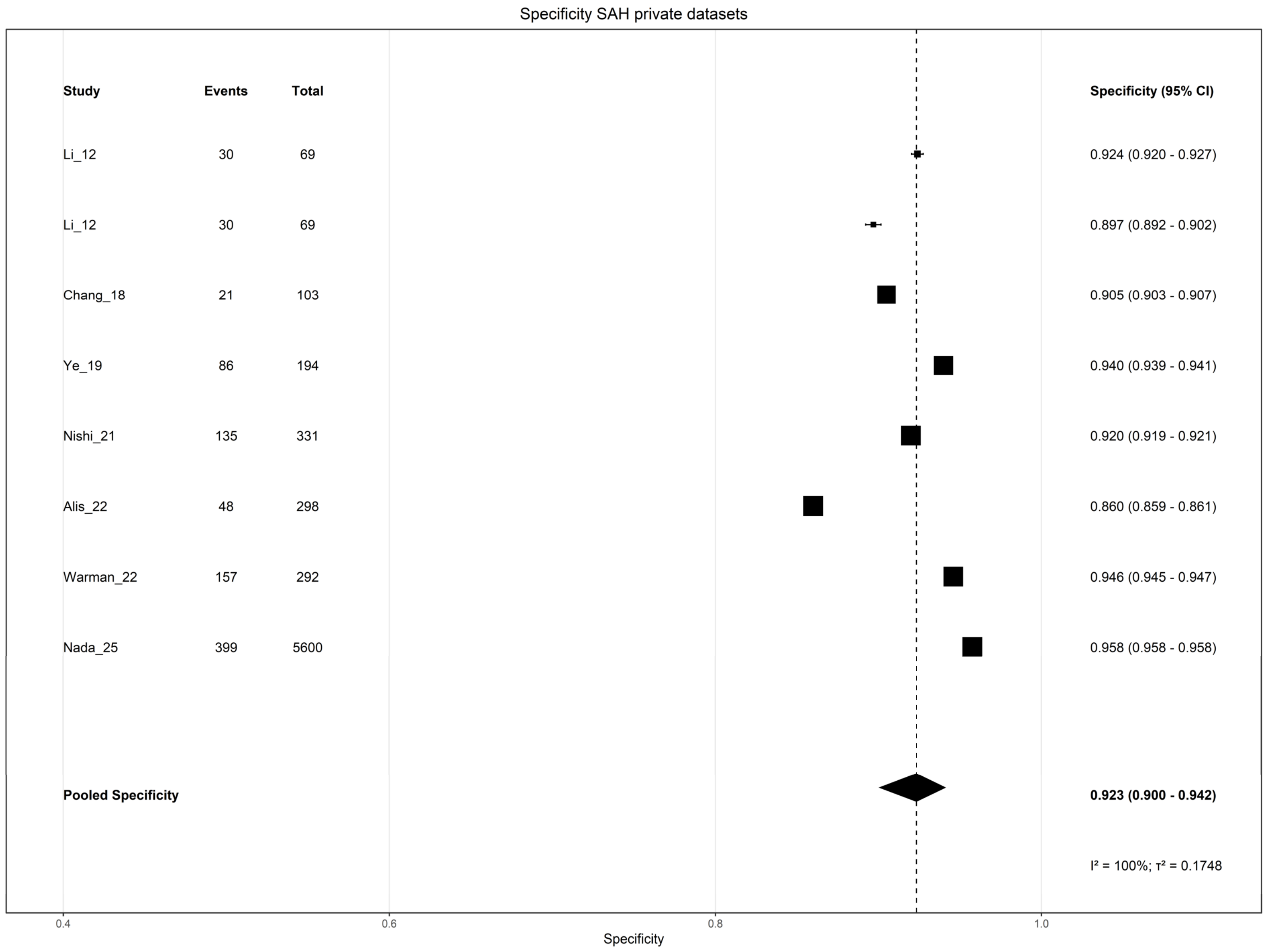

Figure S11. Specificity of SAH studies tested on private datasets.

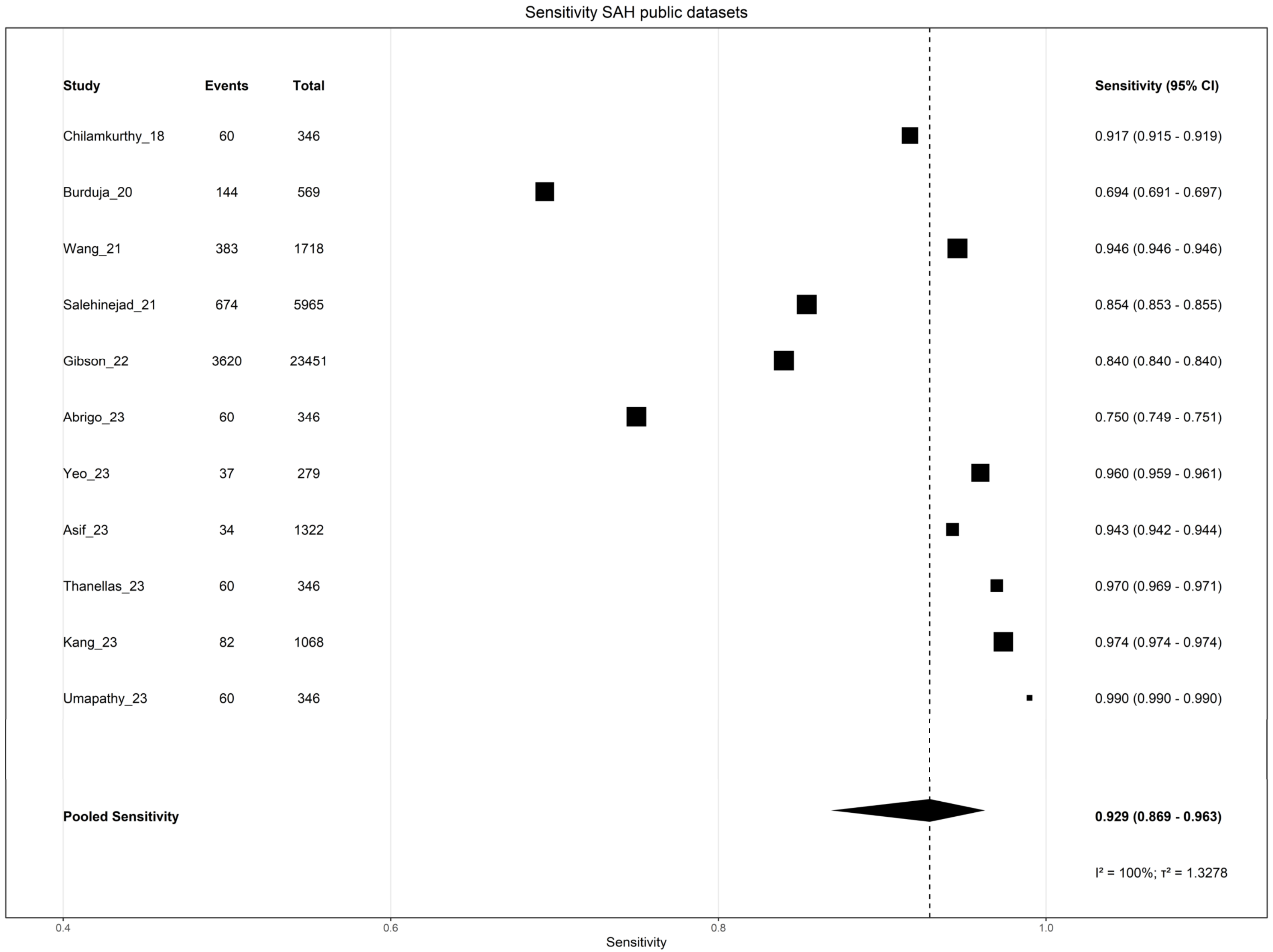

Figure S12. Sensitivity of SAH studies tested on public datasets.

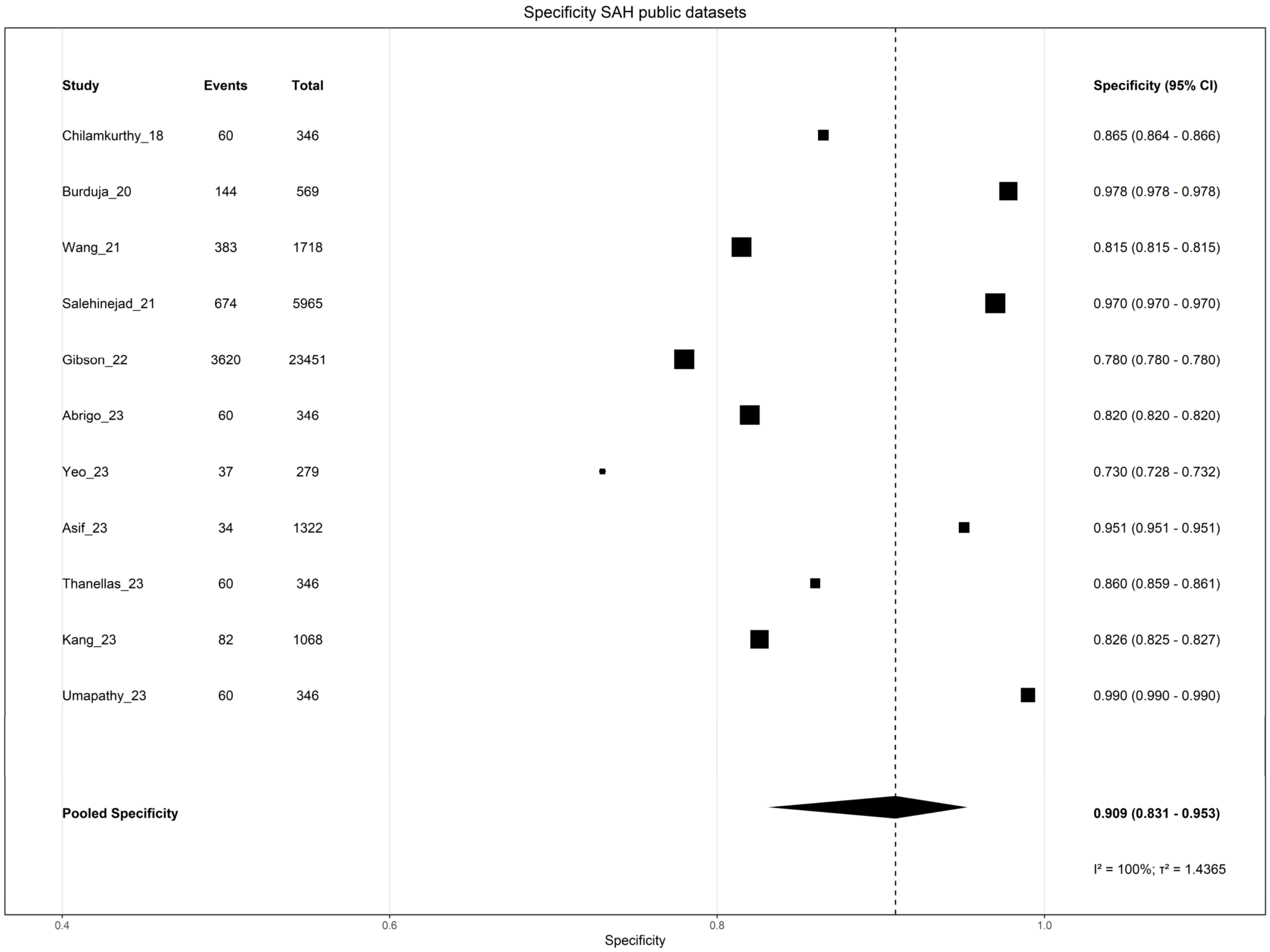

Figure S13. Specificity of SAH studies tested on public datasets.

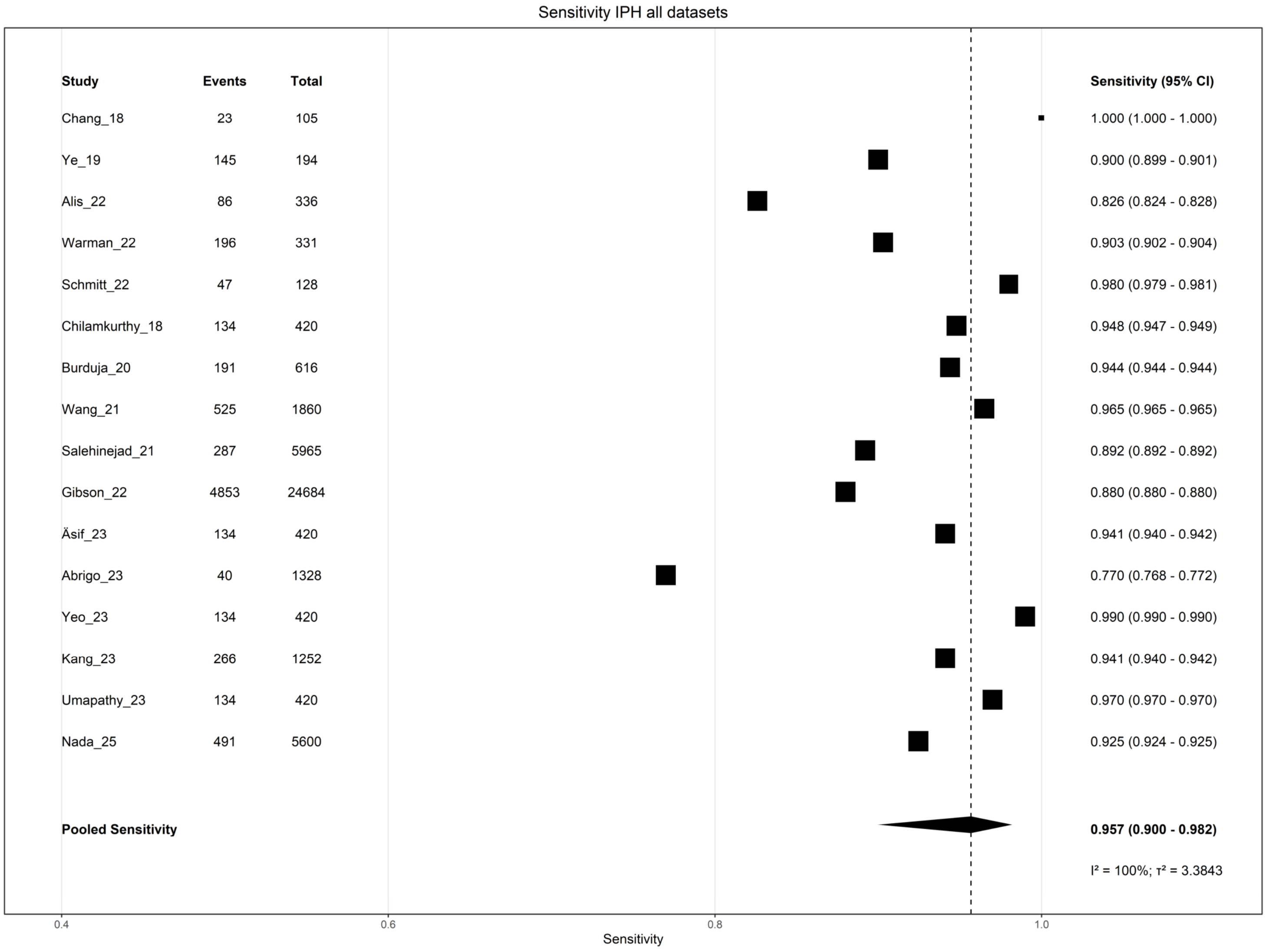

Figure S14. Sensitivity of IPH studies tested on both private and public datasets.

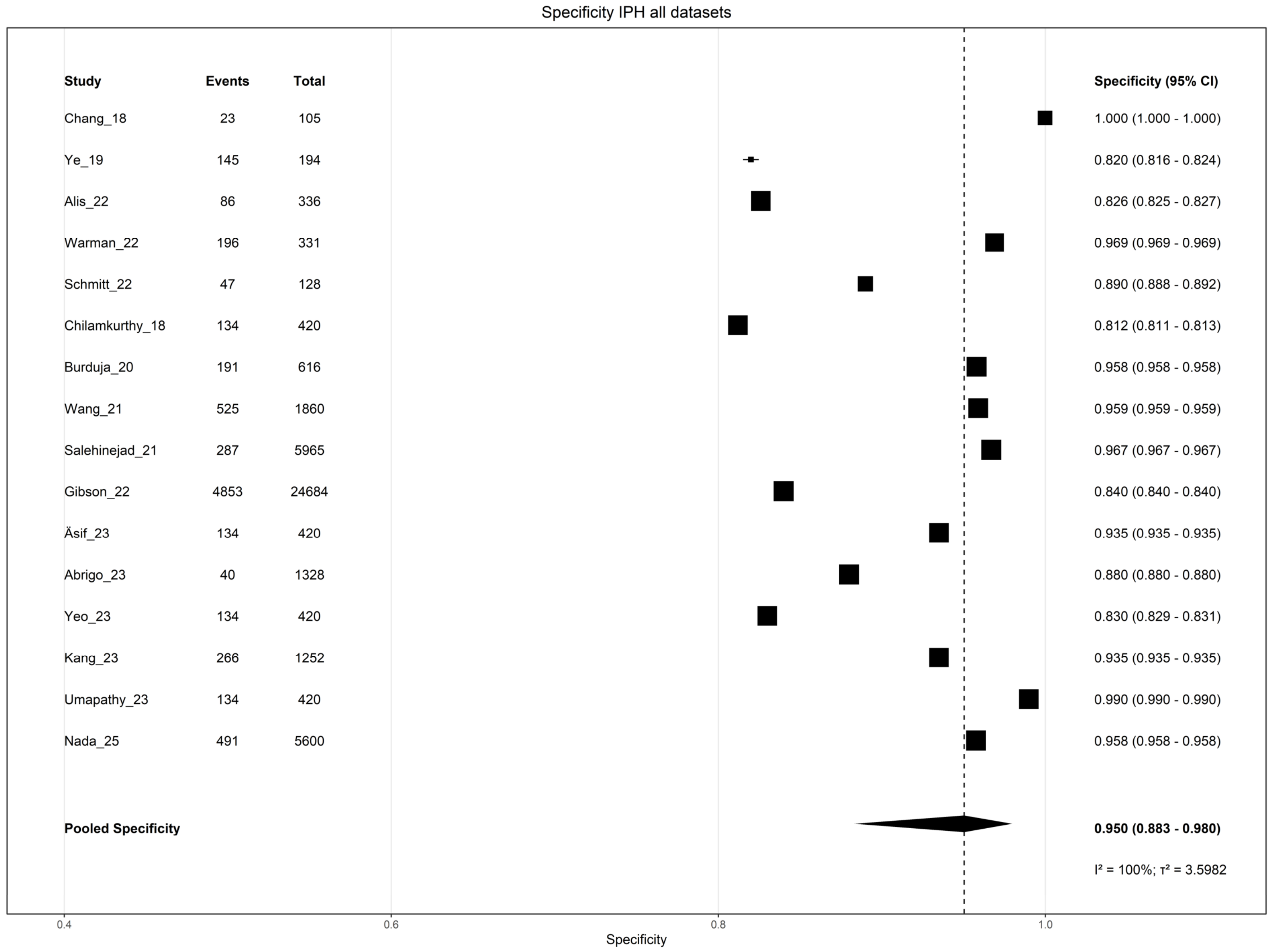

Figure S15. Specificity of IPH studies tested on both private and public datasets.

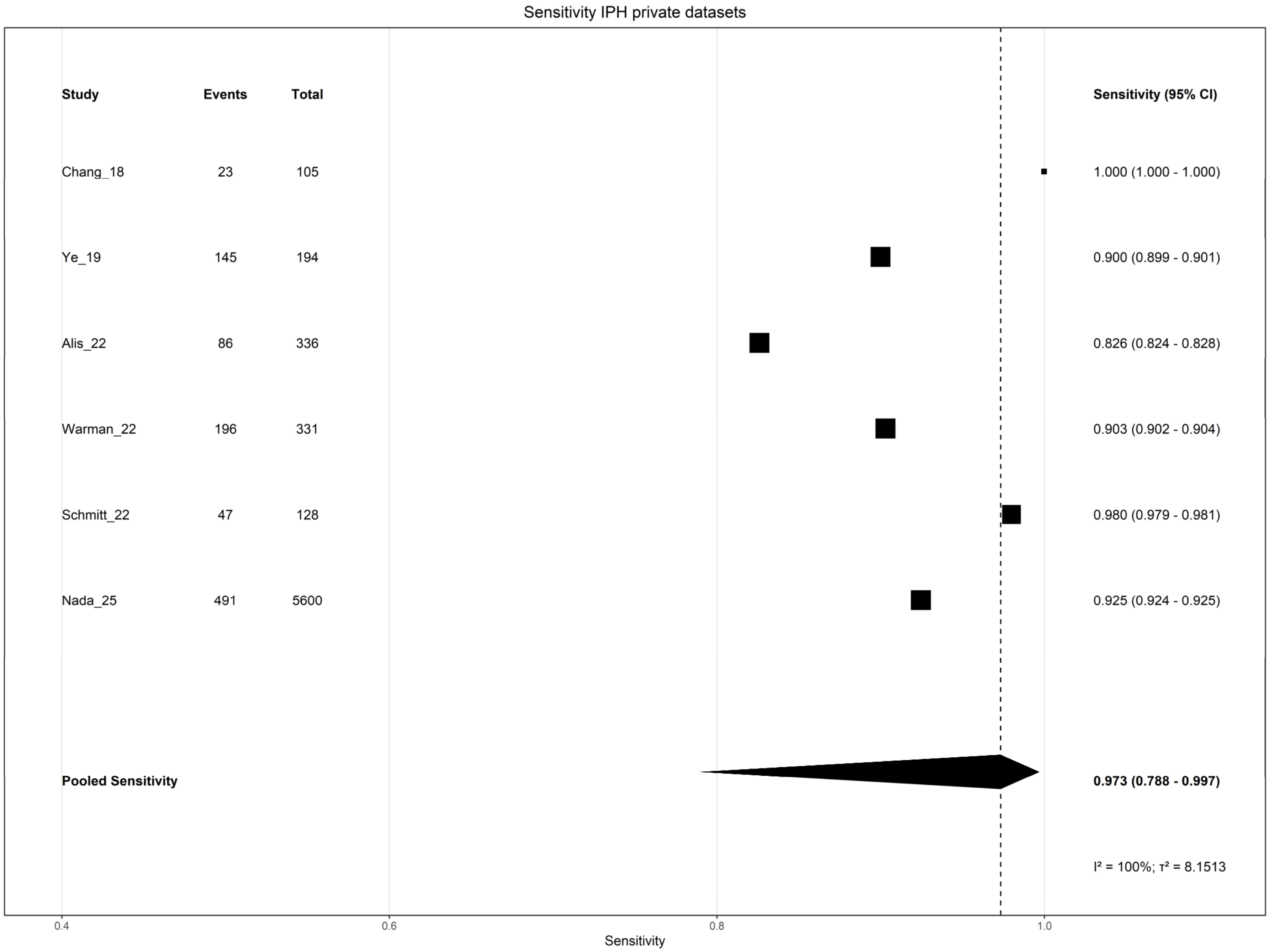

Figure S16. Sensitivity of IPH studies tested on private datasets.

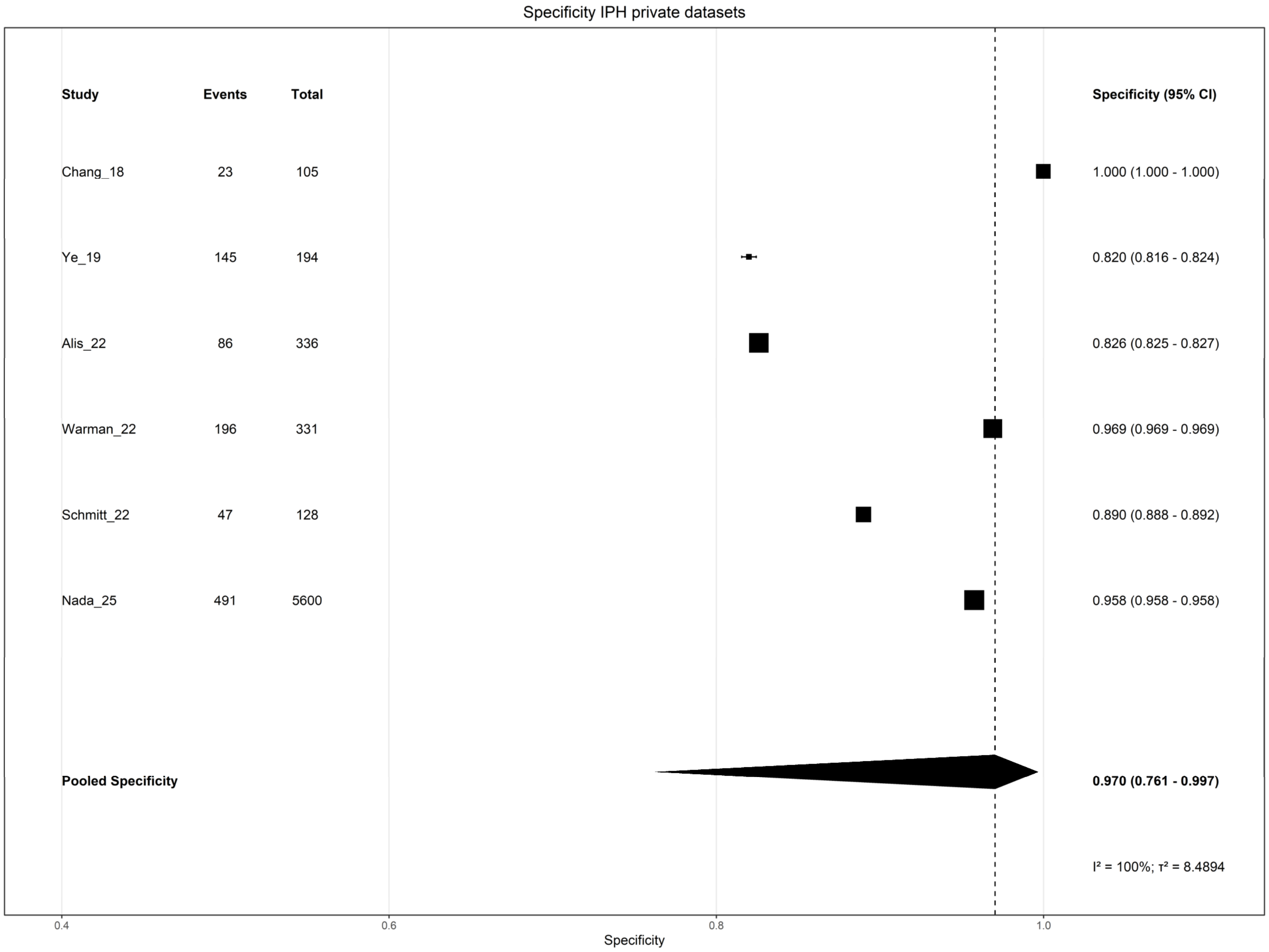

Figure S17. Specificity of IPH studies tested on private datasets.

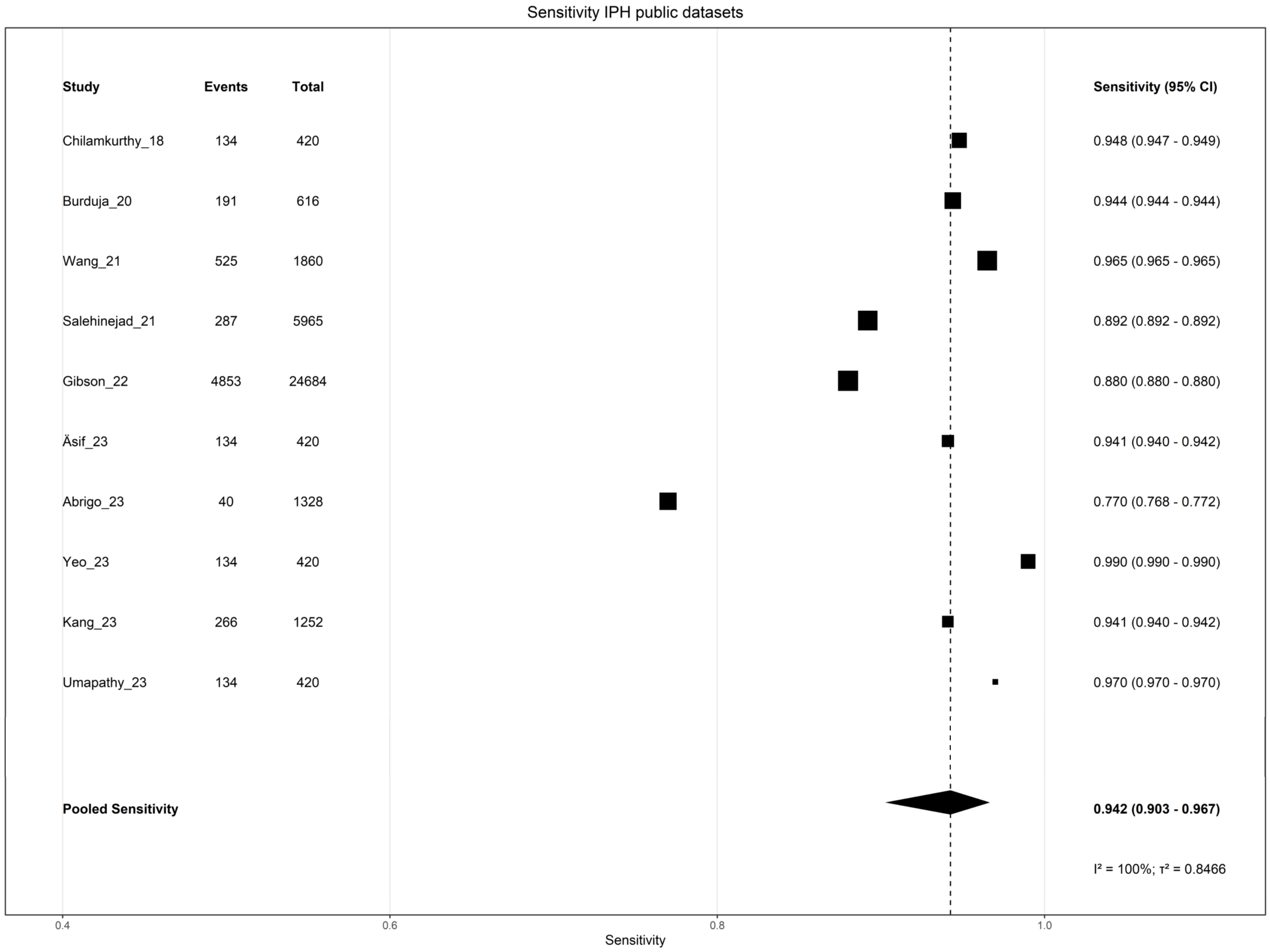

Figure S18. Sensitivity of IPH studies tested on public datasets.

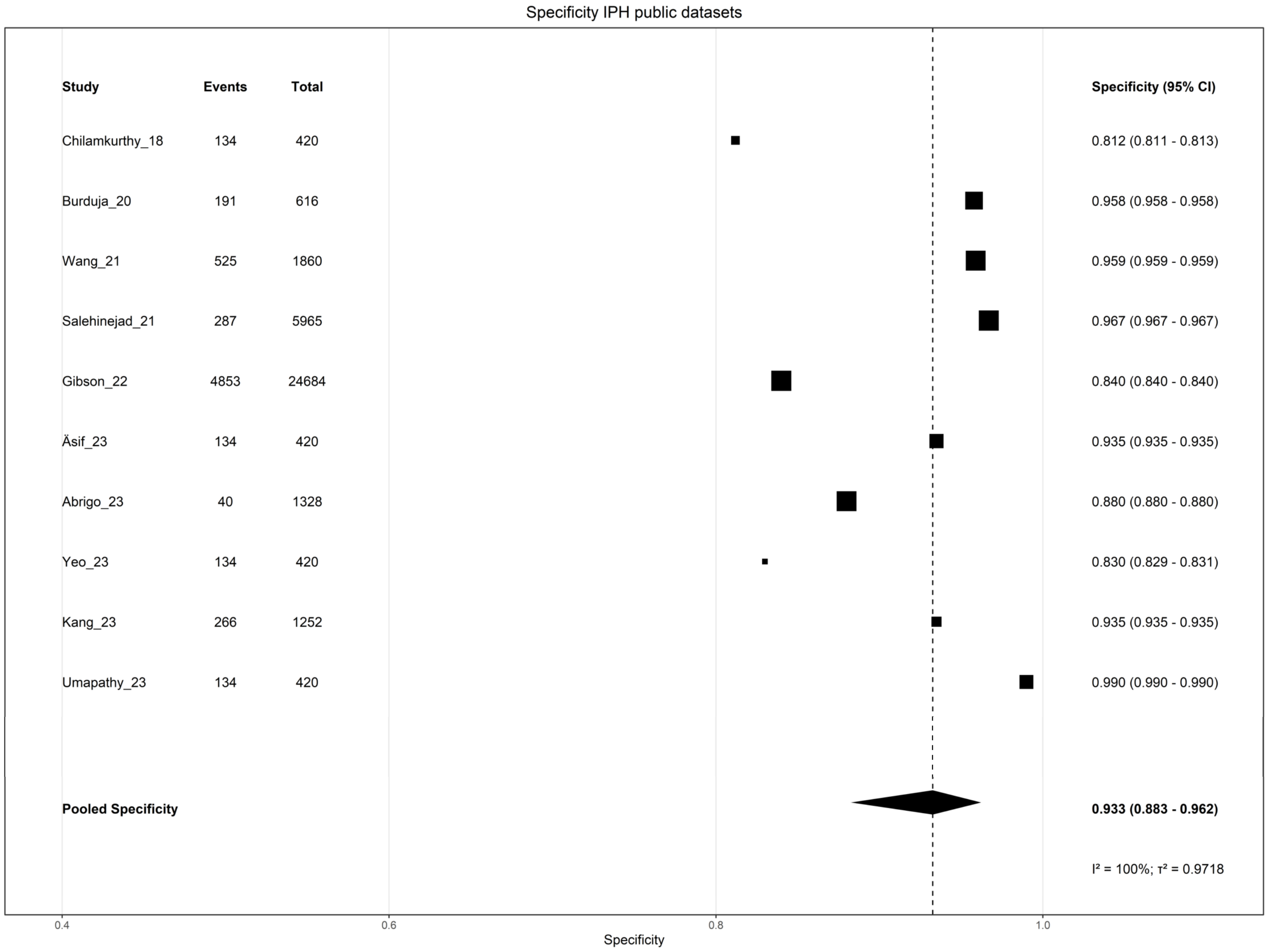

Figure S19. Specificity of IPH studies tested on public datasets.

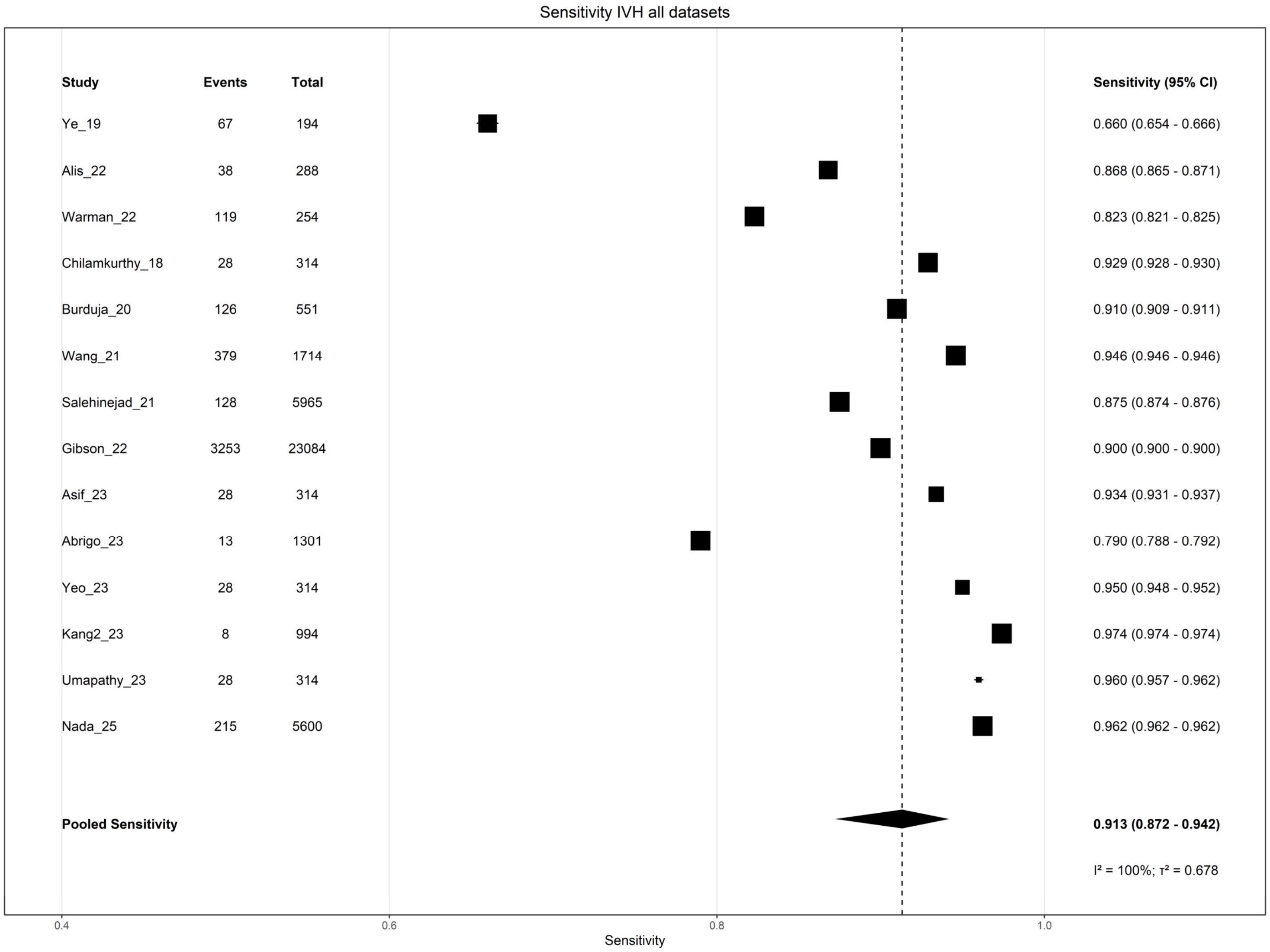

Figure S20. Sensitivity of IVH studies tested on both private and public datasets.

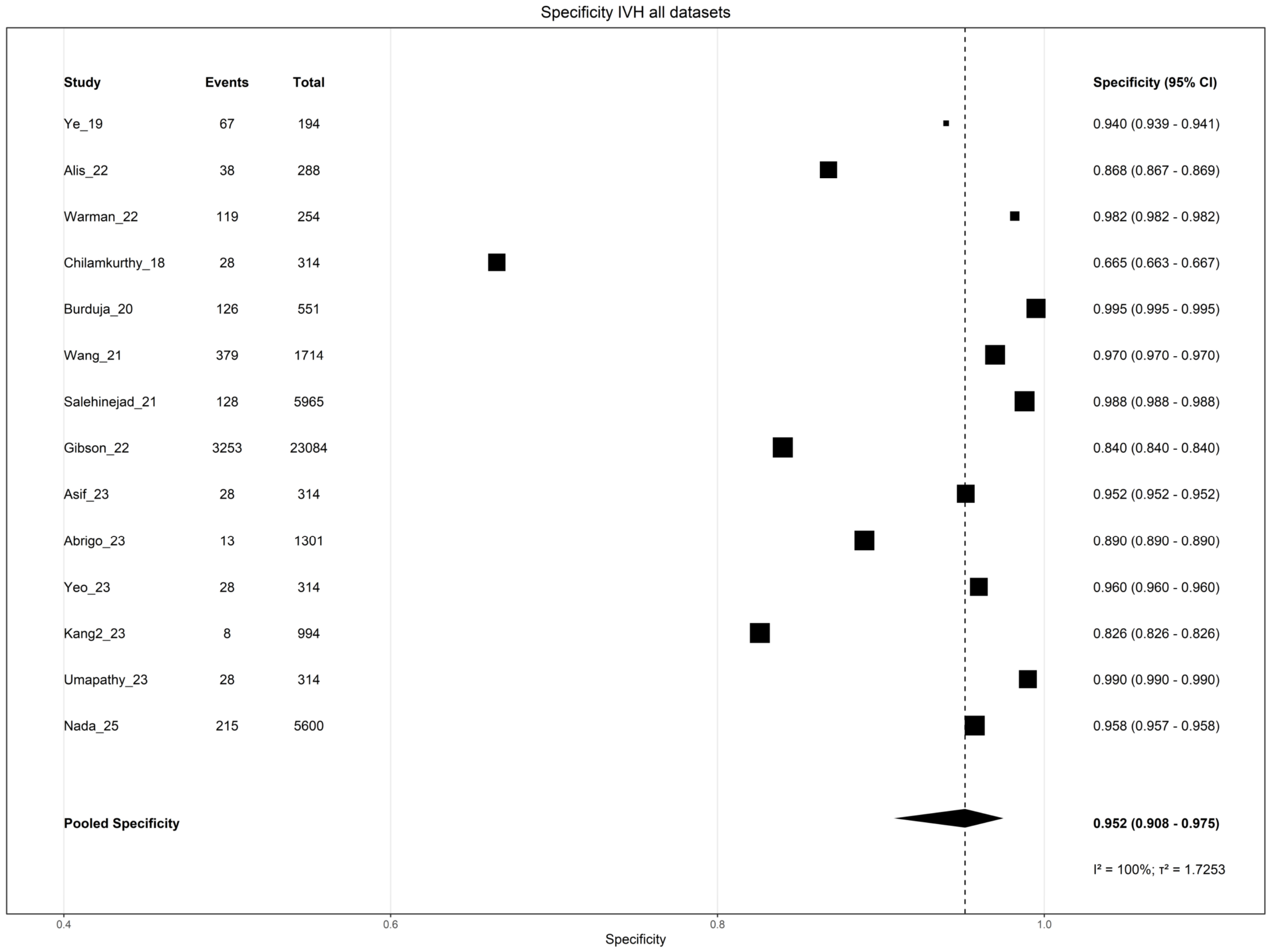

Figure S21. Specificity of IVH studies tested on both private and public datasets.

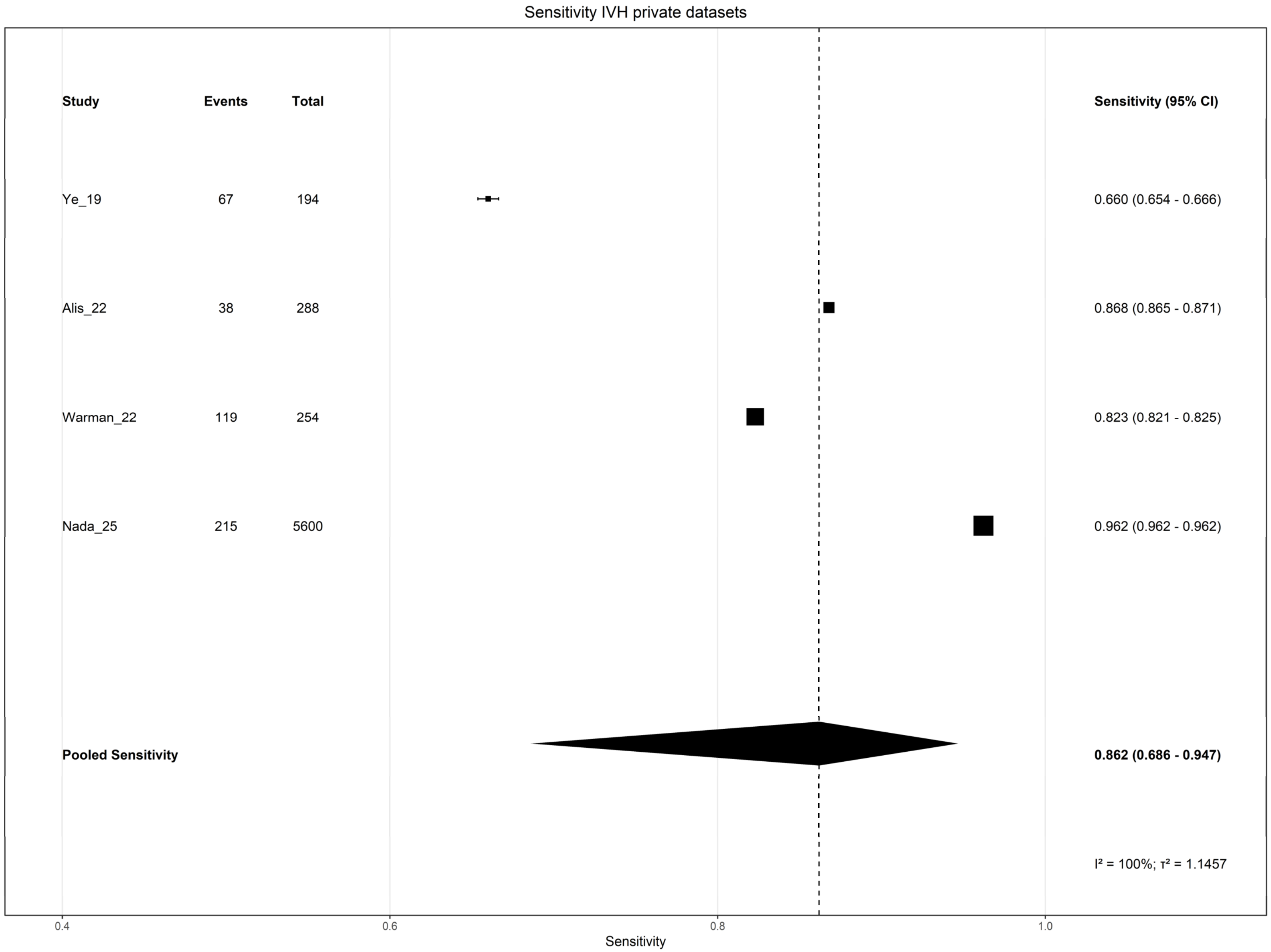

Figure S22. Sensitivity of IVH studies tested on private datasets.

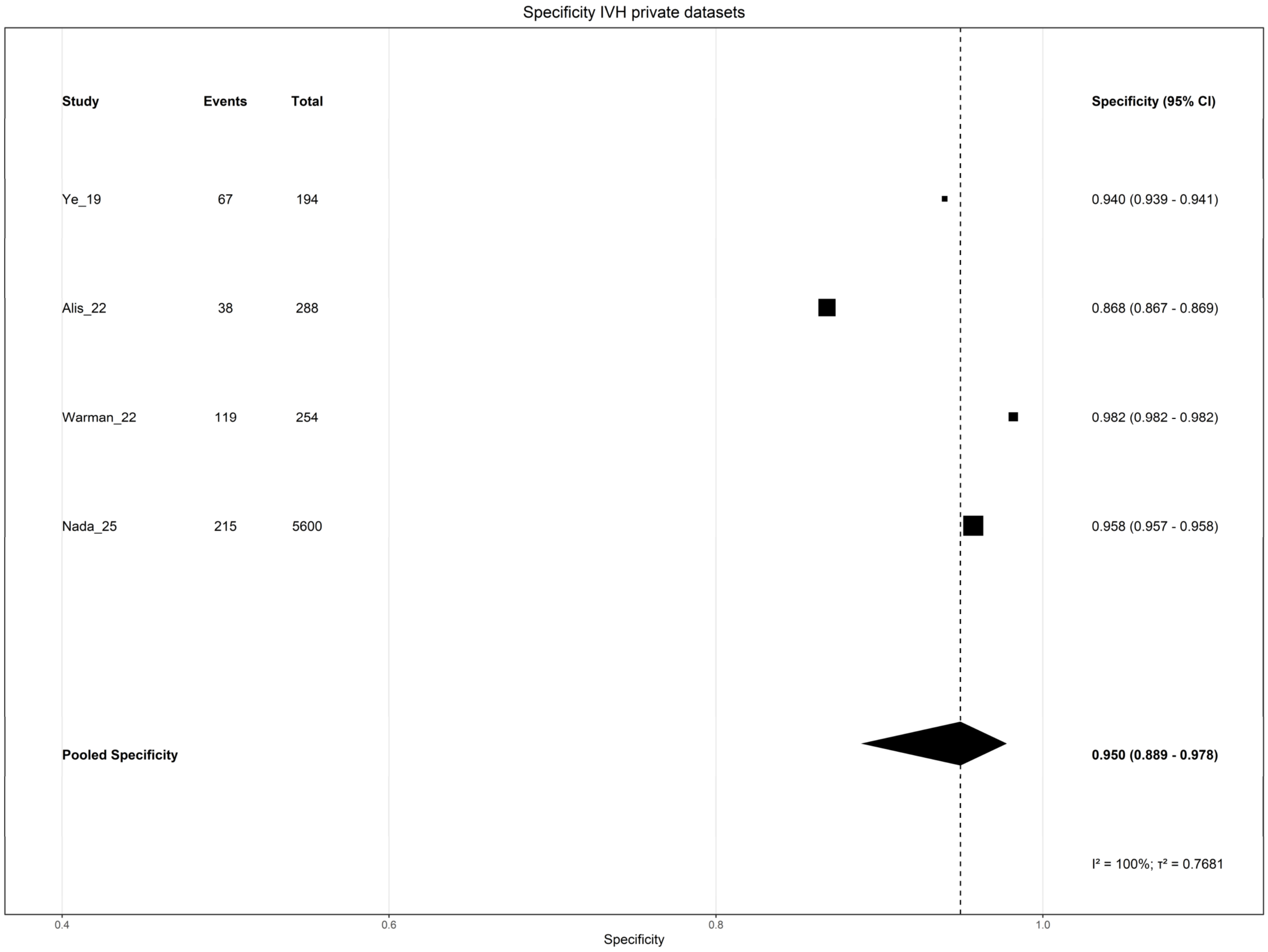

Figure S23. Specificity of IVH studies tested on private datasets.

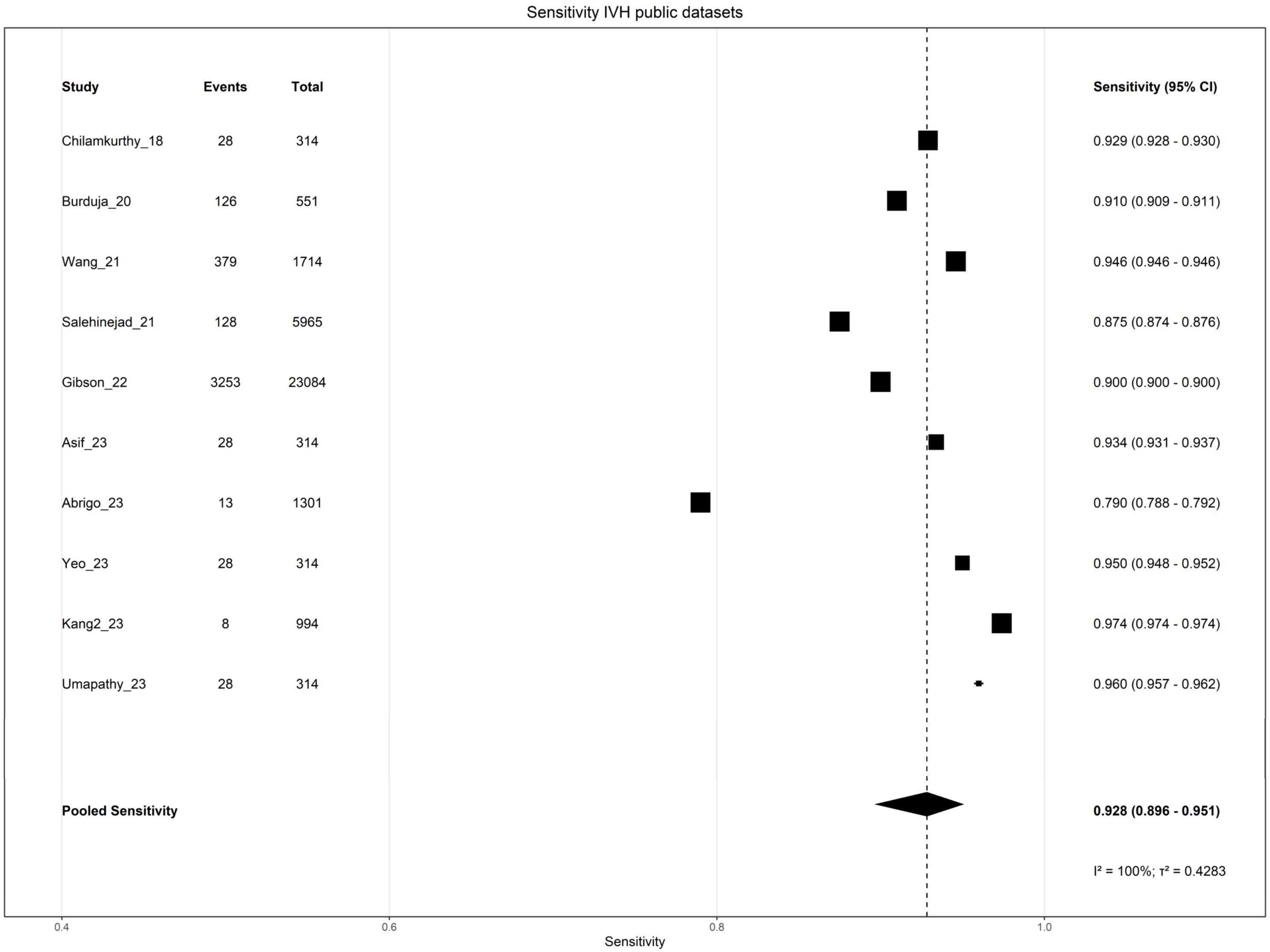

Figure S24. Sensitivity of IVH studies tested on public datasets.

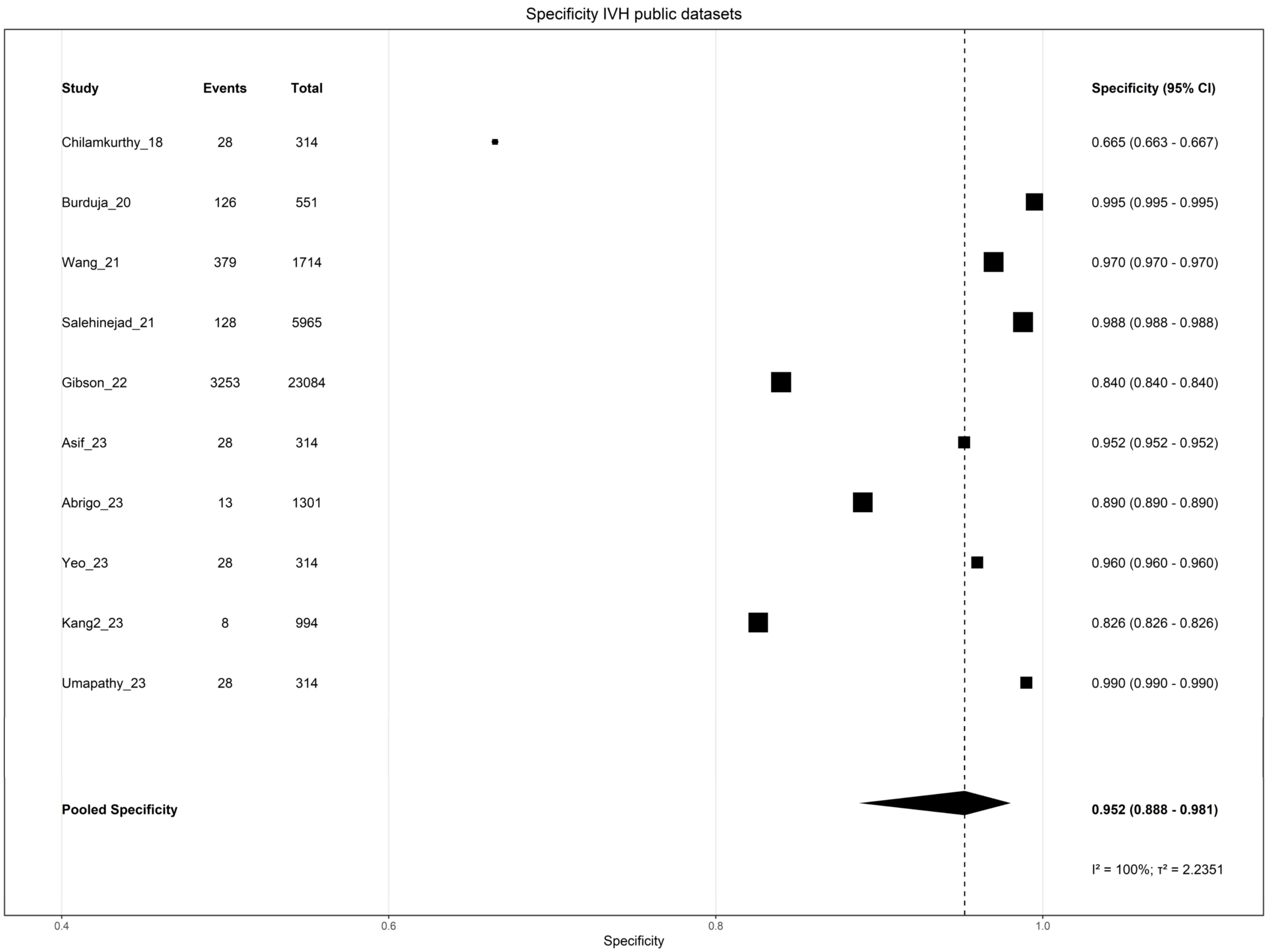

Figure S25. Specificity of IVH studies tested on public datasets.

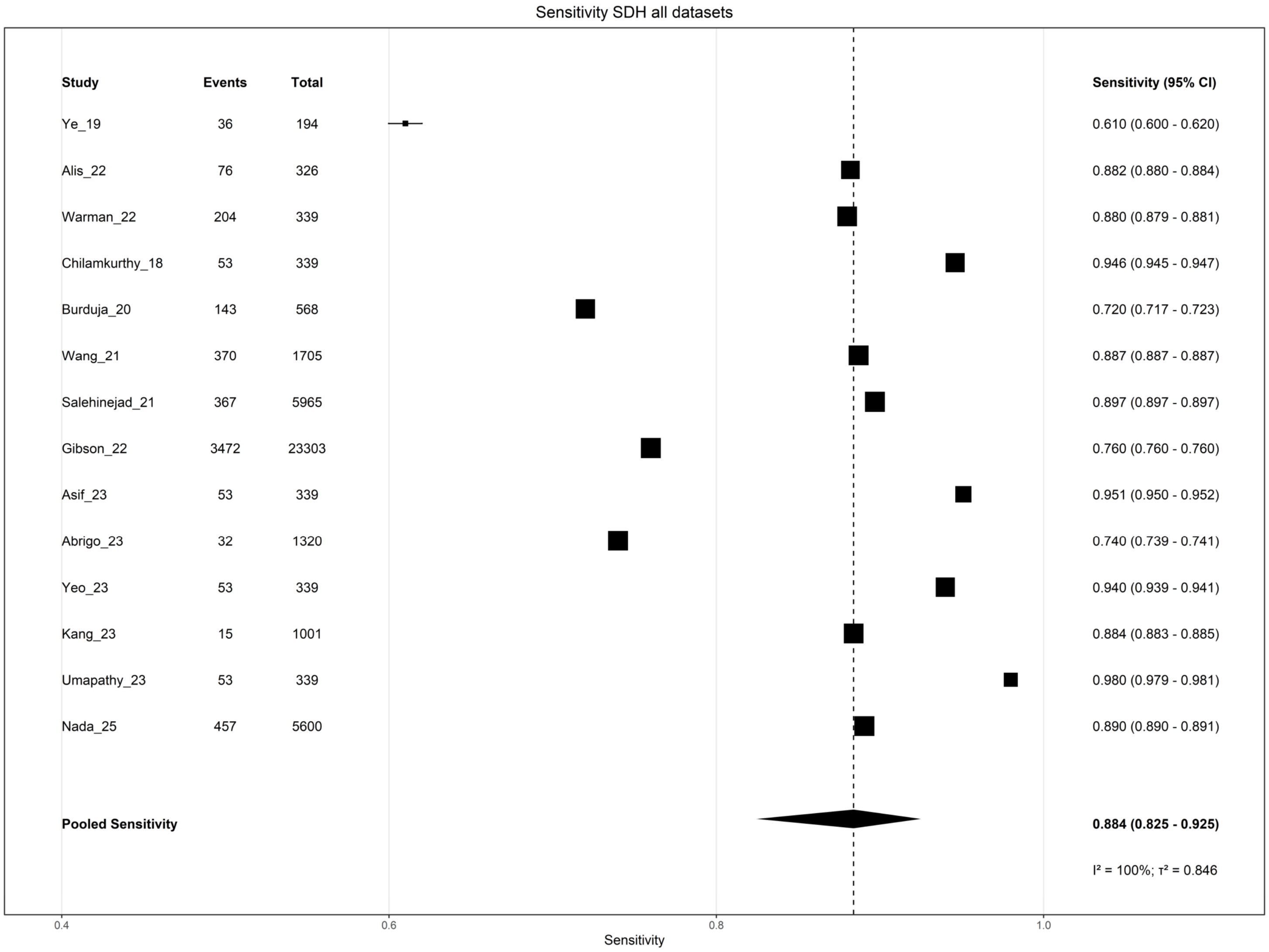

Figure S26. Sensitivity of SDH studies tested on both private and public datasets.

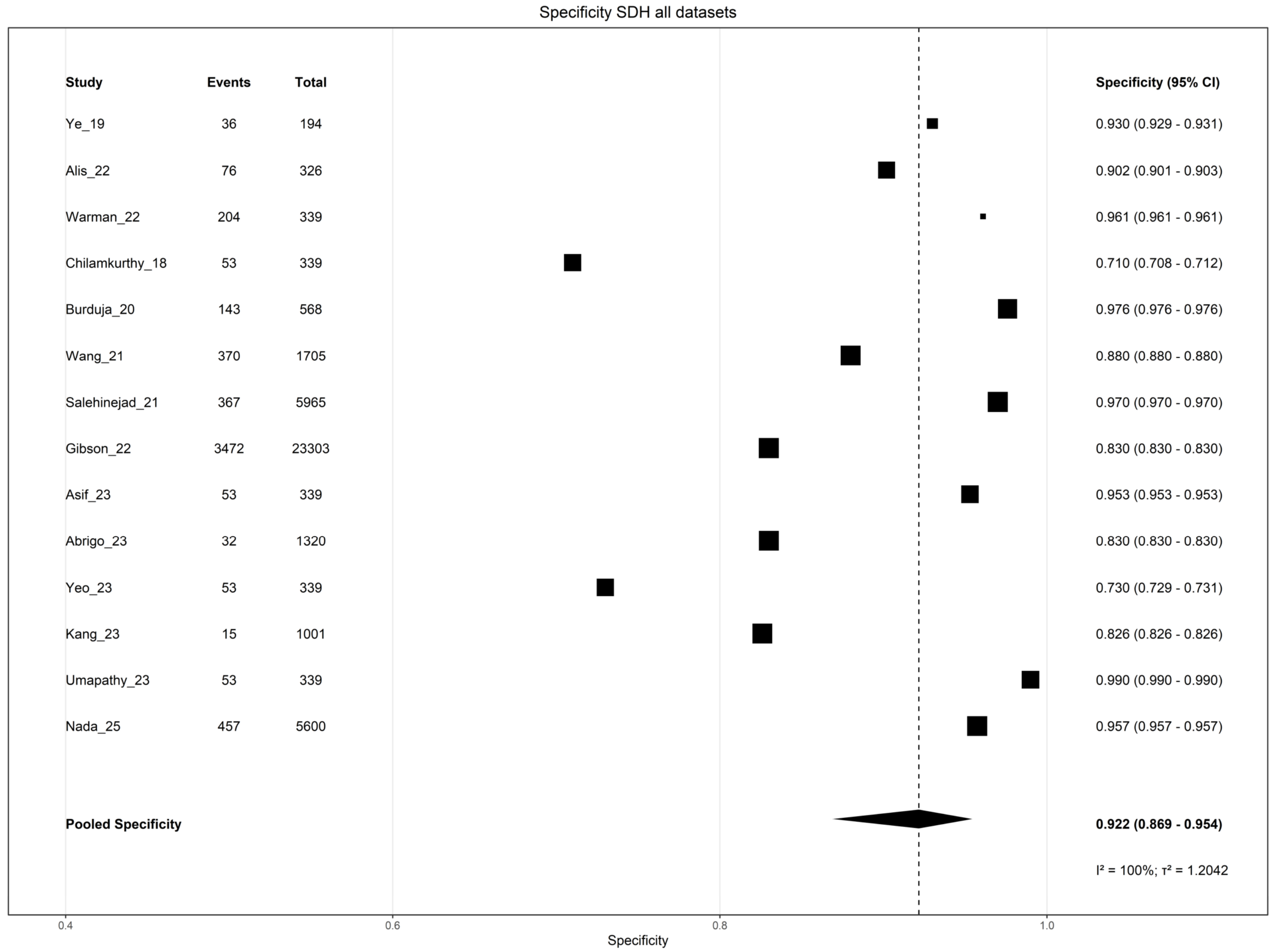

Figure S27. Specificity of SDH studies tested on both private and public datasets.

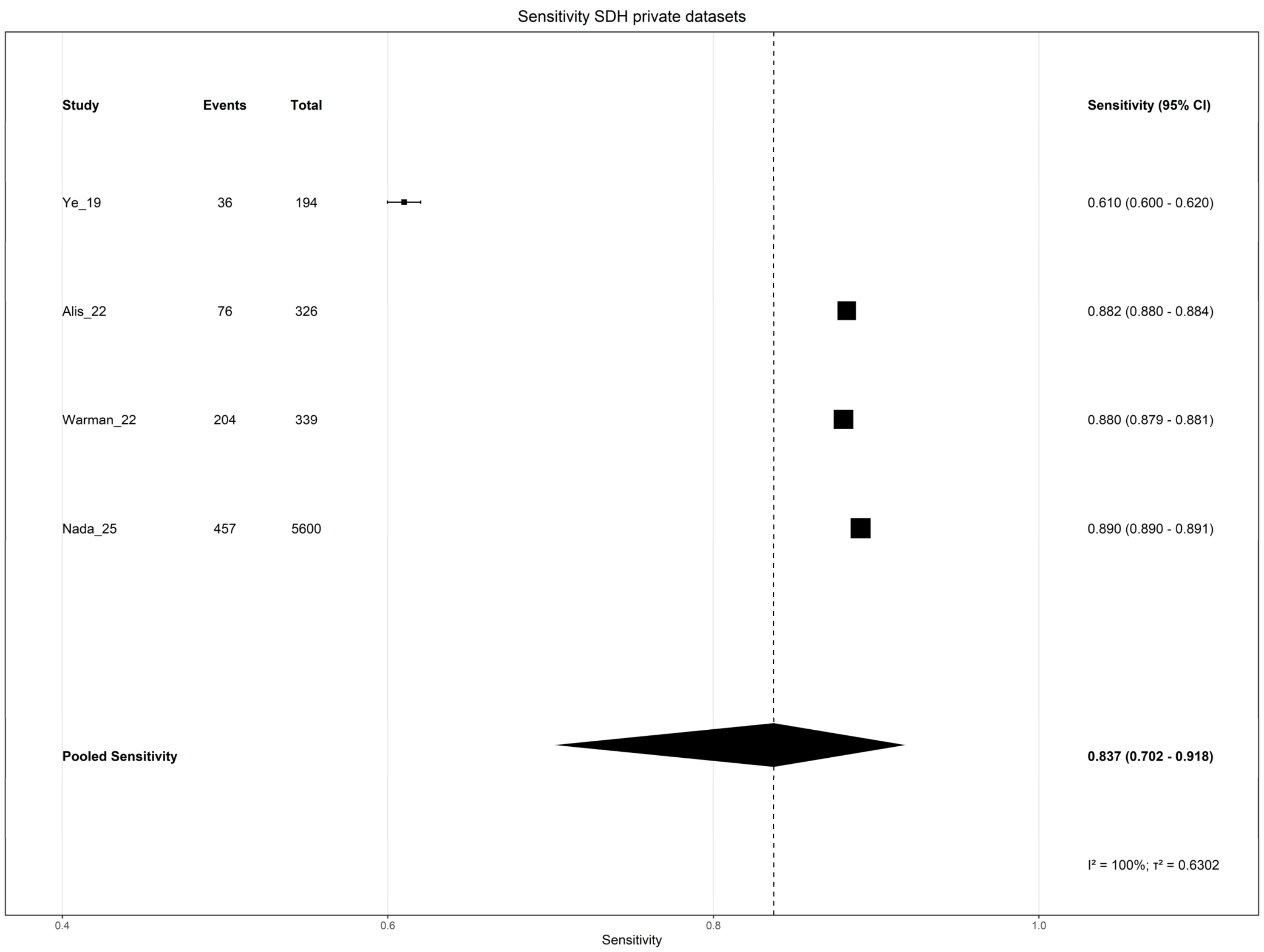

Figure S28. Sensitivity of SDH studies tested on private datasets.

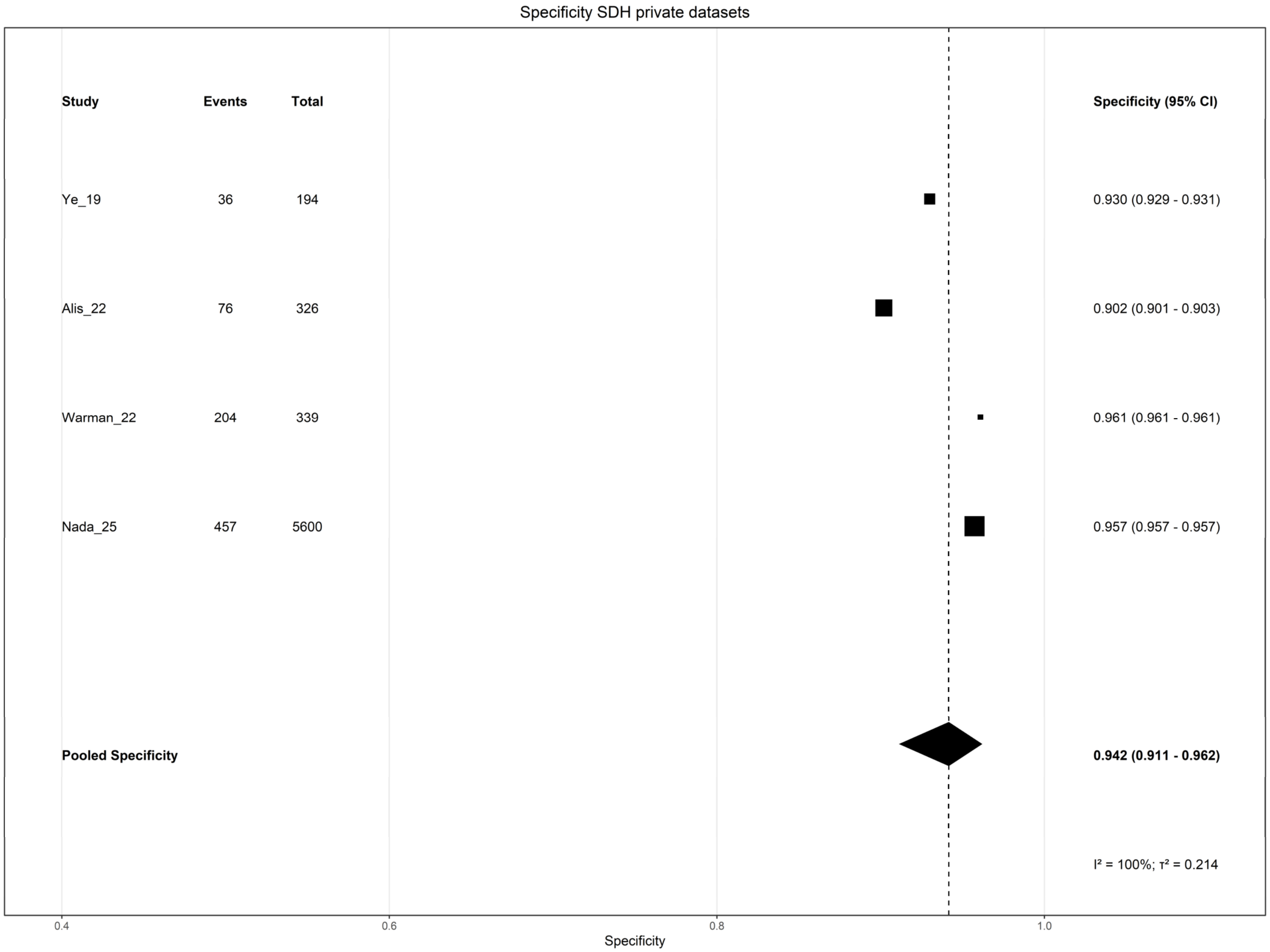

Figure S29. Specificity of SDH studies tested on private datasets.

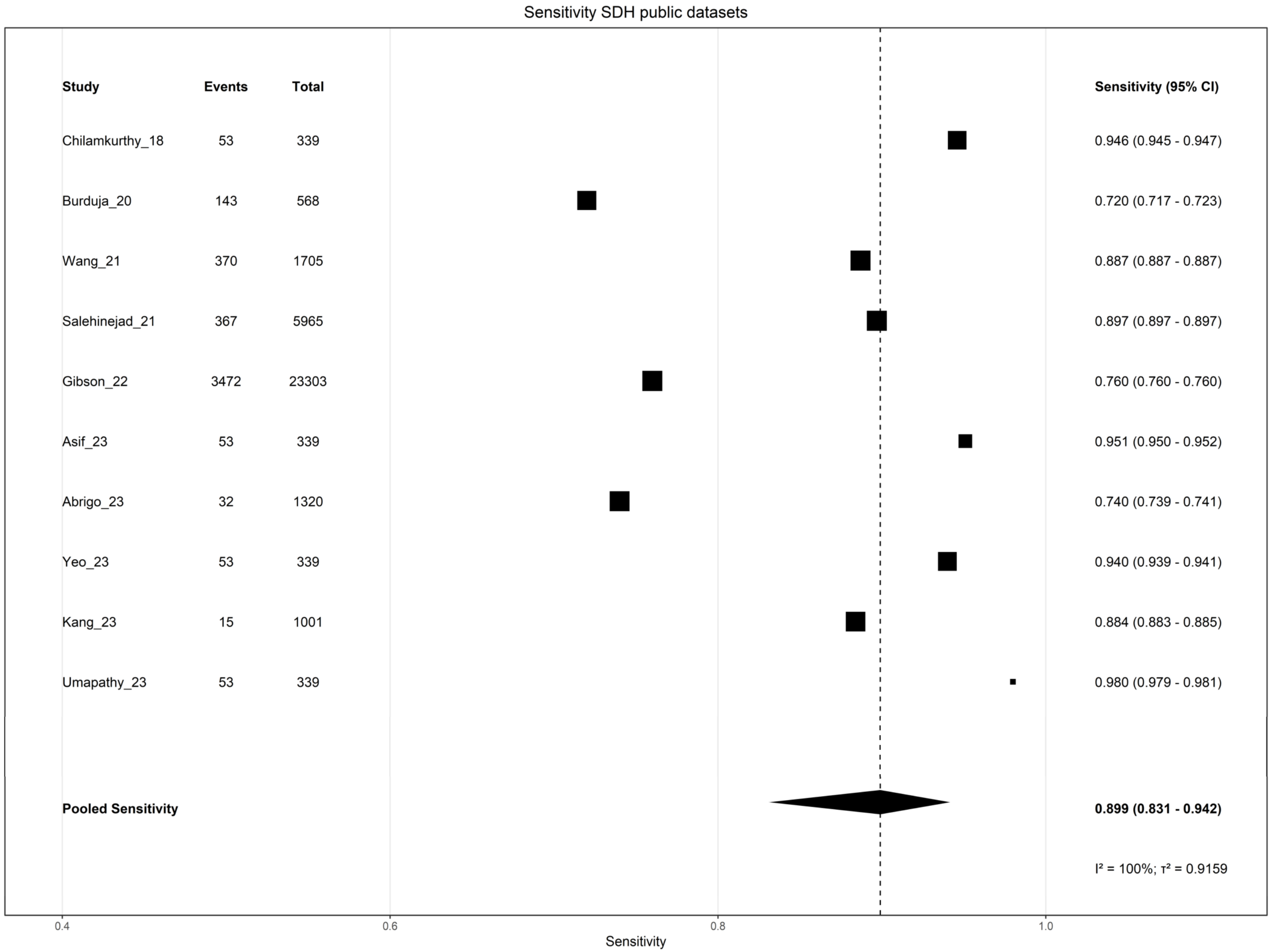

Figure S30. Sensitivity of SDH studies tested on public datasets.

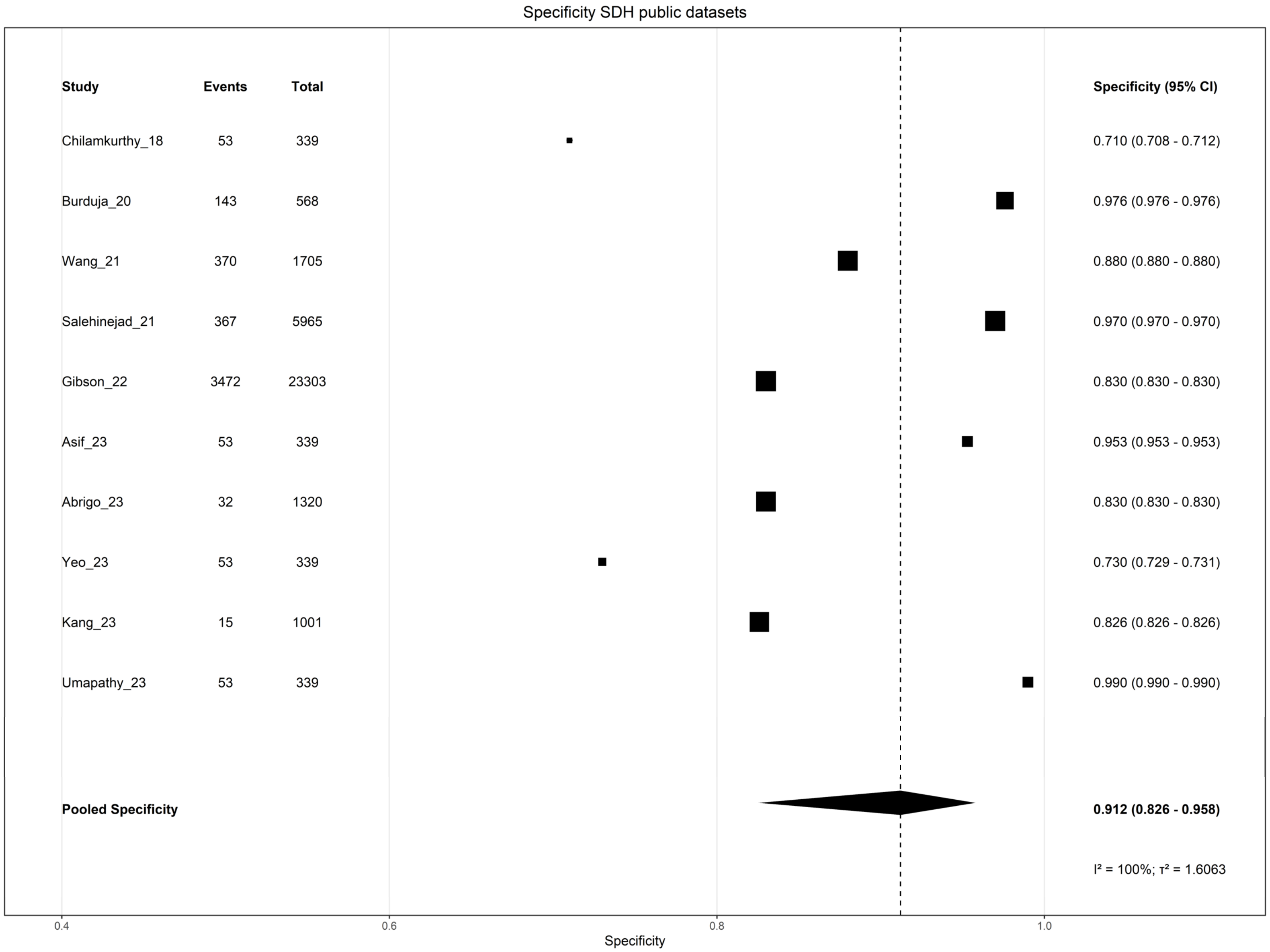

Figure S31. Specificity of SDH studies tested on public datasets.

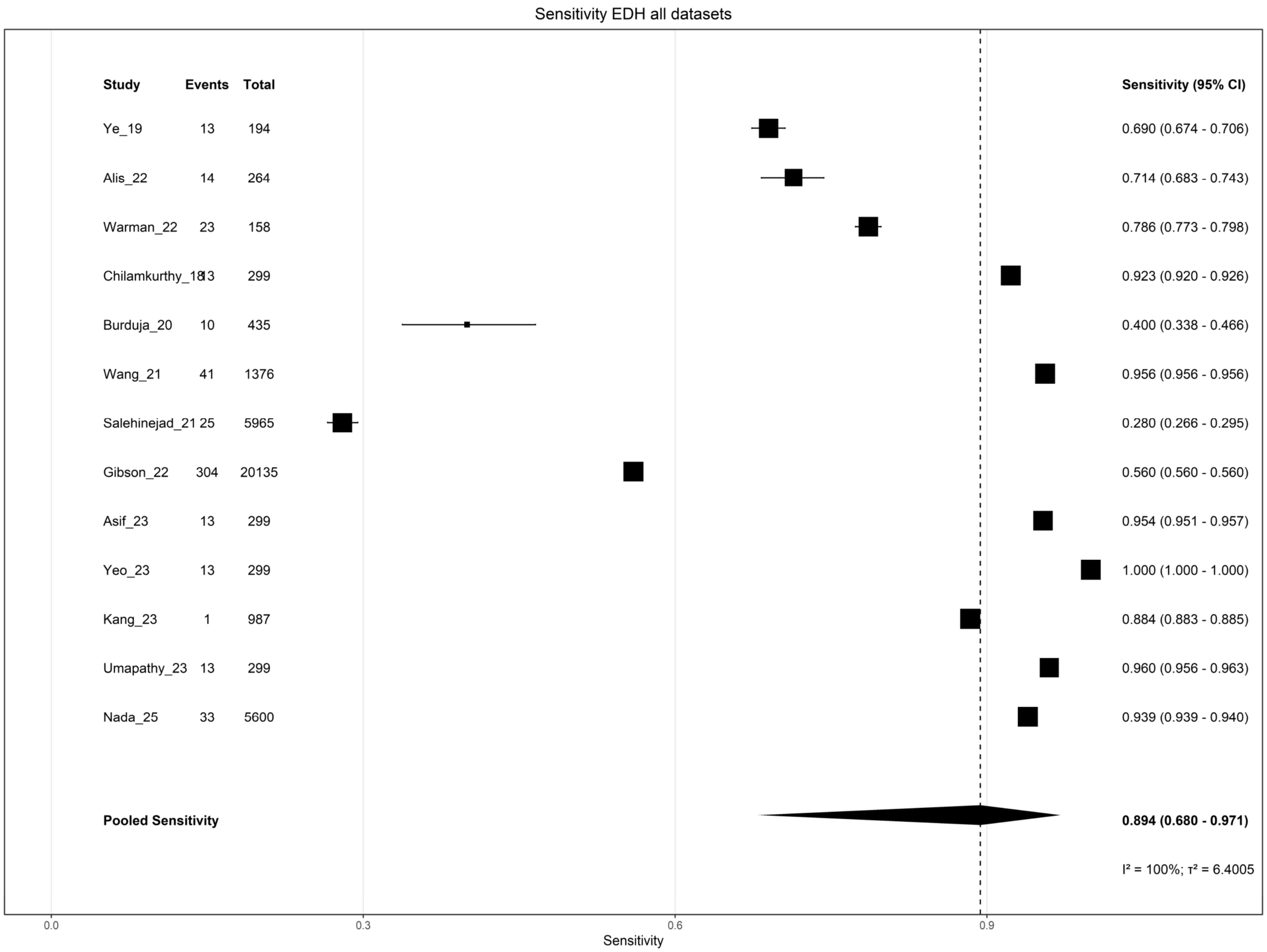

Figure S32. Sensitivity of EDH studies tested on both private and public datasets.

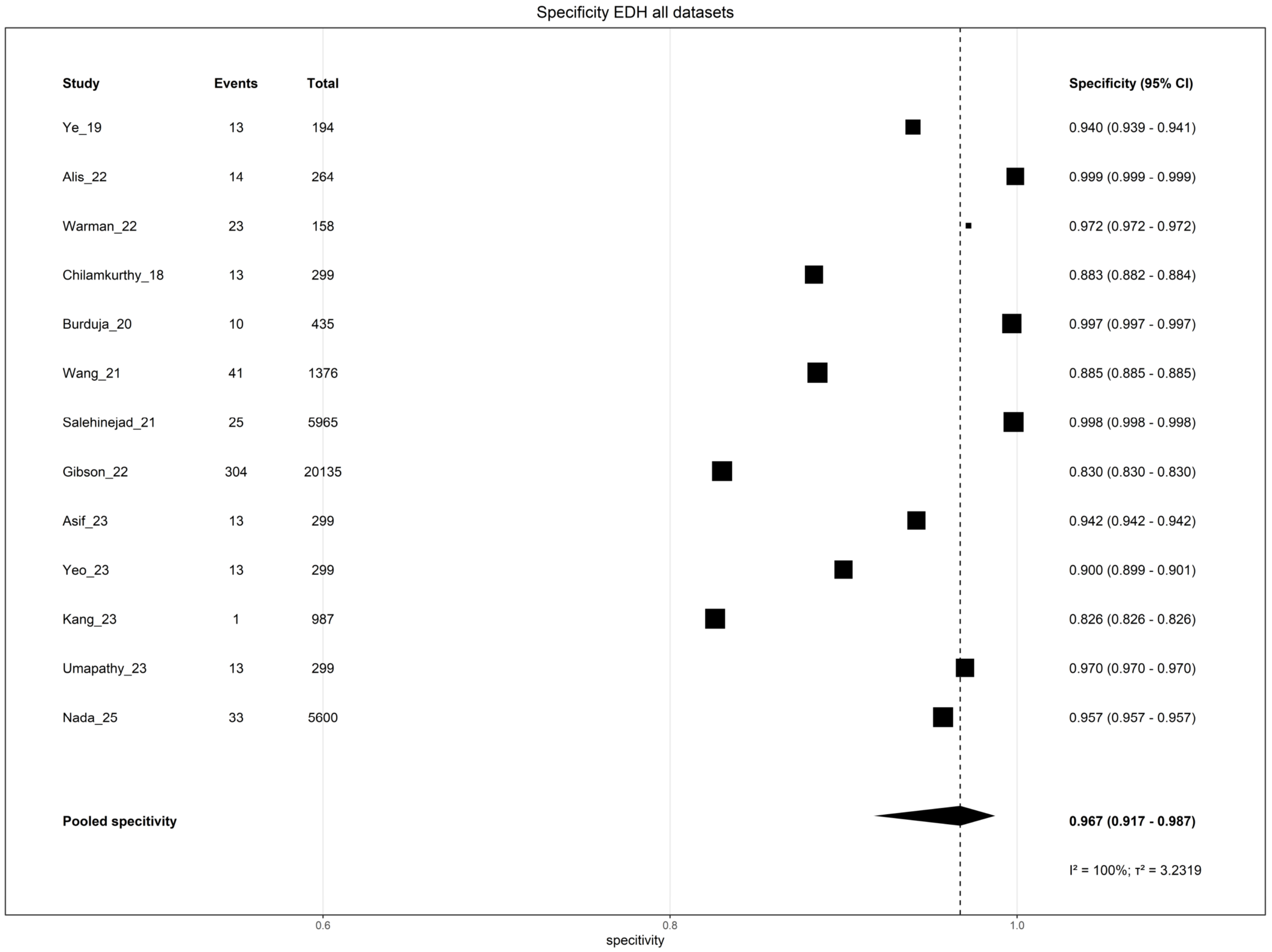

Figure S33. Specificity of EDH studies tested on both private and public datasets.

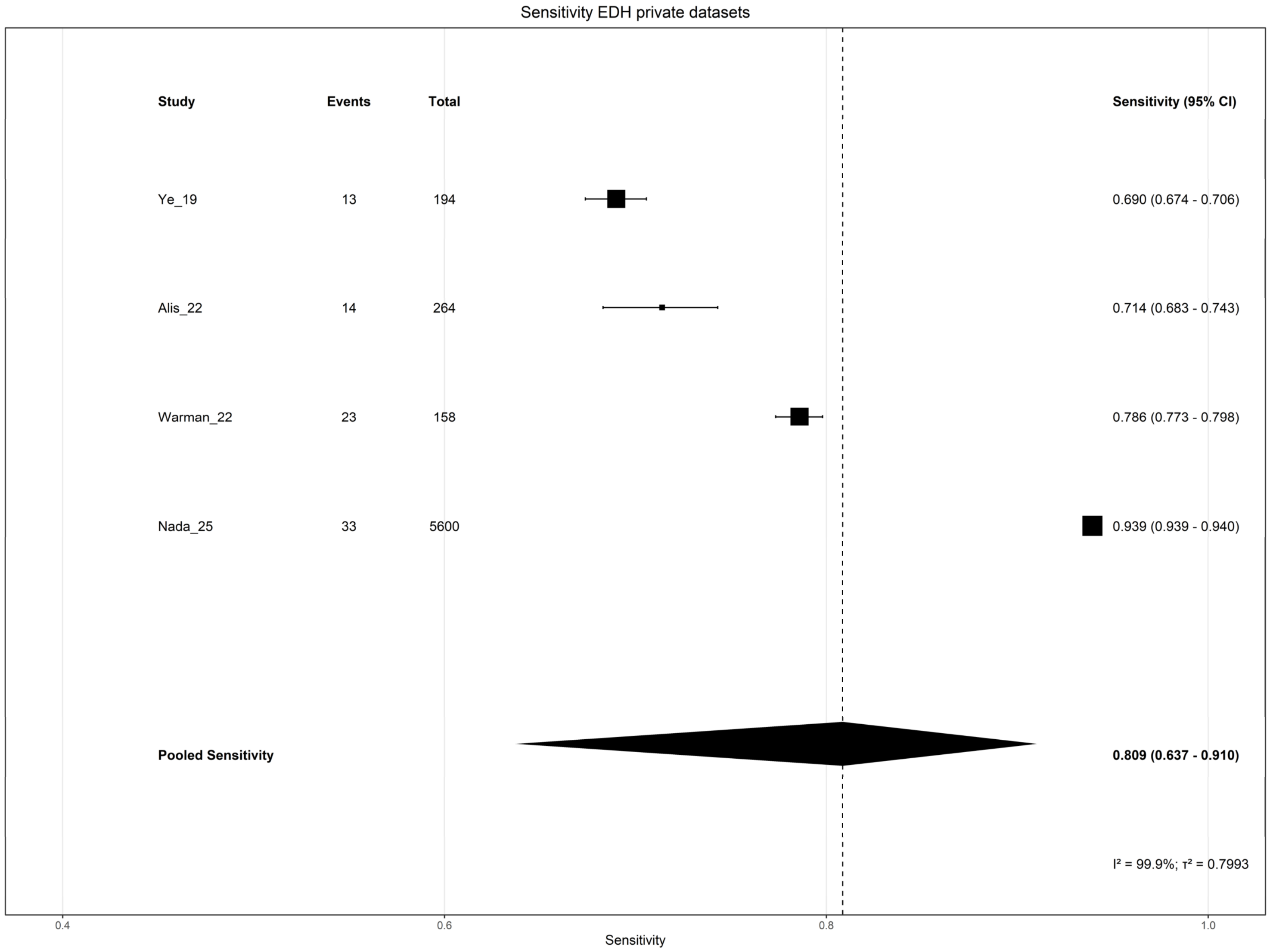

Figure S34. Sensitivity of EDH studies tested on private datasets.

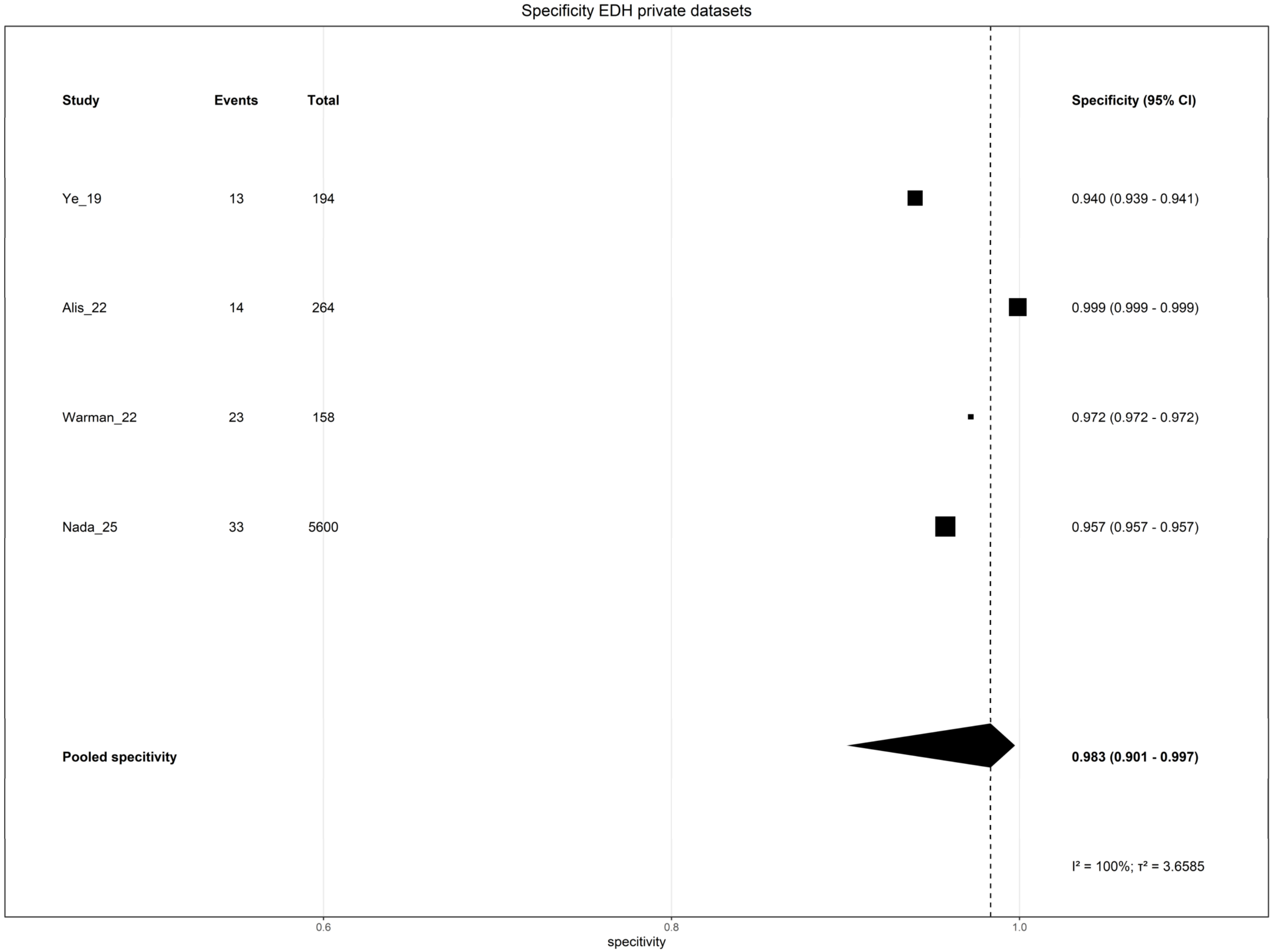

Figure S35. Specificity of EDH studies tested on private datasets.

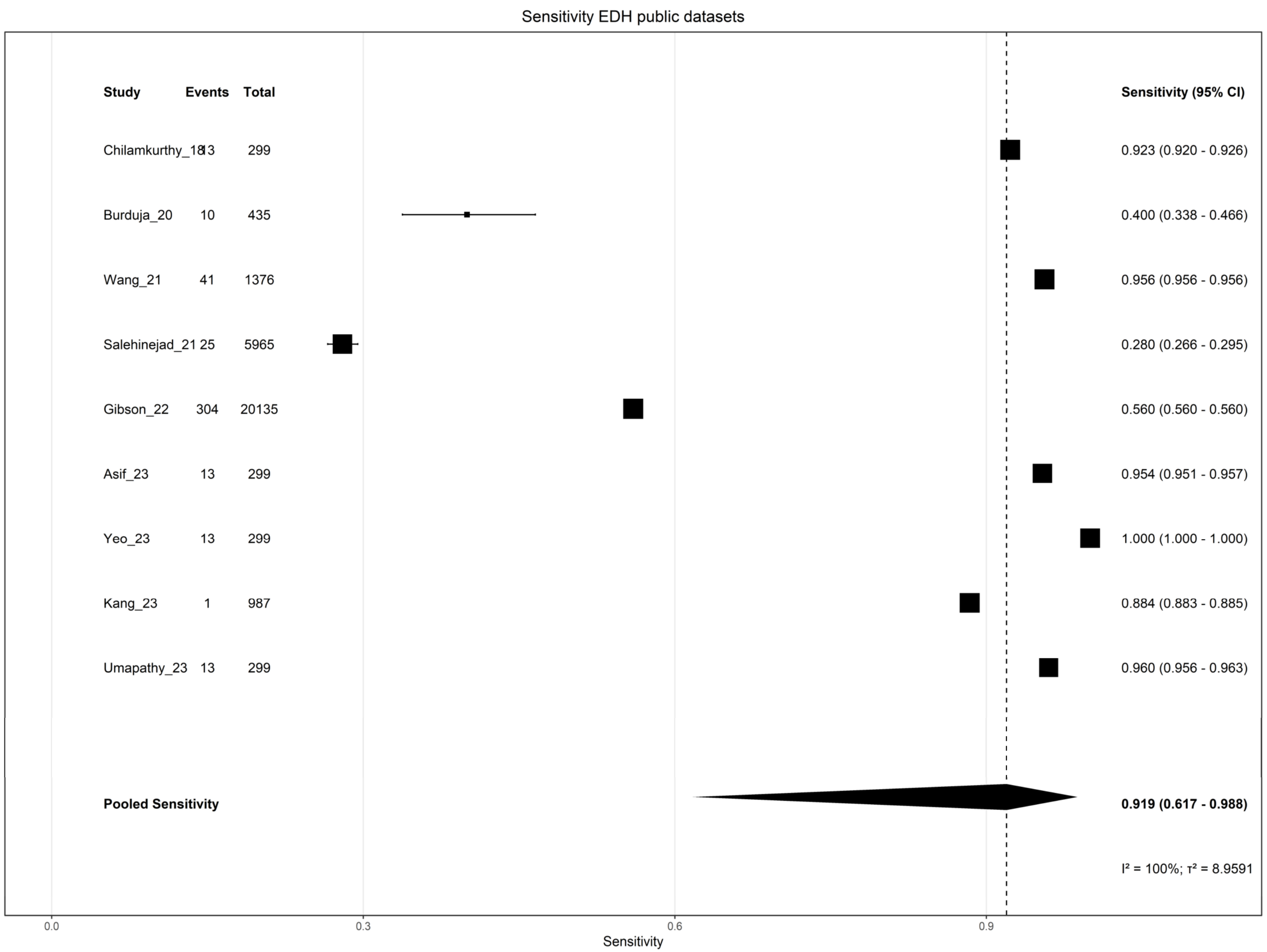

Figure S36. Sensitivity of EDH studies tested on public datasets.

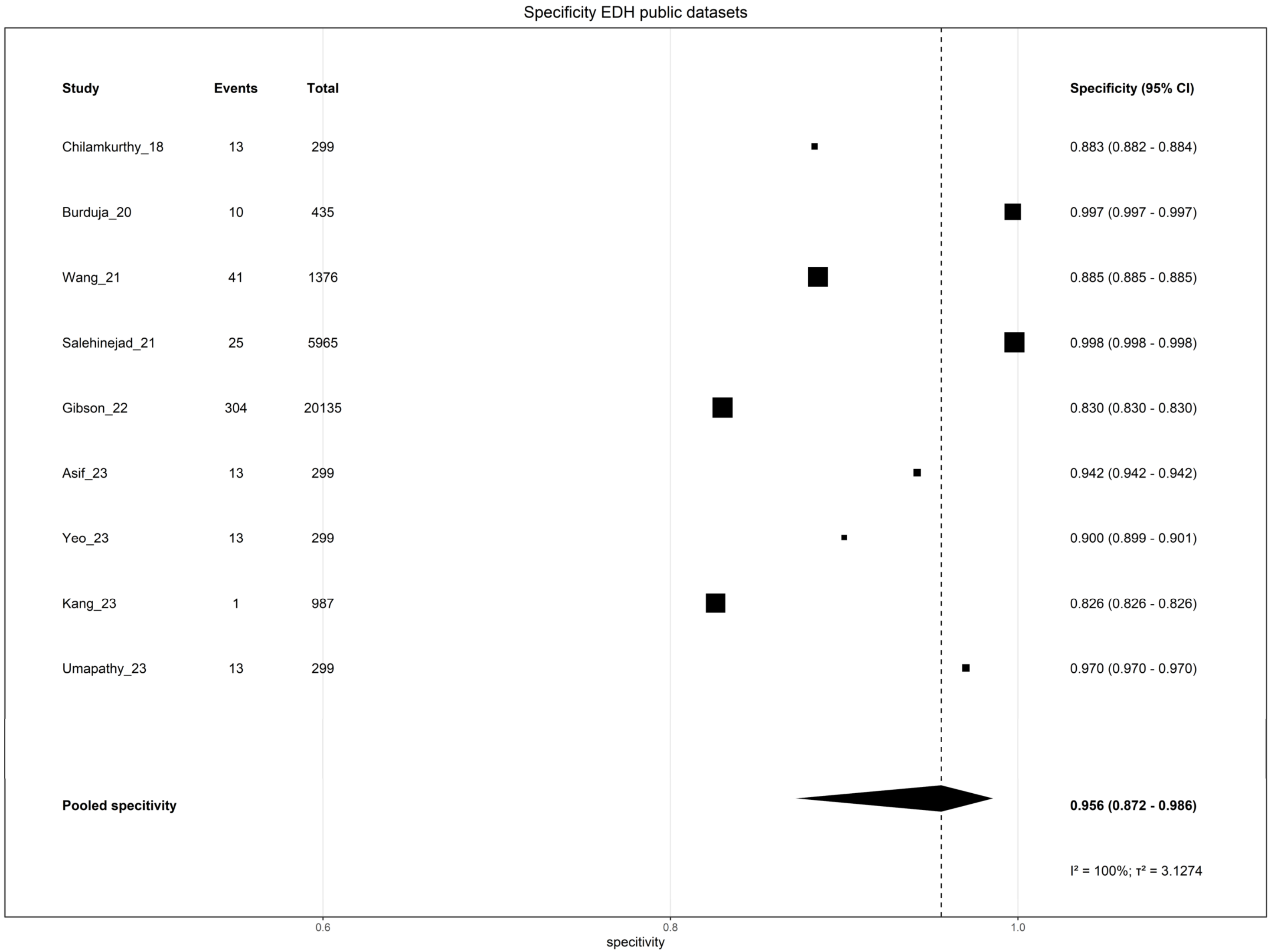

Figure S37. Specificity of EDH studies tested on public datasets.

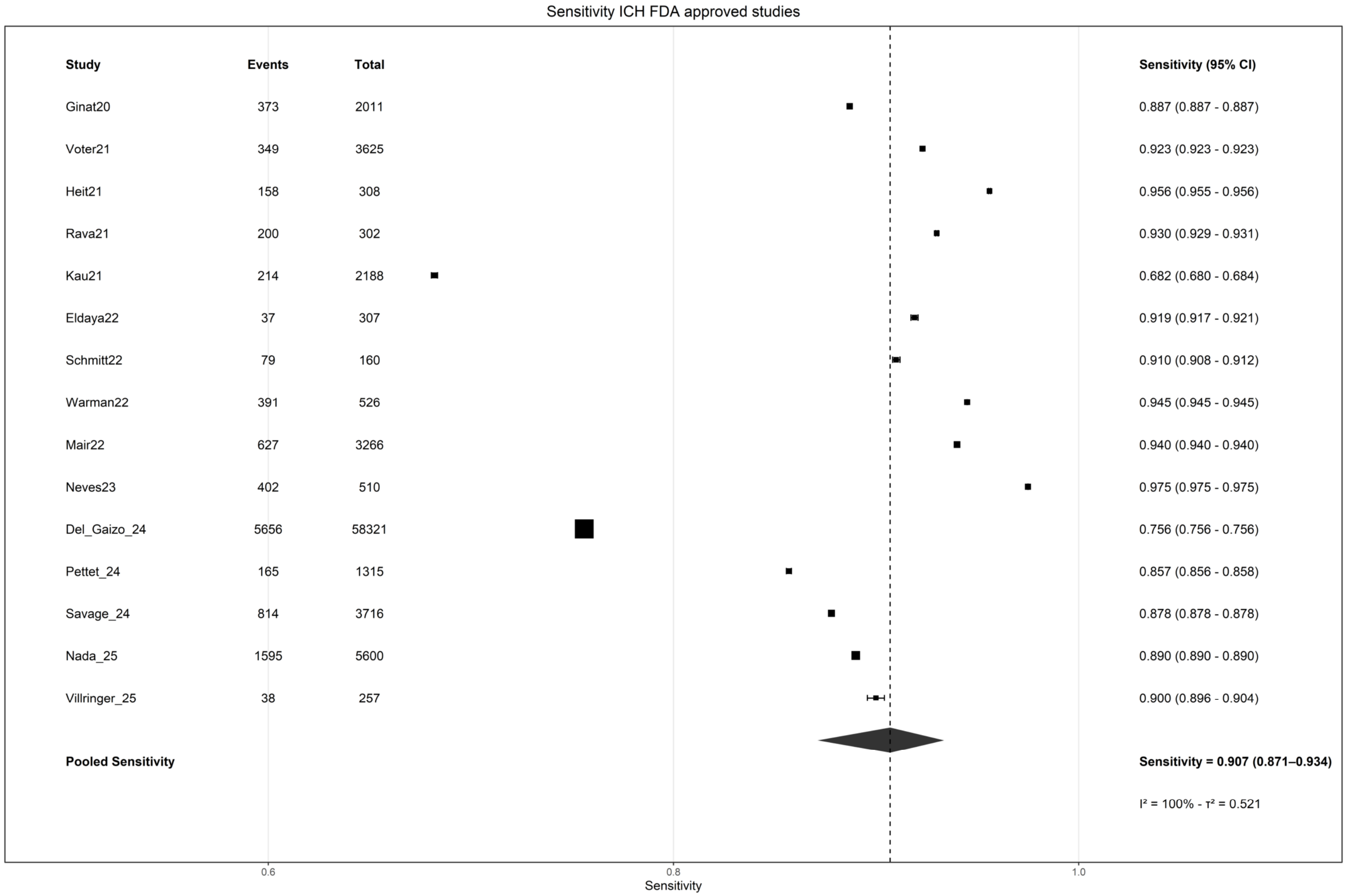

Figure S38. Sensitivity of FDA-approved ICH studies.

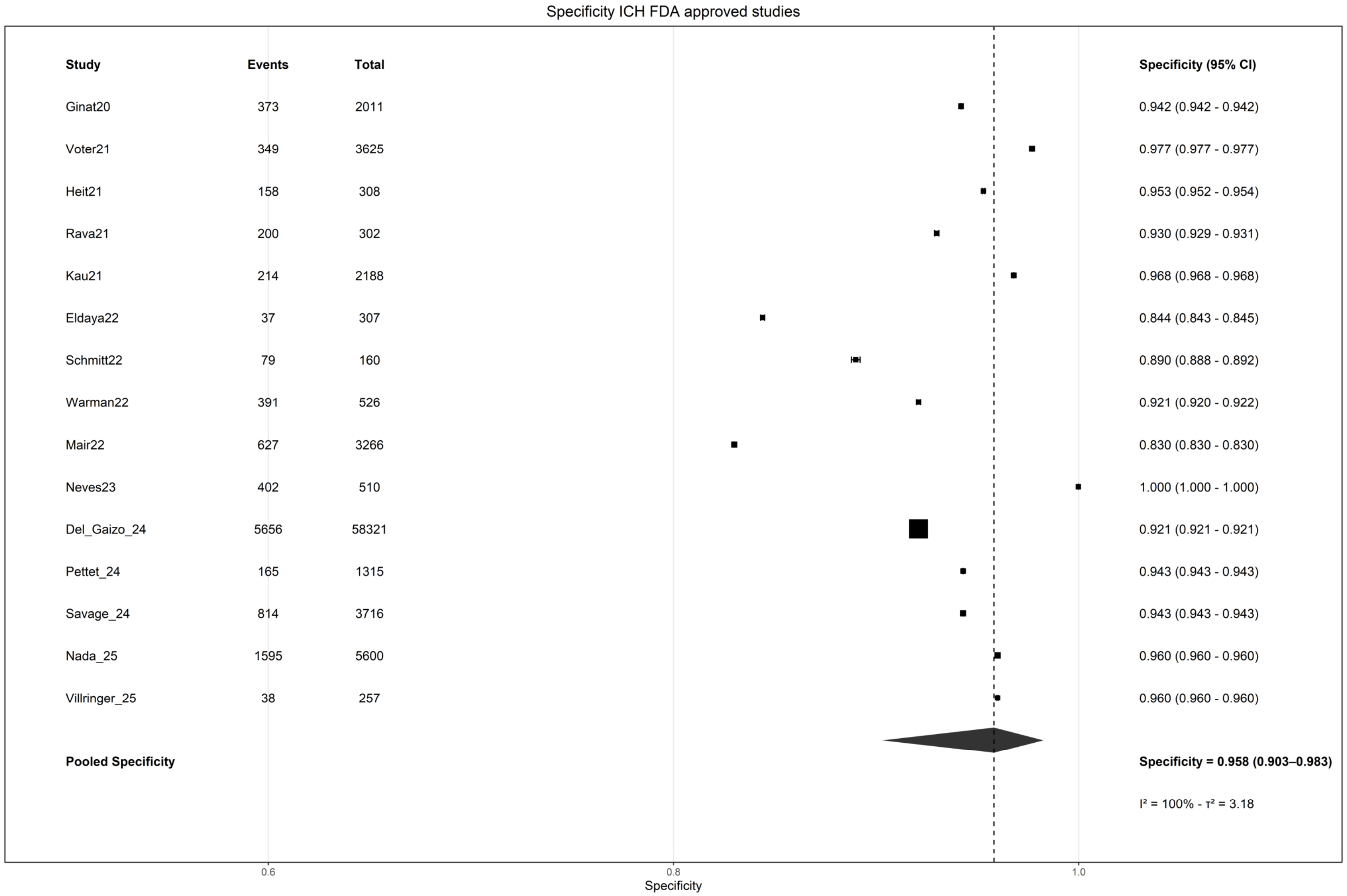

Figure S39. Specificity of FDA-approved ICH studies.

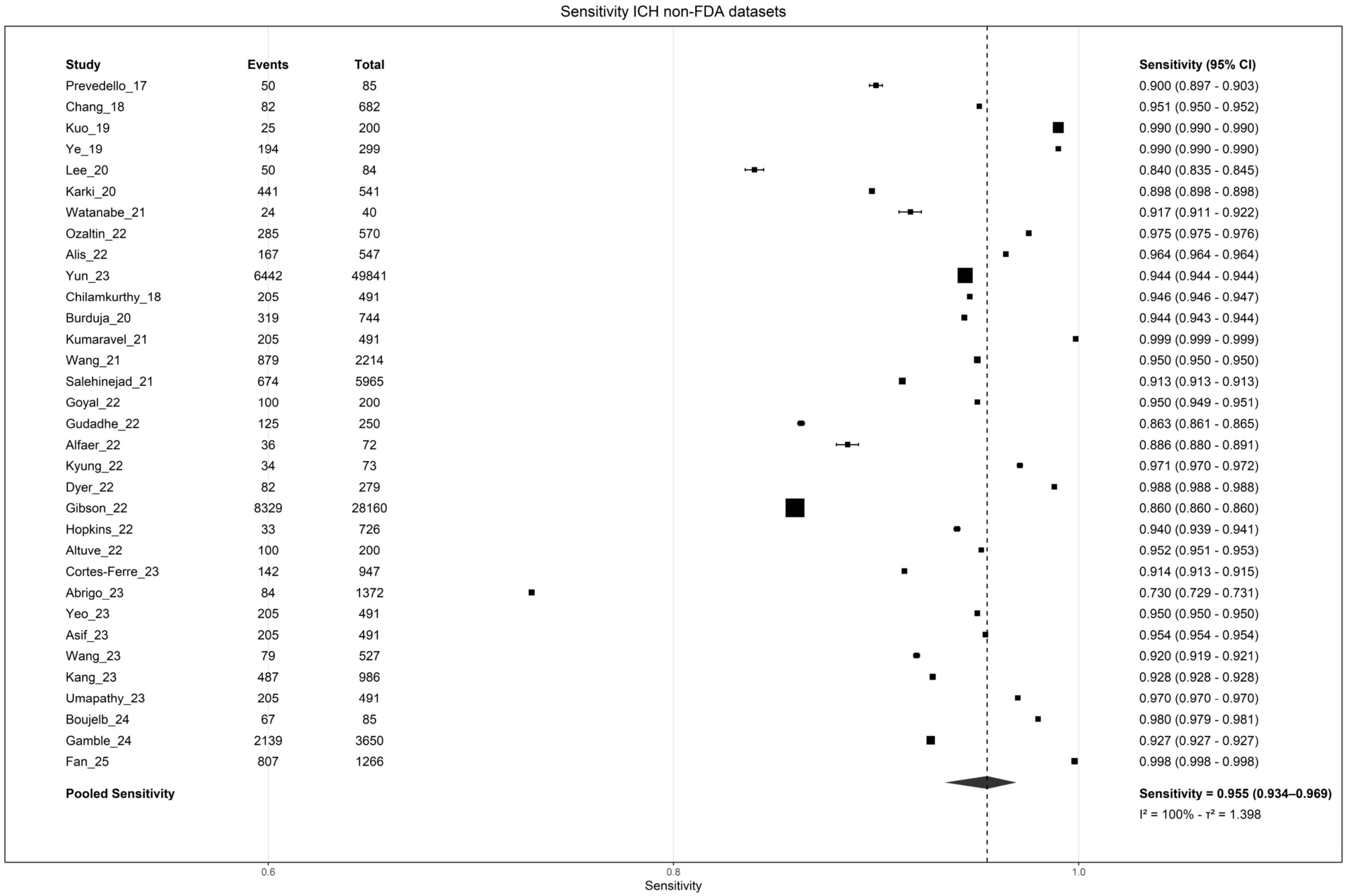

Figure S40. Sensitivity of Non-FDA-approved ICH studies.

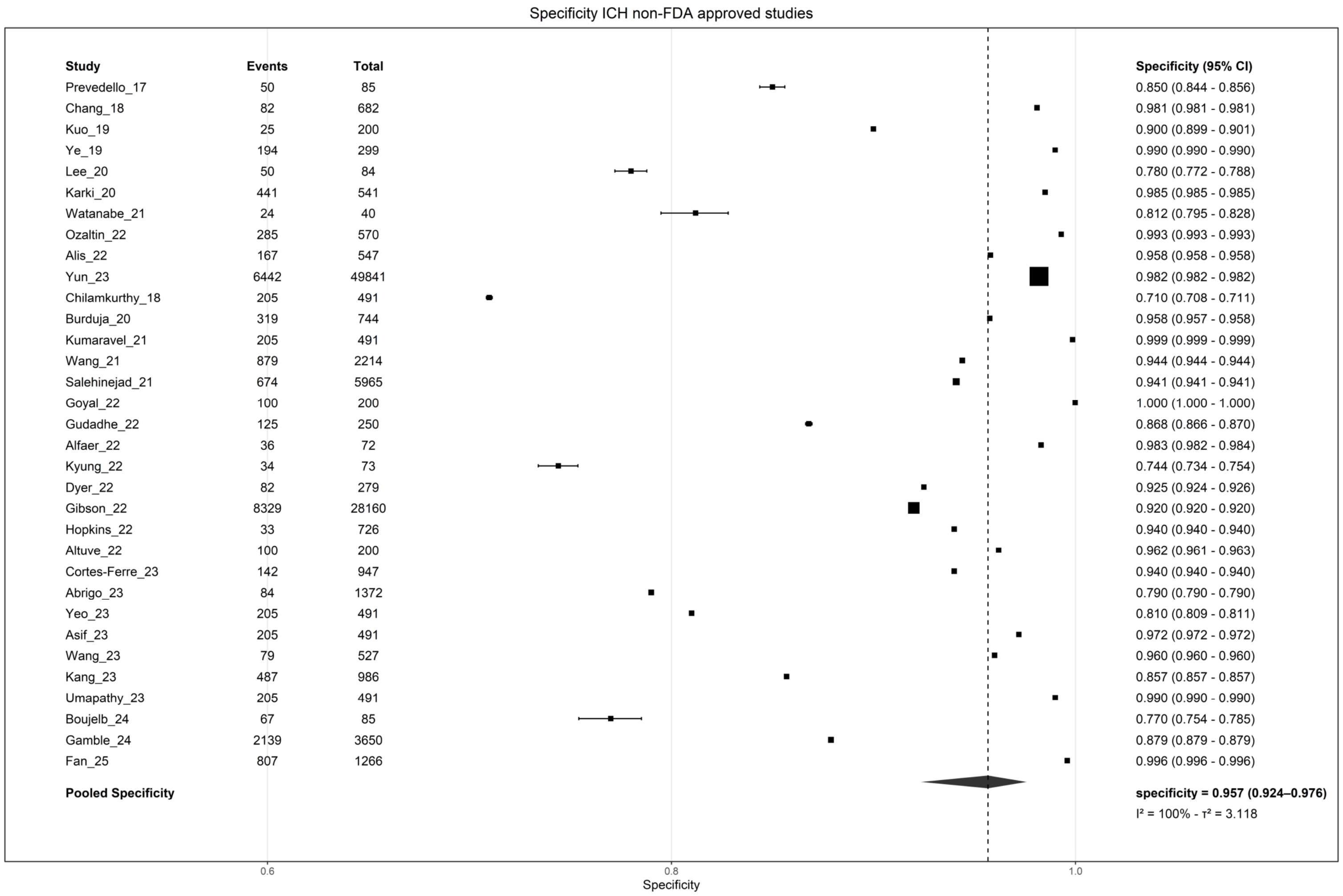

Figure S41. Specificity of Non-FDA-approved ICH studies.

References

1. Prevedello LM, Erdal BS, Ryu JL, Little KJ, Demirer M, Qian S, et al. Automated critical test findings identification and online notification system using artificial intelligence in imaging. Radiology. 2017 Dec 1;285(3):923–31. doi:10.1148/radiol.2017162664 PubMed PMID: 28678669.

2. Chang PD, Kuoy E, Grinband J, Weinberg BD, Thompson M, Homo R, et al. Hybrid 3D/2D convolutional neural network for hemorrhage evaluation on head CT. American Journal of Neuroradiology. 2018 Sep 1;39(9):1609–16. doi:10.3174/ajnr.A5742 PubMed PMID: 30049723.

3. Kuo W, Häne C, Mukherjee P, Malik J, Yuh EL. Expert-level detection of acute intracranial hemorrhage on head computed tomography using deep learning. Proc Natl Acad Sci U S A. 2019 Nov 5;116(45):22737–45. doi:10.1073/pnas.1908021116 PubMed PMID: 31636195.

4. Ye H, Gao F, Yin Y, Guo D, Zhao P, Lu Y, et al. Precise diagnosis of intracranial hemorrhage and subtypes using a three-dimensional joint convolutional and recurrent neural network. Eur Radiol. 2019 Nov 1;29(11):6191–201. doi:10.1007/s00330-019-06163-2 PubMed PMID: 31041565.

5. Ginat DT. Analysis of head CT scans flagged by deep learning software for acute intracranial hemorrhage. Neuroradiology. 2020 Mar 1;62(3):335–40. doi:10.1007/s00234-019-02330-w PubMed PMID: 31828361.

6. Lee JY, Kim JS, Kim TY, Kim YS. Detection and classification of intracranial haemorrhage on CT images using a novel deep-learning algorithm. Sci Rep. 2020 Dec 1;10(1). doi:10.1038/s41598-020-77441-z PubMed PMID: 33239711.

7. Karki M, Cho J, Lee E, Hahm MH, Yoon SY, Kim M, et al. CT window trainable neural network for improving intracranial hemorrhage detection by combining multiple settings. Artif Intell Med. 2020 Jun 1;106. doi:10.1016/j.artmed.2020.101850 PubMed PMID: 32593388.

8. Heit JJ, Coelho H, Lima FO, Granja M, Aghaebrahim A, Hanel R, et al. Automated cerebral hemorrhage detection using rapid. American Journal of Neuroradiology. 2021 Feb 1;42(2):273–8. doi:10.3174/ajnr.A6926 PubMed PMID: 33361378.

9. Watanabe Y, Tanaka T, Nishida A, Takahashi H, Fujiwara M, Fujiwara T, et al. Improvement of the diagnostic accuracy for intracranial haemorrhage using deep learning-based computer-assisted detection [Internet]. doi:10.1007/s00234-020-02566-x/Published

10. Rava RA, Seymour SE, LaQue ME, Peterson BA, Snyder K V., Mokin M, et al. Assessment of an Artificial Intelligence Algorithm for Detection of Intracranial Hemorrhage. World Neurosurg. 2021 Jun 1;150:e209–17. doi:10.1016/j.wneu.2021.02.134 PubMed PMID: 33684578.

11. Voter AF, Meram E, Garrett JW, Yu JPJ. Diagnostic Accuracy and Failure Mode Analysis of a Deep Learning Algorithm for the Detection of Intracranial Hemorrhage. Journal of the American College of Radiology. 2021 Aug 1;18(8):1143–52. doi:10.1016/j.jacr.2021.03.005 PubMed PMID: 33819478.

12. Alis D, Alis C, Yergin M, Topel C, Asmakutlu O, Bagcilar O, et al. A joint convolutional-recurrent neural network with an attention mechanism for detecting intracranial hemorrhage on noncontrast head CT. Sci Rep. 2022 Dec 1;12(1). doi:10.1038/s41598-022-05872-x PubMed PMID: 35136123.

13. Eldaya RW, Kansagra AP, Zei M, Mason E, Holder D, Heitsch L, et al. Performance of Automated RAPID Intracranial Hemorrhage Detection in Real-World Practice: A Single-Institution Experience. J Comput Assist Tomogr. 2022 Sep 1;46(5):770–4. doi:10.1097/RCT.0000000000001335 PubMed PMID: 35617649.

14. Schmitt N, Mokli Y, Weyland CS, Gerry S, Herweh C, Ringleb PA, et al. Automated detection and segmentation of intracranial hemorrhage suspect hyperdensities in non-contrast-enhanced CT scans of acute stroke patients. Eur Radiol. 2022 Apr 1;32(4):2246–54. doi:10.1007/s00330-021-08352-4 PubMed PMID: 34773465.

15. Mair G, White P, Bath PM, Muir KW, Al-Shahi Salman R, Martin C, et al. External Validation of e-ASPECTS Software for Interpreting Brain CT in Stroke. Ann Neurol. 2022 Dec 1;92(6):943–57. doi:10.1002/ana.26495 PubMed PMID: 36053916.

16. Kau T, Ziurlys M, Taschwer M, Kloss-Brandstätter A, Grabner G, Deutschmann H. FDA-approved deep learning software application versus radiologists with different levels of expertise: detection of intracranial hemorrhage in a retrospective single-center study. Neuroradiology. 2022 May 1;64(5):981–90. doi:10.1007/s00234-021-02874-w PubMed PMID: 34988593.

17. Warman R, Warman A, Warman P, Degnan A, Blickman J, Chowdhary V, et al. Deep Learning System Boosts Radiologist Detection of Intracranial Hemorrhage. Cureus. 2022 Oct 13. doi:10.7759/cureus.30264

18. Ozaltin O, Coskun O, Yeniay O, Subasi A. A Deep Learning Approach for Detecting Stroke from Brain CT Images Using OzNet. Bioengineering. 2022 Dec 1;9(12). doi:10.3390/bioengineering9120783

19. Wang D, Jin R, Shieh CC, Ng AY, Pham H, Dugal T, et al. Real world validation of an AI-based CT hemorrhage detection tool. Front Neurol. 2023;14. doi:10.3389/fneur.2023.1177723

20. Yun TJ, Choi JW, Han M, Jung WS, Choi SH, Yoo RE, et al. Deep learning based automatic detection algorithm for acute intracranial haemorrhage: a pivotal randomized clinical trial. NPJ Digit Med. 2023 Dec 1;6(1). doi:10.1038/s41746-023-00798-8

21. Neves G, Warman PI, Warman A, Warman R, Bueso T, Vadhan JD, et al. External Validation of an Artificial Intelligence Device for Intracranial Hemorrhage Detection. World Neurosurg. 2023 May 1;173:e800–7. doi:10.1016/j.wneu.2023.03.019 PubMed PMID: 36906085.

22. Boujelb Z, Idrissi A, Benba A, Chakir EM. Detecting hemorrhagic stroke from computed tomographic scans using machine learning models comparison. Data and Metadata. 2024 Jan 1;3. doi:10.56294/dm2024.548

23. Del Gaizo AJ, Osborne TF, Shahoumian T, Sherrier R. Deep Learning to Detect Intracranial Hemorrhage in a National Teleradiology Program and the Impact on Interpretation Time. Radiol Artif Intell. 2024 Sep 1;6(5). doi:10.1148/ryai.240067

24. Pettet G, West J, Robert D, Khetani A, Kumar S, Golla S, et al. A retrospective audit of an artificial intelligence software for the detection of intracranial haemorrhage used by a teleradiology company in the United Kingdom. BJR Open. 2024 Jan 1;6(1). doi:10.1093/bjro/tzae033 PubMed PMID: 39479271.

25. Savage CH, Tanwar M, Elkassem AA, Sturdivant A, Hamki O, Sotoudeh H, et al. Prospective Evaluation of Artificial Intelligence Triage of Intracranial Hemorrhage on Noncontrast Head CT Examinations. American Journal of Roentgenology. 2024 Nov 1;223(5). doi:10.2214/AJR.24.31639 PubMed PMID: 39230402.

26. Fan J, Fan X, Song C, Wang X, Feng B, Li L, et al. Dual-task vision transformer for rapid and accurate intracerebral hemorrhage CT image classification. Sci Rep. 2024 Dec 1;14(1). doi:10.1038/s41598-024-79090-y PubMed PMID: 39572600.

27. Nada A, Sayed AA, Hamouda M, Tantawi M, Khan A, Alt A, et al. External validation and performance analysis of a deep learning-based model for the detection of intracranial hemorrhage. Neuroradiology Journal. 2025 Jun 1;38(3):312–21. doi:10.1177/19714009241303078 PubMed PMID: 39601611.

28. Villringer K, Sokiranski R, Opfer R, Spies L, Hamann M, Bormann A, et al. An Artificial Intelligence Algorithm Integrated into the Clinical Workflow Can Ensure High Quality Acute Intracranial Hemorrhage CT Diagnostic. Clin Neuroradiol. 2025 Mar 1;35(1):115–22. doi:10.1007/s00062-024-01461-9 PubMed PMID: 39325081.

29. Chilamkurthy S, Ghosh R, Tanamala S, Biviji M, Campeau NG, Venugopal VK, et al. Deep learning algorithms for detection of critical findings in head CT scans: a retrospective study. The Lancet. 2018 Dec 1;392(10162):2388–96. doi:10.1016/S0140-6736(18)31645-3 PubMed PMID: 30318264.

30. Burduja M, Ionescu RT, Verga N. Accurate and efficient intracranial hemorrhage detection and subtype classification in 3D CT scans with convolutional and long short-term memory neural networks. Sensors (Switzerland). 2020 Oct 1;20(19):1–21. doi:10.3390/s20195611 PubMed PMID: 33019508.

31. Kumaravel P, Mohan S, Arivudaiyanambi J, Shajil N, Venkatakrishnan HN. A Simplified Framework for the Detection of Intracranial Hemorrhage in CT Brain Images Using Deep Learning. Current Medical Imaging Formerly Current Medical Imaging Reviews. 2021 Feb 19;17(10):1226–36. doi:10.2174/1573405617666210218100641 PubMed PMID: 33602101.

32. Wang X, Shen T, Yang S, Lan J, Xu Y, Wang M, et al. A deep learning algorithm for automatic detection and classification of acute intracranial hemorrhages in head CT scans. Neuroimage Clin. 2021 Jan 1;32. doi:10.1016/j.nicl.2021.102785 PubMed PMID: 34411910.

33. Salehinejad H, Kitamura J, Ditkofsky N, Lin A, Bharatha A, Suthiphosuwan S, et al. A real-world demonstration of machine learning generalizability in the detection of intracranial hemorrhage on head computerized tomography. Sci Rep. 2021 Dec 1;11(1). doi:10.1038/s41598-021-95533-2 PubMed PMID: 34426587.

34. Goyal R. Intracerebral Hemorrhage Detection in Computed Tomography Scans Through Cost-Sensitive Machine Learning. Applied Artificial Intelligence. 2022;36(1). doi:10.1080/08839514.2022.2138126

35. Alfaer NM, Aljohani HM, Abdel-Khalek S, Alghamdi AS, Mansour RF, Abdel-Khalek S. Fusion-Based Deep Learning with Nature-Inspired Algorithm for Intracerebral Haemorrhage Diagnosis. J Healthc Eng. 2022;2022. doi:10.1155/2022/4409336 PubMed PMID: 35087649.

36. Dyer T, Chawda S, Alkilani R, Morgan TN, Hughes M, Rasalingham S. Validation of an artificial intelligence solution for acute triage and rule-out normal of non-contrast CT head scans. Neuroradiology. 2022 Apr 1;64(4):735–43. doi:10.1007/s00234-021-02826-4 PubMed PMID: 34623478.

37. Gibson E, Georgescuphd B, Ceccaldi P, Trigan PH, Yoo Y, Das J, et al. Artificial Intelligence with Statistical Confidence Scores for Detection of Acute or Subacute Hemorrhage on Noncontrast CT Head Scans. Radiol Artif Intell. 2022 May 1;4(3). doi:10.1148/ryai.210115

38. Hopkins BS, Murthy NK, Texakalidis P, Karras CL, Mansell M, Jahromi BS, et al. Mass Deployment of Deep Neural Network: Real-Time Proof of Concept With Screening of Intracranial Hemorrhage Using an Open Data Set. Neurosurgery. 2022 Apr 1;90(4):383–9. doi:10.1227/NEU.0000000000001841 PubMed PMID: 35132970.

39. Altuve M, Pérez A. Intracerebral hemorrhage detection on computed tomography images using a residual neural network. Physica Medica. 2022 Jul 1;99:113–9. doi:10.1016/j.ejmp.2022.05.015 PubMed PMID: 35671679.

40. Kyung S, Shin K, Jeong H, Kim KD, Park J, Cho K, et al. Improved performance and robustness of multi-task representation learning with consistency loss between pretexts for intracranial hemorrhage identification in head CT. Med Image Anal. 2022 Oct 1;81. doi:10.1016/j.media.2022.102489 PubMed PMID: 35939912.

41. Gudadhe SS, Thakare AD, Oliva D. Classification of intracranial hemorrhage CT images based on texture analysis using ensemble-based machine learning algorithms: A comparative study. Biomed Signal Process Control. 2023 Jul 1;84. doi:10.1016/j.bspc.2023.104832

42. Cortés-Ferre L, Gutiérrez-Naranjo MA, Egea-Guerrero JJ, Pérez-Sánchez S, Balcerzyk M. Deep Learning Applied to Intracranial Hemorrhage Detection. J Imaging. 2023 Feb 1;9(2). doi:10.3390/jimaging9020037

43. Asif M, Shah MA, Khattak HA, Mussadiq S, Ahmed E, Nasr EA, et al. Intracranial Hemorrhage Detection Using Parallel Deep Convolutional Models and Boosting Mechanism. Diagnostics. 2023 Feb 1;13(4). doi:10.3390/diagnostics13040652

44. Abrigo JM, Ko KL, Chen Q, Lai BMH, Cheung TCY, Chu WCW, et al. Artificial intelligence for detection of intracraniahaemorrhage on head computed tomography scans: diagnostic accuracy in Hong Kong. Hong Kong Medical Journal. 2023 Apr 1;29(2):112–20. doi:10.12809/hkmj209053 PubMed PMID: 37088699.

45. Yeo M, Tahayori B, Kok HK, Maingard J, Kutaiba N, Russell J, et al. Evaluation of techniques to improve a deep learning algorithm for the automatic detection of intracranial haemorrhage on CT head imaging. Eur Radiol Exp. 2023 Dec 1;7(1). doi:10.1186/s41747-023-00330-3 PubMed PMID: 37032417.

46. Kang DW, Park GH, Ryu WS, Schellingerhout D, Kim M, Kim YS, et al. Strengthening deep-learning models for intracranial hemorrhage detection: strongly annotated computed tomography images and model ensembles. Front Neurol. 2023;14. doi:10.3389/fneur.2023.1321964

47. Umapathy S, Murugappan M, Bharathi D, Thakur M. Automated Computer-Aided Detection and Classification of Intracranial Hemorrhage Using Ensemble Deep Learning Techniques. Diagnostics. 2023 Sep 1;13(18). doi:10.3390/diagnostics13182987

48. Gamble C, Faghani S, Erickson BJ. Applying Conformal Prediction to a Deep Learning Model for Intracranial Hemorrhage Detection to Improve Trustworthiness. Radiol Artif Intell. 2025 Mar 1;7(2). doi:10.1148/ryai.240032

49. Li Y, Wu J, Li H, Li D, Du X, Chen Z, et al. Automatic detection of the existence of subarachnoid hemorrhage from clinical ct images. J Med Syst. 2012 Jun;36(3):1259–70. doi:10.1007/s10916-010-9587-8 PubMed PMID: 20827565.

50. Li YH, Zhang L, Hu QM, Li HW, Jia FC, Wu JH. Automatic subarachnoid space segmentation and hemorrhage detection in clinical head CT scans. Int J Comput Assist Radiol Surg. 2012;7(4):507–16. doi:10.1007/s11548-011-0664-3 PubMed PMID: 22081264.

51. Nishi T, Yamashiro S, Okumura S, Takei M, Tachibana A, Akahori S, et al. Artificial intelligence trained by deep learning can improve computed tomography diagnosis of nontraumatic subarachnoid hemorrhage by nonspecialists. Neurol Med Chir (Tokyo). 2021;61(11):652–60. doi:10.2176/nmc.oa.2021-0124 PubMed PMID: 34526447.

52. Thanellas A, Peura H, Lavinto M, Ruokola T, Vieli M, Staartjes VE, et al. Development and External Validation of a Deep Learning Algorithm to Identify and Localize Subarachnoid Hemorrhage on CT Scans. Neurology. 2023 Mar 21;100(12):E1257–66. doi:10.1212/WNL.0000000000201710 PubMed PMID: 36639236.

53. Yun TJ, Choi JW, Han M, Jung WS, Choi SH, Yoo RE, et al. Deep learning based automatic detection algorithm for acute intracranial haemorrhage: a pivotal randomized clinical trial. NPJ Digit Med. 2023 Dec 1;6(1). doi:10.1038/s41746-023-00798-8

54. Kyung S, Shin K, Jeong H, Kim KD, Park J, Cho K, et al. Improved performance and robustness of multi-task representation learning with consistency loss between pretexts for intracranial hemorrhage identification in head CT. Med Image Anal. 2022 Oct 1;81. doi:10.1016/j.media.2022.102489 PubMed PMID: 35939912.
